# The Specification and Global Reprogramming of Histone Epigenetic Marks during Gamete Formation and Early Embryo Development in *C. elegans*

**DOI:** 10.1371/journal.pgen.1004588

**Published:** 2014-10-09

**Authors:** Mark Samson, Margaret M. Jow, Catherine C. L. Wong, Colin Fitzpatrick, Aaron Aslanian, Israel Saucedo, Rodrigo Estrada, Takashi Ito, Sung-kyu Robin Park, John R. Yates, Diana S. Chu

**Affiliations:** 1 Department of Biology, San Francisco State University, San Francisco, California, United States of America; 2 Department of Chemical Physiology, The Scripps Research Institute, La Jolla, California, United States of America; 3 Mass Spectrometry Division, National Center for Protein Science Shanghai, Shanghai Institute of Biochemistry and Cell Biology, Chinese Academy of Science, Shanghai, China; 4 Department of Biochemistry, Nagasaki University School of Medicine, Nagasaki, Japan; IGBMC, France

## Abstract

In addition to the DNA contributed by sperm and oocytes, embryos receive parent-specific epigenetic information that can include histone variants, histone post-translational modifications (PTMs), and DNA methylation. However, a global view of how such marks are erased or retained during gamete formation and reprogrammed after fertilization is lacking. To focus on features conveyed by histones, we conducted a large-scale proteomic identification of histone variants and PTMs in sperm and mixed-stage embryo chromatin from *C. elegans*, a species that lacks conserved DNA methylation pathways. The fate of these histone marks was then tracked using immunostaining. Proteomic analysis found that sperm harbor ∼2.4 fold lower levels of histone PTMs than embryos and revealed differences in classes of PTMs between sperm and embryos. Sperm chromatin repackaging involves the incorporation of the sperm-specific histone H2A variant HTAS-1, a widespread erasure of histone acetylation, and the retention of histone methylation at sites that mark the transcriptional history of chromatin domains during spermatogenesis. After fertilization, we show HTAS-1 and 6 histone PTM marks distinguish sperm and oocyte chromatin in the new embryo and characterize distinct paternal and maternal histone remodeling events during the oocyte-to-embryo transition. These include the exchange of histone H2A that is marked by ubiquitination, retention of HTAS-1, removal of the H2A variant HTZ-1, and differential reprogramming of histone PTMs. This work identifies novel and conserved features of paternal chromatin that are specified during spermatogenesis and processed in the embryo. Furthermore, our results show that different species, even those with diverged DNA packaging and imprinting strategies, use conserved histone modification and removal mechanisms to reprogram epigenetic information.

## Author Summary

Successful reproduction depends upon the receipt and processing of distinct chromatin packages from sperm and oocytes. This includes not only a unique complement of DNA, but information in the form of proteins, such as histones, that differentially package the DNA in each cell type. For example, histone variants and post-translationally modified histones can carry epigenetic information across generations. Such information is then reprogrammed in the new embryo to ensure proper development. However, it is unclear how many of these marks are established during gamete formation and reprogrammed after fertilization. We define a signature histone variant and post-translational modification profile of sperm chromatin from *C. elegans*. This profile is established during sperm formation in part by exchanging canonical histones with sperm-specific histone proteins. Histone variants and modifications passed from sperm and oocytes are differentially removed or retained, suggesting that the embryo can reprogram epigenetic information from each parent distinctly. These *C. elegans* studies reveal that both conserved and novel histone modification and exchange mechanisms are used across diverse species to establish and reprogram epigenetic information.

## Introduction

The totipotency of a new embryo depends upon the reprogramming of epigenetic information carried over from the sperm and oocyte, each of which distinctly packages its DNA. For example, canonical histones H2A, H2B, H3, H4 and the linker histone H1 within mammalian sperm are replaced by sperm nuclear basic proteins (SNBPs) including histone variants, transition proteins, and protamines [1]–[4]. This repackaging results in global transcriptional repression and tight compaction of sperm chromatin [4]. After fertilization, sperm and oocyte chromatin also undergo distinct processes [5], [6]. Oocyte chromosomes complete meiotic divisions. Then, while maternal and paternal chromatin decondense, SNBPs are removed from sperm chromatin. Thus, distinct programs package the chromatin from each gamete type during their development and after fertilization. However, still largely unknown are the epigenetic features that are inherited, how they are reprogrammed in the new embryo, and the extent to which these reprogramming steps are conserved across species.

During sperm formation, histone variants are incorporated to restructure chromatin. In human, mouse, and fly, testes-specific variants of H1, H2A, H2B, and H3 displace canonical histones prior to incorporation of transition proteins and protamines [7]–[9]. *In vitro* studies demonstrate that some of these variants cause nucleosome destabilization [8], [10]–[15]. Other histone variants mark specific chromatin domains. For example in mouse spermatids, H2AL1/2 are incorporated into heterochromatic pericentric chromatin [14], inherited by the embryo, and removed upon paternal chromatin decondensation [16]. The fate of other paternally contributed histone variants remains unclear.

Chromatin restructuring during spermatogenesis is also regulated by histone post-translational modifications (PTMs). In *Drosophila* and mammals, global acetylation of histones precedes their replacement after meiosis [7], [17]–[21]. In particular, H4K16 acetylation (H4K16ac) targets histones for polyubiquitin-independent degradation within sperm-specific proteasomes called spermatoproteasomes [18]. During spermatogenesis in *Drosophila*, mouse, and rat, histone H2A is mono-ubiquitinated (H2AK119ub) [21]–[24]. However, the extent to which histone removal requires H2A ubiquitination by the ubiquitin ligase RNF8 remains controversial [23], [25]. Many other histone PTMs have been identified in sperm or testes but roles in the histone-to-protamine transition for most of these PTMs are unknown [26], [27].

Other roles for histone PTMs are as paternal imprints. In human sperm, marks of active transcription, such as H3K4me2 and H3K4me3, localize to paternally-expressed imprinted loci and to promoters of genes required for development, suggesting these paternal marks play key roles in developmental programs in the embryo [28]. Conversely, a mark of repressed chromatin, H3K27me3, is enriched at paternal promoters that are repressed in gamete formation and early embryos [28], [29]. In mouse, H4K8ac and H4K12ac, which mark heterochromatin regions in elongating spermatids, are also carried over by sperm [30], [31]. In *C. elegans*, the exclusion of H3K4me2 at inactive chromatin domains during spermatogenesis is maintained at those domains within the embryonic genome [32]. Thus histone PTMs can serve as epigenetic marks to transmit the history of specific chromatin regions to the new embryo [5], [33]. With the array of possible histone PTMs, there is significant potential for other paternal chromatin PTMs to play directive roles in the embryo.

In this work, we define the unique complement of epigenetic information contributed by histones in sperm chromatin in *C. elegans*. Histones are hypothesized to be the major constituent of epigenetic information in *C. elegans* because this organism lacks DNA methylation, which is classically associated with genomic imprinting in some species [34]. Though three highly homologous putative protamines, called SPCH-1, SPCH-2, and SPCH-3 (SPCH-1, 2, 3), have been identified in *C. elegans* [35], histones and histone PTMs are retained in sperm chromatin and delivered to the new embryo. For example, previous studies have detected canonical and variant histones [32], [35], GFP-labeled histone H3.3, or specific post-translationally modified histones (such as H3K4me2) in *C. elegans* sperm [32], [36]. However, a global profile of histone variants and PTMs in paternal chromatin and an understanding of their fate during the reprogramming of the embryonic genome are lacking. To address this, we employ a combination of approaches. First, we use proteomic and western analysis to define the histone profile of isolated sperm and compare it to the profile of mixed-stage embryos. Immunostaining is then used to track the temporal and spatial dynamics of how histone marks are established during spermatogenesis and oogenesis and then processed in the early embryo. This combination of approaches identifies paternal-specific epigenetic features and shows how parent-specific information can be retained or erased before and after fertilization.

## Results

### Histones are retained in *C. elegans* sperm chromatin

**Figure 1:**
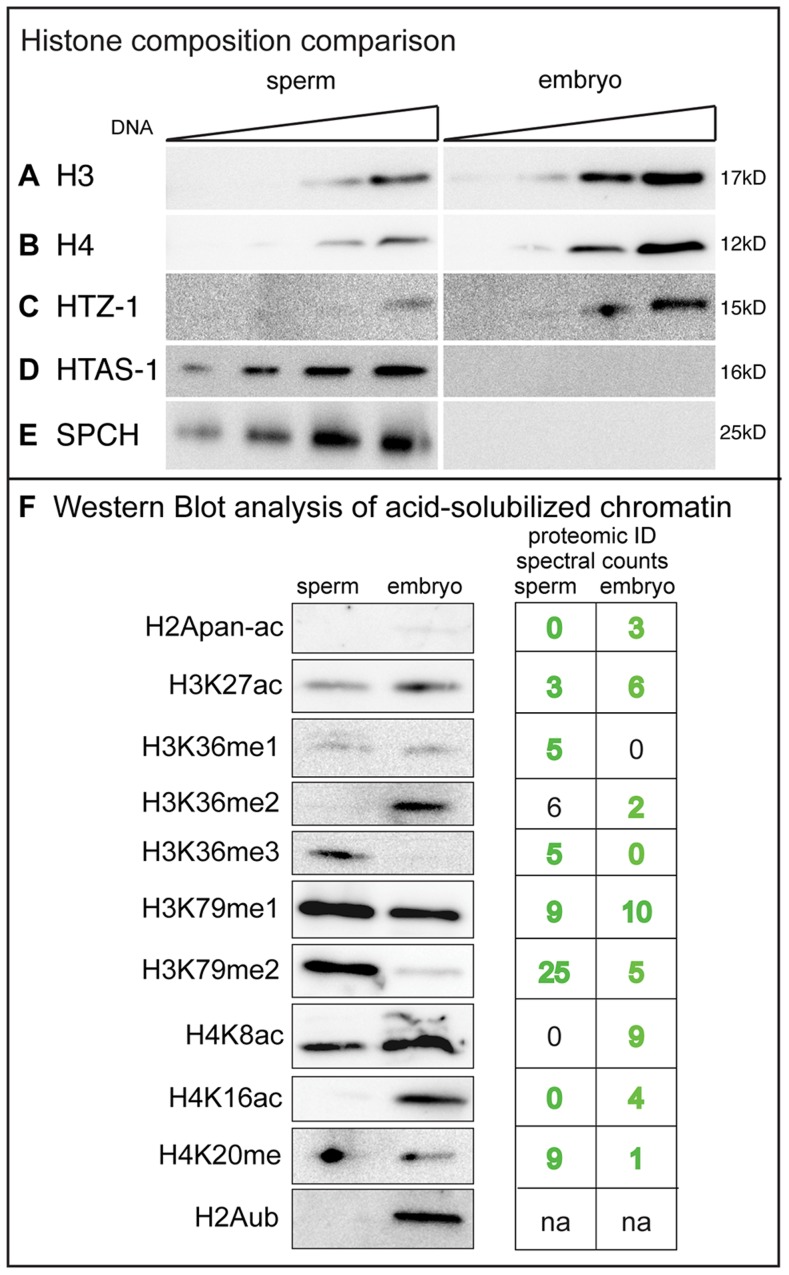
Canonical histones are retained in *C. elegans* sperm. **A–E**) Western blot analysis of sperm and embryo lysates loaded with equivalent amounts of DNA. **A**) Histone H3: 12, 24, 60 and 120 ng; **B**) Histone H4: 24, 48, 120, and 240 ng; **C**) HTZ-1: 24, 48, 120, and 240 ng; **D**) HTAS-1: 12, 24, 60 and 120 ng; **E**) SPCH-1, 2, 3: 12, 24, 60 and 120 ng. Bands shown were quantified using ImageJ software (see Methods). **F**) Western analysis detection of histone post-translational modification (PTMs) in acid-solubilized sperm and embryo chromatin samples. The left panel shows western signals for the histone PTMs indicated. Right panels show spectral counts for histone PTMs identified by MuDPIT analysis (from Table 1). Green text indicates western and proteomic identification correspond. Black text indicates western and proteomic identification did not correspond. ‘na’ is not applicable because identification by proteomics of ubiquitination was not included in PTM counts (see Methods). The anti-H2AK119ub antibody (#87, see Table S7) recognizes the corresponding residue K120 in *C. elegans* (File S5).

To assess the extent of histone retention in sperm, we compared histone levels in sperm (a relatively uniform population of transcriptionally inactive, differentiated haploid cells [37]) to that of mixed-stage embryos (transcriptionally active and developing diploid cells). Western blot analysis shows canonical histone H3, H4, and the ubiquitous H2A variant HTZ-1 are present in both sperm and embryos, while the H2A variant HTAS-1 and the putative protamines SPCH-1, 2, 3 are found only in sperm (Figure 1A–E). Quantification revealed that lysates from sperm possess roughly 37% of canonical histones H3 and H4 compared to those of embryos that contain equivalent amounts of DNA (Figure 1A, B, Methods). HTZ-1 was found at 30% in sperm lysates compared to embryo lysates (Figure 1C, Methods). Thus, *C. elegans* retain a considerable portion of histones in sperm compared to humans (∼4–15%), mice (∼1%), and *Drosophila* (∼0%) [21], [28], [38], [39].

To further explore the histone content of chromatin from sperm and embryos we employed Multidimensional Protein Identification Technology (MudPIT), a sensitive approach that can detect low-abundance chromatin proteins and PTMs in complex mixtures of proteins [40]–[43]. We purified and acid solubilized chromatin from sperm and mixed-stage embryos [44]. SDS PAGE analysis revealed histones H2A, H2B, H3, and H4 were enriched in both chromatin samples (Figure S1A, B). These samples were digested with proteases and then analyzed by MudPIT. Spectra from each sample were matched by the ProLuCID algorithm to predicted peptides and assembled into corresponding *C. elegans* proteins by the DTASelect program [45]–[47]. Consistent with SDS PAGE and western analysis (Figure 1A–D, Figure S1A, B), histones were among the most highly represented from the 1889 spermatogenic and 2421 embryonic proteins identified as evaluated by either total spectral counts, normalized spectral abundance factor (NSAF) values, or Exponentially Modified Protein Abundance Index (EMPAI) values (Table S1, Table S2) [41], [48]–[50].

### The global profile of histone variant and post-translational modifications in sperm and embryo chromatin is distinct

We looked for chromatin proteins enriched in embryo or sperm chromatin samples compared to one another utilizing the spectral count feature of MudPIT, which gives an idea of the relative levels of more abundant proteins [41], [51], [52]. Indeed, we found that the H2A variant HTAS-1 is highly enriched in sperm chromatin with 285 spectral IDs out of 3595 total H2A spectra IDs (which include modified and unmodified canonical H2A, HTZ-1, and HTAS-1 spectra) compared to embryo chromatin (0/4758 total H2A spectra IDs) (Table S3). Furthermore, MudPIT analysis was sensitive: we were able to detect 36 different histone PTMs including mono-methylated (me1), di-methylated (me2), tri-methylated (me3) and acetylated (ac) residues (Table 1, Tables S3–S6, Files S1–S4). Few phosphorylated residues were found, likely because phosphorylation is inherently labile and phospho-enrichment methods were not used. The coverage of the N- and C-termini of histones was also low because they are rich in lysine and arginine, the recognition sites of the proteases used.

**Table 1:** Post-translational modification sites of core histone proteins identified by MudPIT analysis in embryos and sperm.

Type	*C. elegans* Site	Mouse Site	Sperm				Embryo			
			ac	me1	me2	me3	ac	me1	me2	me3
H2A	K5	K5					3			
	K8	K9					3			
	K10	R11					3			
	K96	K95	1				2			
	K119	K118					6			
H2B	K7	K12					9			
	K10	K15	1				20			
	K11	K16	1				25			
	K14	K20	3				63			
	K21	K24					6			
	K105	K108					3			
	K113	K116					1			
H3	K9	K9	1							
	K14	K14	2				3			
	K23	K23	2	1	6	5	3	2	44	4
	K27	K27	3				6		16	47
	K36	K36	34	5	6	5	11		2	
	K79	K79		9	25			10	5	
H4	K5	K5	1				1			
	K8	K8					9			
	K12	K12	1				9			
	K16	K16					4			
	K20	K20		9	13			1		
	K31	K31					3			
	K59	K59					1			
	K79	K79	1							

Amino acid numbering (starting at the amino acid after the starting methionine, see File S5) following the convention of the histone field is shown for *C. elegans* and the corresponding site in mouse. The number of occurrences (see Methods) of acetylation (ac), methylation (me), di-methylation (me2), and tri-methylation (me3) are shown for identified modification sites from each histone subtype.

Overall, proteomic analysis found 31 histone PTM marks in embryos compared to 22 marks in sperm (Table 1), some of which would not have been identified by immunostaining or western analysis because antibodies that recognize them are not available. Our analysis also found ∼2.4 fold fewer modified peptides in sperm samples (127 PTM histone spectra IDs/7190 total histone spectra IDs = 1.8%) compared to embryo samples (253 PTM histone spectra IDs/5998 total histone spectra IDs = 4.2%) (Tables S3, S4, S5, S6, Methods). A comparison of histone PTMs between sperm and embryos revealed differences in the identification of some PTM classes in each histone subtype. In general, acetylation on histone H2A, H2B, and H4 were identified more frequently and on more sites in embryos compared to sperm, whereas methylation on histone H3 was detected at similar levels in both samples (Table 1).

To further investigate the presence of histone PTMs identified by proteomic analysis, we applied western analysis on sperm and mixed-stage embryo chromatin. One drawback of conducting westerns is the availability, reliability, and expense of suitable antibodies [53]. Indeed, of 27 available antibodies to modified and unmodified histones we screened (Table S7), 12 recognized bands at the expected molecular weight (Table S7, Figure 1F). A band was detected in 14/16 instances for sites where proteomic analysis had also identified the histone PTM (Figure 1F); therefore, proteomic identification is suitable for detecting both high and low abundance histone PTMs. However, in 2/4 instances western analysis detected a band for sites where proteomic analysis did not identify a modification (Figure 1F). Hence, the lack of detection by either proteomics or westerns is not in itself definitive evidence that the modification is absent. An antibody that recognizes ubiquitinated histone H2A (H2Aub) [(at lysine K119 in mammals, which corresponds to *C. elegans* K120 (File S5)] also detected H2Aub in embryos but not sperm (Figure 1F) [54]. Thus, both proteomic and western analysis support that the global histone variant and PTM profile of sperm is distinct from that of mixed-stage embryos.

### The histone H2A variant HTAS-1 exclusively marks paternal chromatin

**Figure 2:**
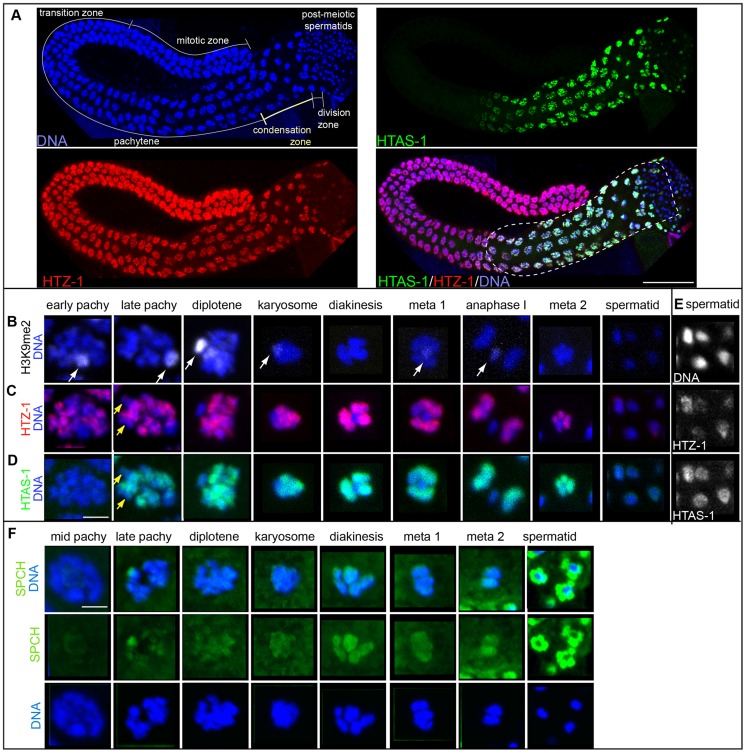
HTAS-1 is incorporated as sperm chromosomes condense. Immunolocalization of isolated and fixed *C. elegans* male gonads. **A**) DNA stained with DAPI (blue), HTAS-1 (green), HTZ-1 (red), merged image of HTAS-1 (green), HTZ-1 (red) and DAPI (blue). Scale bar represents 50 µm. **B–E**) Immunostaining of individual nuclei from late stages of sperm formation (as found in the proximal end of the gonad marked with the dotted line in A). The scale bar represents 2 µm and applies to all panels in B–F. **B**) Histone H3 dimethylated at lysine 9 (H3K9me2), which marks the X chromosome (white arrows). **C**) HTZ-1. **D**) HTAS-1. Yellow arrows mark regions of under-representation of HTAS-1 and HTZ-1 that are not the X chromosome. **E**) HTZ-1 and HTAS-1 are detectable immediately after meiotic divisions as sperm chromatin condenses. Contrast-adjusted black and white images of DNA, HTZ-1, and HTAS-1 staining of the early spermatid nuclei shown in panels C and D that show HTZ-1 and HTAS-1 are detectable. See Figure S2 for contrast-adjusted images of later spermatid nuclei. **F**) SPCH proteins (SPCH-1, 2, 3) (green) are detectable on DNA as sperm DNA condenses for meiotic divisions. Levels of SPCH proteins increase dramatically after meiosis, particularly around spermatid DNA.

Because proteomic and western analysis on sperm and embryo chromatin indicated HTAS-1 is enriched in sperm (Table S3, Figure 1D), we verified it is sperm-specific. First, HTAS-1 is present in mutant strains that produce only sperm and not in those that produce only oocytes, similar to the sperm-specific Major Sperm Protein (MSP) (Figure S1C). In contrast, another H2A variant, HTZ-1, was detected in both (Figure S1C). Furthermore, immunolocalization on dissected males and hermaphrodites found that HTAS-1 is expressed only during spermatogenesis in both sexes (Figure 2A, Figure S2A) [35]. HTAS-1 incorporation into chromosomes starting from the late pachytene stage precedes incorporation of the putative protamines SPCH-1, 2, 3 (Figure 2D, F). HTAS-1 is strongly enriched on condensing DNA. It is also detectable on all post-meiotic spermatids when SPCH-1, 2, and 3 levels are high. In contrast, HTZ-1 and histone H1 are present at all stages (Figure 2A, F, Figure S2B). Thus, from proteomic, western, and cytological analysis, we conclude that HTAS-1 is a sperm-specific chromatin protein.

HTAS-1 incorporates into chromosomes as they condense and transcription becomes globally reduced (Figure 2A) [55], [56]. We were thus interested in determining the localization of HTAS-1 relative to HTZ-1, which incorporates at promoters and regulates transcription in embryos [57]–[59]. Though HTZ-1 is present in all cells, several chromosomal regions exhibit relatively low levels of HTZ-1, particularly the silenced and condensed X chromosome, which has been shown to be marked by dimethylation of lysine 9 on histone H3 (H3K9me2) in male germ lines (Figure 2B, C) [60]–[63]. Levels of HTZ-1 staining do not substantially diminish as HTAS-1 is incorporated, suggesting that the function of HTAS-1 is not solely to displace HTZ-1. Instead, HTAS-1 is integrated into similar chromosomal regions as HTZ-1 (Figure 2C and D) and both are retained on post-meiotic spermatids (Figure 2E). Staining of HTZ-1 and histone H1, which was previously found to be retained in sperm [64], decreases in later spermatids (Figure S2B); however, both are visible on paternal chromatin in newly fertilized embryos (Figure S3A), supporting the idea that they are retained in sperm and carried over at fertilization [55], [56].

**Figure 3:**
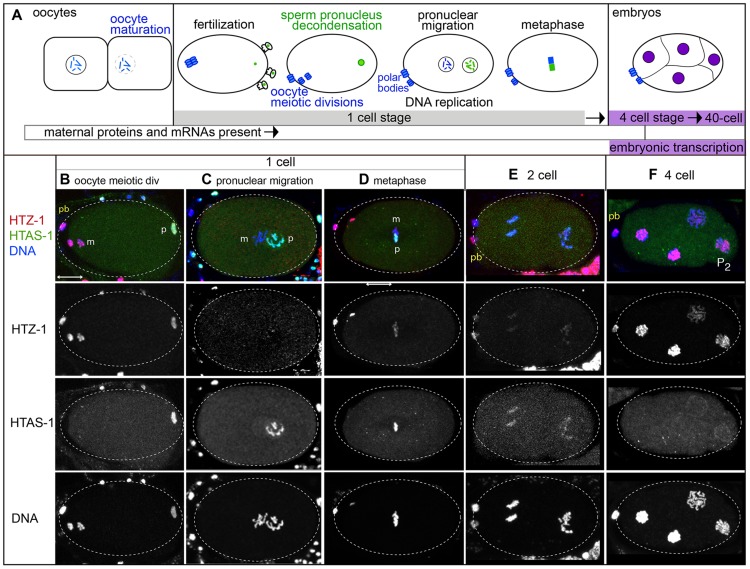
Histone H2A variants have different fates in the new embryo. **A**) A schematic of paternal and maternal chromatin processing before and after fertilization. **B–F**) Immunostaining of embryos in different stages show that HTAS-1 (green in merged images) is retained in paternal (p) chromatin while HTZ-1 (red) is removed from paternal and maternal (m) chromatin in the 1-cell embryo. DNA is shown in blue. The scale bar represents 10 µm and applies to all panels. **B**) 1-cell embryos after fertilization. The oocyte pronucleus is undergoing meiotic divisions while the paternal pronucleus is decondensing. **C**) After DNA replication, the pronuclei migrate to the center of the embryo. **D**) Chromosomes align on the metaphase plate before segregation. **E**) 2-cell embryos **F**) 4-cell embryos. P_2_ marks the transcriptionally silent P_2_ germline precursor cell.

In the new embryo, differences in histone variant dynamics were found by examining chromatin transitions unique to each gamete of origin (Figure 3A). Prior to fertilization, oocytes adjacent to the spermatheca mature, a process that includes nuclear envelope breakdown, nuclear migration, and cortical rearrangements [65]. After sperm entry, the oocyte pronucleus completes meiotic divisions to produce a haploid complement of maternal DNA and two polar bodies [6], [66]. Both the oocyte and sperm chromatin then decondense, become ensheathed within pronuclear envelopes, and undergo DNA replication [67]. The oocyte and sperm pronuclei migrate, transition to metaphase and undergo mitosis. Embryos in the 1- to 2-cell stage rely on maternal stores of proteins and mRNAs because embryonic transcription does not initiate until the 4-cell stage [68], [69]. Embryos complete transfer from maternal to embryonic control of development around the 40-cell stage.

### After fertilization, paternally-delivered H2A variants have different fates

Interestingly, histone H2A variants have different fates in the embryo. HTZ-1 levels are high on both paternal and maternal chromatin just after fertilization (Figures 3B, Figure S3A), but levels drop on both after oocyte meiotic divisions (Figure 3C, D). Thus HTZ-1 is either removed by maternally loaded factors or displaced during DNA replication. HTZ-1 levels remain low in 1- and 2-cell stage embryos (Figures 3C–E and S3A) then rise in the 4-cell stage on all nuclei (Figure 3F). This includes the transcriptionally silenced germline precursor P_2_ cell [70], [71], suggesting embryonic transcription is not necessary for HTZ-1 incorporation. In contrast, HTAS-1 is retained on paternal chromatin and absent on maternal chromatin throughout the 1-cell stage (Figure 3B–D, Figure S3B). HTAS-1 marks only the paternal half of the chromatin mass at metaphase, revealing the striking compartmentalization of chromosomes at this stage (Figure 3D). After division, HTAS-1 is detectable on chromosomes and levels subsequently decline with each cell division, becoming undetectable after the 4-cell stage (Figure 3E, F). Thus, HTZ-1 is removed after fertilization while HTAS-1 is retained. These results show that histone H2A variants are differentially processed in the early embryo and indicate distinct mechanisms recognize and either remove or retain each variant.

### H2A ubiquitination correlates with histone exchange

The dynamics of HTZ-1 and HTAS-1 before and after fertilization suggests histone subunit exchange during these critical transitions. A clue about the mechanism of this process was revealed by western blot analysis, which detected H2Aub in chromatin from embryos but not sperm (Figure 1F) [54]. To investigate this difference further, we tested four antibodies via immunostaining in *C. elegans* that were previously shown to specifically recognize H2Aub in mammals (Table S7) [54], [72], [73]. Of these, one antibody, the monoclonal E6C5 antibody detected H2Aub in both male and hermaphrodite germ lines but not in later stages of spermatogenesis (Figure S4A). During the pachytene stage, H2Aub is found on all chromosomes (Figure S4A), which is in contrast to the heterochromatic sex body localization observed at that stage in mice [22], [74]. As H2Aub levels on chromosomes decrease in early pachytene, the E6C5 antibody also detected foci not associated with DNA, a pattern we subsequently refer to as “off-chromatin foci” (Figure S4A) that are distinct from germline P-granules [75]. The H2Aub off-chromatin foci do share partial overlap with poly-ubiquitin conjugates linked through either lysine 48 (Ub-K48), which target proteins to the proteasome, or lysine 63 (Ub-K63), which can act as a signal for autophagy (Figure S4B–D) [76]. The overlapping localization of Ub-K48 and the proteasome together with the presence of Ub-K63 foci suggest these pathways are active during late spermatogenesis, when chromatin is undergoing extensive remodeling. The lack of H2Aub on spermatid chromatin is consistent with western blot analysis and, together with the potential overlap of off-chromatin foci with ubiquitin conjugates, suggests H2A may be removed during late spermatogenesis.

**Figure 4:**
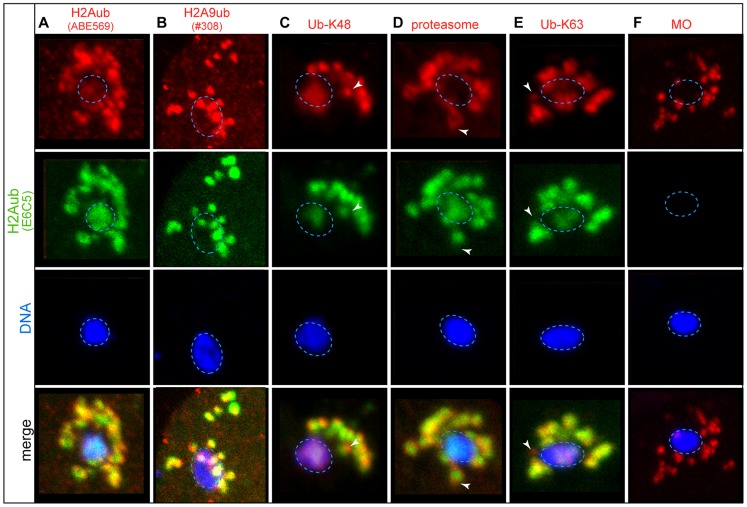
Paternal histone H2A is ubiquitinated and removed after fertilization. Immunostaining of fixed 1-cell embryos. The scale bar represents 10 µm and applies to all panels. The paternal DNA (blue dotted ovals) stained with the DNA dye DAPI (blue) and the monoclonal E6C5 antibody that recognizes H2Aub (green) [73] and the following (in red): **A**) H2Aub recognized by the ABE569 antibody [72]. **B**) H2Aub recognized by the polyclonal #308 antibody [54]. **C**) K48-linkage specific polyubiquitin (Ub-K48) that can target proteins for degradation via the **D**) proteasome. **E**) K63-linkage specific polyubiquitin (Ub-K63) tags targets, like **F**) Membranous Organelles (MOs), for autophagy [76]. The white arrowheads mark regions of staining that does not overlap with H2Aub staining. The overlapping staining suggests that H2Aub may be processed by either autophagy (like MOs) or by the proteasome after fertilization.

Because spermatids lack the H2Aub mark before fertilization, we were surprised to observe high levels of H2Aub on paternal chromatin and in adjacent off-chromatin foci in newly fertilized embryos using either the E6C5 monoclonal or the polyclonal ABE569 and #308 anti-H2Aub antibodies (Figure S5, Figure 4A, B). Similar structures around paternal DNA were previously found to harbor sperm membranous organelles (MOs) that become poly-ubiquitinated on lysines 63 and 48 to target them for destruction by autophagy and possibly the proteasome after fertilization (Figure 4C–F) [76], [77]. H2Aub staining partially overlaps with Ub-K48 and Ub-K63 staining on paternal DNA and in some off-chromatin foci as oocyte chromosomes complete meiosis (Figure 4C–E) and is distinct from the localization of MOs both before and after fertilization (Figure 4F). The partially overlapping regions of staining with Ub-K48 and Ub-K63 suggest that paternally-delivered H2A may, like MOs, be targeted for destruction after fertilization [76]. Maturing and meiotically-dividing oocyte chromosomes also exhibit low H2Aub levels, where off-chromatin foci were also visible (Figure S5). After oocyte meiosis, levels of chromatin-associated H2Aub fall (Figure S5). By the 16-cell stage, H2Aub chromosomal staining rebounds (Figure S5) but Ub-K48 and Ub-K63 staining are absent [77]. Together, these results indicate that H2A may be removed and targeted for destruction by ubiquitination as paternal and maternal chromosomes decondense, suggesting that ubiquitination of H2A plays a role in H2A exchange in the newly fertilized embryo.

### Lack of bulk histone acetylation distinguishes the paternal from the maternal pronucleus in the newly fertilized embryo

The differential acetylation profile of sperm chromatin was next examined by testing commercially available antibodies to find those that recognized specific chromatin patterns in *C. elegans* (Table S7). We found that 8 acetylated histone marks [H4K16ac, H2BK12ac, H3K23ac, H3K27ac, H4K5ac, H4K8ac, H4K12ac, and H2A acetylated on sites K5, K9, K13, K15 (hereafter referred to as H2Apan-ac)] dramatically decrease during sperm chromosome condensation (Figure S6, Table S7, File S5). Six of the sites (H3K27ac, H4K5ac, H4K12ac, H4K16ac, H2Apan-ac, and H2BK12ac) showed no staining on nuclei undergoing the second meiotic division (Figure S6A–F), while 2 sites - H4K8ac and H3K23ac - were detectable at low levels on metaphase 2 nuclei (Figure S6G, H). Thus, overall acetylation levels are decreased on post-meiotic sperm, consistent with proteomic results (Table 1).

### Bulk histone acetylation status becomes equalized before the first mitotic division

**Figure 5:**
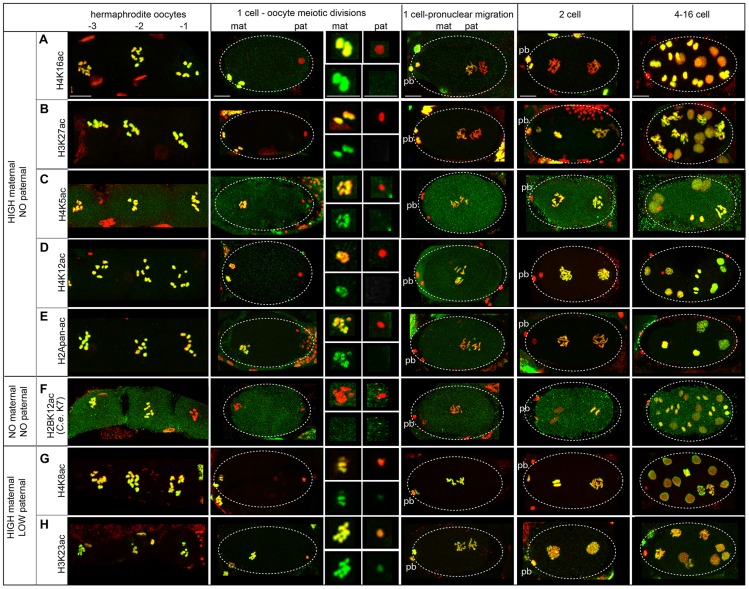
Paternal and maternal chromatin differ in acetylation status after sperm entry. Immunostaining of dissected and fixed hermaphrodite gonads with antibodies against acetylated histones (green) and DNA stained with DAPI (red). Before fertilization, oocytes are numbered with the −1 oocyte adjacent to the spermatheca. Top insets from 1-cell embryos undergoing oocyte meiotic divisions show merged images of enlarged maternal and paternal pronuclei. Bottom insets show acetylated histone staining only. Polar bodies are denoted by ‘pb’, ‘mat’ is maternal, ‘pat’ is paternal. Scale bars represent 10 µm for all panels. **A**) H4K16ac levels are high in oocytes and the female pronucleus after fertilization, but absent on that paternal pronucleus. Levels are low until the 4-cell stage and are strong by the 16-cell stage. **B**) H3K27ac is present in oocytes and the female pronucleus but absent on the paternal pronucleus after fertilization. Levels increase gradually to become high in 2-cell embryos. **C**) H4K5ac **D**) H4K12ac and **E**) H2Apan-ac levels are strong in oocytes and the female pronucleus during meiotic divisions but are absent on sperm. Levels increase to become high in 2-cell embryos. **F**) H2BK12ac (corresponding to H2BK7 in *C. elegans*, see File S5) is not detectable in oocytes adjacent to the spermatheca or on maternal and paternal pronuclei after fertilization. Levels increase in 4-cell embryos. **G**) H4K8ac and **H**) H3K23ac are strong on maternal chromatin and weak, but present, on paternal pronuclei. Levels are high on both after meiotic divisions.

Immediately after fertilization, the lack of staining for 5 sites (H4K16ac, H3K27ac, H4K5ac, H4K12ac, H2Apan-ac) on paternally-derived chromatin distinguishes it from the maternal chromatin, where high levels of staining were observed (Figure 5A–E, Figure S3C–E). In the early embryo, acetylation dynamics reveal differences in the fate of different PTMs on each pronucleus and mechanisms of histone acetylation/deacetylation.

In particular, H4K16ac staining is retained on the maternal chromosomes but excluded from the paternal chromosomes in 1-cell embryos (Figure 5A, Figure S3C). Levels of H4K16ac remain low in 1- to 2-cell stage embryos but increase dramatically in 4-cell stage embryos in all cells, including the P_2_ cell, suggesting transcription is not necessary for H4K16 acetylation (Figure S7A). Overall, the sperm-specific loss of H4K16ac results in a maternal-specific H4K16 acetylation mark in 1-cell embryos.

Acetylation dynamics of H3K27 suggest that some histone acetylases are maternally loaded. H3K27ac levels are high on maternal chromatin after fertilization, but absent on paternal chromatin (Figure 5B). However, H3K27ac levels on paternal chromatin gradually rise until staining is strong in 2-cell embryos. This gradual rise in 1-cell embryos suggests maternal loading of the acetylase for this site, resulting in an “equalization” of H3K27ac levels between paternal and maternal pronuclei in the 1-cell embryo.

A general equalization of acetylation levels can occur via histone deposition during DNA replication. Histone H4 is marked by di-acetylation at K5 and K12 as it incorporates during DNA replication in *Tetrahymena*, *Drosophila*, and human cells [78]. Indeed in *C. elegans*, we also found H4K5ac and H4K12ac are present on the maternal pronucleus but absent on paternal chromatin after fertilization; however, levels of each are uniform on both pronuclei after DNA replication (Figure 5C, D, Figure S3D). The timing of this equalization suggests these marks may be added to the paternal chromatin via histone deposition during DNA replication. An antibody that recognizes multi-acetylated H2A (H2Apan-ac) on K5, K9, K13 and K15 (the only antibody we tested against H2A that produced positive immunostaining results, see Table S1 and File S5) exhibited similar dynamics (Figure 5E, Figure S3E). H2AK5 acetylation is influenced in the germline by XND-1, a protein involved in regulating meiotic crossovers in *C. elegans* [79]; however, little is known about acetylation of on these residues in any organism.

Deacetylation in oocytes before fertilization can also contribute to equalizing acetylation levels. Oocytes closest to the spermatheca, which are maturing [65], exhibit a dramatic drop in H2BK12ac levels on chromosomes during diakinesis (Figure 5F, Table S7, File S5), suggesting that H2BK12 deacetylation could be triggered by oocyte maturation signals. However, we also observe deacetylation of H2BK12 also occurs at diakinesis in spermatocytes (Figure S6F). H2BK12ac levels remain low on both sperm and oocyte chromatin until the 2–4 cell stage, when H2BK12ac then decorates all nuclei including the P_2_ cell (Figure S7B), suggesting that embryonic transcription is not directly correlated with its presence [71].

Epigenetic acetylation marks present at different levels in both pronuclei at sperm entry can become equalized. H4K8ac and H3K23ac exhibit high levels of staining in oocytes and low levels in late stages of sperm meiosis (Figure 5G, H, Figure S6G, H). Consistent with this, post-fertilization H4K8ac and H3K23ac staining is much weaker on paternal chromatin compared to maternal chromatin (Figure 5G, H). H4K8ac levels on both pronuclei increase dramatically during oocyte meiotic divisions, while H3K23ac levels remain modest in 1-cell embryos but become strong in 2-cell embryos. The increase in acetylation of both sites indicates that the acetylases that recognize each are likely to be maternally loaded.

Overall, we have identified features of paternal and maternal chromatin histone acetylation dynamics before and after fertilization in *C. elegans* that are both shared and distinct from that of other organisms. These observations show that 1) paternal chromatin undergoes erasure of many acetylation sites during sperm formation, 2) sperm and oocyte chromosomes carry parent-specific markings into the new embryo, 3) paternal and maternal pronuclei can undergo distinct histone remodeling events both before and after fertilization, 4) the processing of epigenetic information is dependent on maternal loading of histone modification enzymes.

### Retention of histone methylation in sperm marks specific chromatin domains in embryos

Parent-specific imprinting in *C. elegans* can be specified by histone methylation that marks the transcriptional activity of chromatin domains by two different mechanisms [32], [61], [80]. Meiotic silencing of unpaired chromatin (MSUC) is implemented in part by excluding H3K4me, a mark of germline transcriptional activity, from the single X chromosome in XO males [61]. The silenced male X chromosome instead harbors H3K9 methylation marks [61]–[63], [81]. After fertilization, only the paternal X remains resistant to H3K4 methylation while autosomes and the maternal X exhibit this mark. In contrast, H3K36me2 is a different mark added by the MES-4 methyltransferase only on autosomes in hermaphrodites and males to transmit a memory of gene expression patterns from the parental germ line to germline precursors in offspring [80], [82]. Thus H3K36me2 is absent on both the paternal and maternal X chromosomes before and after fertilization. Our proteomic analysis identified other histone methylation marks on sperm chromatin (Table 1). We used immunostaining to investigate the retention of three methylation marks (H3K36me1, H4K20me1, and H3K79me2) with available commercial antibodies (Table S7).

**Figure 6:**
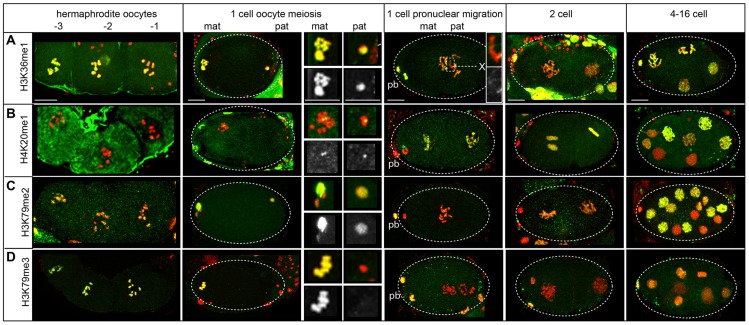
Histone methylation dynamics on the paternal and maternal chromatin in the embryo. Immunostaining of dissected and fixed hermaphrodite gonads with antibodies specific to methylated histones (green) and DNA stained with DAPI (red). Before fertilization, oocytes are numbered with the −1 oocyte adjacent to the spermatheca. Polar bodies are denoted by ‘pb’, ‘mat’ is maternal, ‘pat’ is paternal. Scale bars represent 10 µm for all panels. **A**) H3K36me1 levels are high on both pronuclei after fertilization but absent on the paternal X chromosome, which is shown enlarged in the inset (top is merged image, bottom is H3K36me1 staining shown in grayscale). **B**) H4K20me1 is detectable as small foci on both pronuclei after fertilization with levels increasing on all chromosomes following oocyte meiosis. **C**) H3K79me2 levels are high on both pronuclei, low in 1 to 4-cell stage embryos, then high after the 16-cell stage. **D**) H3K79me3 is present on the maternal pronucleus but not the paternal after fertilization. Levels of H3K79me3 are very low after oocyte meiotic divisions and rise again after the 8-cell stage.

We found H3K36me1 is likely specified by mechanisms that implement MSUC and not by MES-4. During spermatogenesis in males, H3K36me1 is absent from the single X chromosome but present on autosomes in nuclei at all stages, though low on post-meiotic sperm chromatin (Figure S8A). During oogenesis in hermaphrodites, all chromosomes, including the maternal X, stain with H3K36me1 at the diplotene stage (Figure 6A). In early embryos, only the paternal pronuclei lack one region of H3K36me1 staining, which, because it lacked staining before fertilization, is presumably the paternal X chromosome. This pattern is distinct from that of H3K36me2 [80], [82] and instead mirrors the staining pattern observed after fertilization for H3K4me [61], [62]. Thus, the dynamics of H3K36me1 we observe more closely correlates with the imprinting mechanisms of MSUC and not MES-4 [61], [80].

H4K20me1 exhibits a distinctive localization pattern when carried over to the embryo. In *C. elegans*, though H4K20me1 is enriched on dosage-compensated X chromosomes in somatic cells, it is reduced on meiotically-silenced X chromosomes during the pachytene stage [83]. We observe that by diplotene/diakinesis, H4K20me1 becomes focused in one or two bright chromatin-associated spots (Figure 6B). In male sperm-producing germ lines, levels of H4K20me1 are also low on the single X chromosome during pachytene (Figure S8B) then fall; however, one or two bright chromatin-associated foci appear. Costaining with an antibody that recognizes H3K9me2, a marker for the X chromosome [61], [62], [81], show that these foci are not on X (Figure S8B). After fertilization, the paternal and maternal pronuclei are similarly decorated with small H4K20me1 foci. Levels then rise to cover all chromosomes in both pronuclei (Figure 6B), suggesting the H4K20 methylase is maternally loaded. These results show that H4K20me1 is retained on sperm chromatin in a novel localization pattern and demonstrate that paternal and maternal nuclei are equivalent in H4K20me1 status in the new embryo.

H3K79me2 is also carried over to the new embryo from both sperm and oocytes (Figure S8C, Figure 6C). H3K79me2 is found at high levels on oocyte chromosomes before and after fertilization. However, though H3K79me2 status is equivalent in paternal and maternal pronuclei after sperm entry; levels drop in 1-cell embryos (Figure 6C), suggesting a H3K79 demethylase is maternally loaded to reprogram paternally delivered marks in the new embryo. A similar demethylation of H3K79 also occurs in mouse [84]. H3K79me2 levels rise in 4-cell embryos, including the transcriptionally inactive P_2_ cell (Figure S7C), indicating its presence is not dependent on transcription [71].

Not all histone methylation marks are retained in sperm chromatin. H3K79me3 was not identified in sperm chromatin by proteomic analysis. In the male sperm-producing germ lines, H3K79me3 levels fall before meiotic divisions (Figure S8D). In contrast, H3K79me3 levels are high in oocyte-producing germ lines through diakinesis. Subsequently, lack of H3K79me3 distinguishes the paternal pronucleus from the maternal pronucleus after fertilization (Figure 6D). Levels of H3K79me3 become low in both maternal and paternal pronuclei then rise after the 16-cell stage. This suggests that the enzyme responsible for H3K79me3 is likely not maternally supplied. The removal of maternal H3K79me3 in early embryos was similarly observed in mouse early embryos [84].

## Discussion

**Figure 7:**
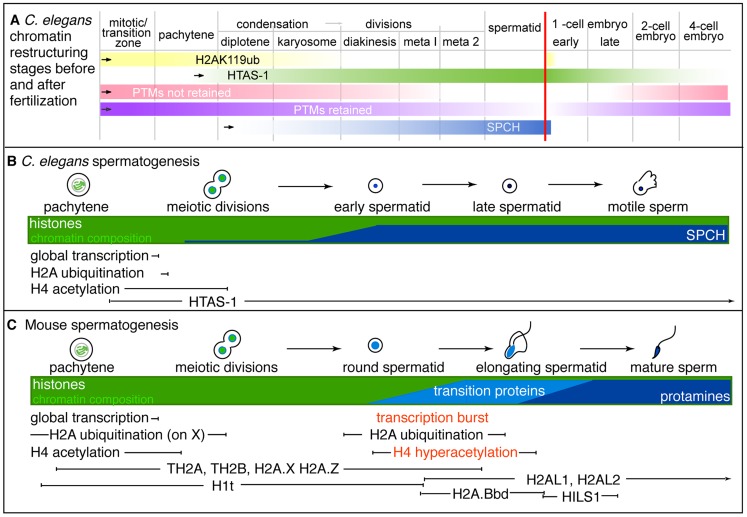
Summary of stages of chromatin remodeling events in *C. elegans* during spermatogenesis and post-fertilization. **A)** Schematic of overlapping stages that restructure sperm chromatin to establish paternal epigenetic information during spermatogenesis. Bars represent the presence and levels of key markers, the vertical red line represents the point fertilization occurs. The stages include: 1) H2Aub removal from chromatin (yellow) as HTAS-1 (green) is incorporated and retained in the new embryo. 2) Removal of many histone PTMs (pink) prior to meiotic divisions that result in the erasure of paternal epigenetic marks. Some histone PTMs are retained (purple). 3) Putative *C. elegans* protamines, SPCH-1, 2, 3 (SPCH, blue) begin incorporating during meiosis and are found at high levels in spermatids. **B** and **C**) Schematic comparison of key events during spermatogenesis between **B**) *C. elegans* and **C**) mouse (inspired by [92]). Notably, HTAS-1 is incorporated and retained in *C. elegans* while some histone variants are transiently incorporated to facilitate transition and protamine incorporation in mouse. Also, the post-meiotic transcriptional burst and histone hyperacetylation seen during mouse spermatogenesis are not observed during *C. elegans* spermatogenesis.

In this study, we define the epigenetic profile of *C. elegans* sperm chromatin. This distinctive profile includes (1) sperm-specific incorporation of the histone H2A variant HTAS-1, (2) a depletion of histone acetylation marks, and (3) the retention of specific histone marks, like H3K36me1, that convey the transcriptional history of chromatin domains. Overall, we find that sperm generally harbor less histone PTMs on fewer sites compared to embryos and tracked 13 sites via immunostaining before and after fertilization to define distinctive features of paternal and maternal chromatin (Tables 1, 2). An important product of these studies is a set of temporal markers of the dramatic chromatin restructuring and reprogramming events that occur during sperm formation and in early embryo development (Table 2, Figure 7).

**Table 2:** Summary of the profiles of histone post-translational modifications during spermatogenesis and after fertilization.

Subtype	*C. elegans* Residue	Mouse Residue	PTM	Both Sexes		Male						Herm - 1 oocyte	Embryos				
				mitotic/TZ	pachytene	pachytene X	karyosome	diakinesis	meta 1	meta 2	post-meiosis		early 1 cell		late 1 cell	2 cell	>8 cell
													M	F			
**Removed from paternal during spermatogenesis and after fertilization**																	
**H2A**	**K120**	**K119**	**ub**	++*	++*	++*	off-chromatin foci*	off-chromatin foci*	−	−	−	−/+	off-chromatin foci	−/+	−	−	++
**Removed from paternal during spermatogenesis**																	
**H2A**	**K5, K9, K14, K16**	**K5, K9, K13, K15**	**ac**	+++	+++	−	+++	+++	−	−	−	+++	−	+++	+	++	+++
**H3**	**K27**	**K27**	**ac**	+++	+++	−	++	+	−	−	−	+++	−	+++	+	+++	+++
**H4**	**K5**	**K5**	**ac**	−	++	−	++	−	−	−	−	+++	−	+++	+	+++	+++
**H4**	**K12**	**K12**	**ac**	+++	+++	−	+++	+	−	−	−	+++	−	+++	++	+++	+++
**H4**	**K16**	**K16**	**ac**	+++	+++	+	+++	++	+	−	−	+++	−	+++	+	++	+++
**H3**	**K79**	**K79**	**me3**	+++	+++	+++	++	−	−	−	−	+++	−	+++	−	−	++
**Removed from both paternal and maternal**																	
**H2B**	**K7**	**K12**	**ac**	+++	+++	−	+++	+	−	−	−	−	−	−	−	++	+++
**Retained on both paternal and maternal**																	
**H3**	**K23**	**K23**	**ac**	+++	+++	+++	+++	+++	+	+	−	+++	+	+++	+	+++	+++
**H4**	**K8**	**K8**	**ac**	+++	+++	+++	+++	+++	+++	++	−	+++	+	+++	+++	+++	+++
**H3**	**K36**	**K36**	**me1**	+++	+++	−	+++	+++	+++	++	small foci	+++	+++ (not X)	+++	++ (not paternal X)	+	+++
**H3**	**K79**	**K79**	**me2**	+++	+++	+	+++	++	++	++	−	++	++	+++	−/+	++	+++
**H4**	**K20**	**K20**	**me1**	+++	small foci +	+	small foci	small foci	small foci	small foci	small foci	small foci	small foci	small foci	+++	+++	+++

+++ high levels, ++ moderate levels, + low levels, − not detectable. The H2Aub profile reflects the staining patterns from E6C5, ABE569, and #308 antibodies except where denoted by (*) which is from E6C5 antibody only.

The sperm epigenetic profile is established by four partially overlapping stages during sperm formation in *C. elegans* (Figure 7A, B) that are then processed in the embryo. The first involves H2A ubiquitination. H2A ubiquitination occurs in *Drosophila*, mouse, and rat spermatogenesis [21], [23], [24]; however, the role of this modification remains unclear because reports concerning how loss of the ubiquitin conjugating enzyme, RNF8, affects histone replacement during spermiogenesis vary in mouse [23], [25]. A recent report has instead shown histones may be targeted to sperm-specific proteasomes via H4K16 acetylation [18]. In our studies, chromatin-associated H4K16ac and H2Aub are removed as chromosomes condense for meiotic divisions. Additionally, we found evidence of H2Aub off-chromatin foci sharing partial overlap with Ub-K48 conjugates, which target proteins to proteasomes, and Ub-K63 conjugates, which are linked to the DNA damage response and autophagy (Figure S4), though only one (E6C5) of four antibodies we tested showed a clear signal in the male germ line [77]. However, the dynamics of ubiquitin conjugates and the proteasome localization defined here for the first time in the *C. elegans* germ line suggests that Ub-K48 and Ub-K63 pathways are active during late spermatogenesis when chromatin is remodeled (Figure S4). Furthermore, after fertilization, the appearance of H2Aub detected by three antibodies (E6C5, ABE569, and #308) as paternal chromatin decondenses suggests paternally contributed histones are targeted for destruction, just as other sperm-specific proteins, like membranous organelles, are targeted for autophagy [76], [85]. Thus, ubiquitination may play a general role in the immediate clearance of sperm proteins in the embryo after fertilization.

Incorporation of the sperm-specific H2A variant HTAS-1 is the next phase of chromatin remodeling (Figure 7A, B). Incorporation of histone variants before completion of meiosis occurs in other organisms (Figure 7C). For example, TH2B facilitates protamine incorporation in mouse and rat [9], [86]. Other histone variants, such as H2AL1/2 in mouse, are retained at pericentric regions, then delivered to the embryo and removed upon sperm decondensation [14], [16]. In contrast, HTAS-1 is carried over and not erased in the 1-cell embryo. This raises the intriguing possibility that HTAS-1 may be specifically retained at specific genomic sites for function in the embryo. In contrast, HTZ-1 is removed from paternal and maternal chromatin rapidly in the new embryo. Though it is not clear if HTZ-1 removal coincides with DNA replication, studies in mice show that maternally-contributed H2A.Z removal is DNA replication-independent [87]. Thus, it remains to be determined which structural differences between H2A variants may alter the stability of nucleosomes during DNA replication or are recognized by histone chaperones or chromatin modifiers [88], [89].

The third stage of chromatin remodeling during sperm formation is the bulk loss of many histone PTMs that results in the lack of histone acetylation marks on paternal chromatin in the embryo (Figure 5, Table 2). Levels of all histone PTMs fall as sperm chromosomes condense and divide. However, we found that removal prior to the second meiotic division correlates with erasure, indicating this cell cycle transition may be a critical juncture that coordinates histone PTM removal (Figure 7A, Table 2). After fertilization, many maternal and paternal chromatin histone PTM marks subsequently become equalized. Work from this study and others show this occurs via distinct mechanisms [5], [33], [84]. A notable mark that is not equalized is H4K16ac, which marks oocyte chromatin but remains absent from paternal chromatin. However, by the 4-cell stage, levels of all histone PTMs progressively rebound (Figure 7A). While embryonic transcription may be a factor, several marks are established on chromatin of the transcriptionally-silenced germline precursor cell (Figure S7). Thus the regulatory factors that temporally regulate histone modifications during the oocyte-to-embryo transition remain to be elucidated.

Histone PTMs that persist into the second meiotic division during spermatogenesis are retained (Figure 7A, Table 2). For example H4K20me1 forms small chromatin-associated foci detectable before and after fertilization. In later embryos, H4K20me1 levels rebound on all chromosomes then later become enriched on X chromosomes to function in dosage compensation [83], [90]. Further investigation will reveal whether H4K20me1 dynamics play roles in sperm or early embryos, as H4K20 methylation has a broad range of functions, including DNA damage response, DNA replication, mitotic condensation, and transcription [91]. Overall, we have found that many of the PTM marks in *C. elegans* share similar fates as those in mouse (Table S8). Importantly, our proteomic analysis has also identified both histone methylation and acetylation marks retained in sperm chromatin that also have the potential to pass on specific paternal epigenetic information to the embryo (Table 1, Table 2, Table S8).

The last stage of restructuring is the incorporation of sperm nuclear basic proteins (SNBPs), which exhibits differences compared with mammals (Figure 7B, C). Most strikingly, in *C. elegans*, incorporation of HTAS-1 and the putative protamines SPCH-1, 2, 3 overlaps with meiotic divisions (Figure 2, Figure 7A, B) [35], [55], in contrast to mammalian transition proteins and protamines that are incorporated after meiosis (Figure 7C) [92]. Furthermore, *C. elegans* sperm retain more histones compared to mammals. This may be due to the rapid progression of spermatogenesis as well as a lack of identifiable *C. elegans* transition proteins. Future studies, including assessing *spch* gene loss-of-function, are necessary to support the possibility that SPCH-1, 2, and 3 function as *C. elegans* protamines and to determine whether each plays a distinct role in sperm formation.

The overlap of events also results in a lack of a post-meiotic burst of transcription in *C. elegans* spermatogenesis, which prepares gametes for chromosome restructuring and spermiogenesis in mammals [4] (Figure 7B, C). In *C. elegans*, transcription is globally repressed before meiotic divisions and not reactivated after meiosis [55], [56], [93]. Further, *C. elegans* does not exhibit post-meiotic histone hyperacetylation, which plays roles in transcriptional activation and histone displacement in mammals and *Drosophila* [18]–[20], [94]. In *C. elegans*, histone acetylation levels remain high on nuclei until meiotic divisions, at which point levels rapidly drop (Figure 7B and Figure S6) [56]. The overlap, omission, or abbreviation of these events likely contributes to the rapid progression of sperm formation observed in *C. elegans* (∼24 hours) compared to mouse (∼30 days) or humans (∼60 days) [56], [95]–[97].

Historically, identifying epigenetic marks in sperm and embryos solely using cytology or western analysis in any organism has been challenging. Antibody availability is limited and specificity in different applications is variable [53]. For example, in this work, we tested four distinct antibodies used in previous studies to detect H2A ubiquitination: the monoclonal E6C5 and the highly purified polyclonal ABE569, #87, and #308 antibodies [54], [72]. Antibody #87 exhibited specificity in western analysis (Figure 1F), while E6C5, ABE569 and #308 showed distinct staining patterns in immunostaining experiments (Figure 4, Figure S4, Figure S5). While one possibility is that they may recognize off-target proteins or unmodified H2A in immunostaining experiments, it is also possible that they are specific for distinct epitopes that are accessible under specific conditions (i.e. denatured or partially folded protein conformations) because each was raised in individual animals against antigens with multiple possible epitopes. Another issue is whether lack of staining indicates the absence of a protein or the inaccessibility of antibodies to the tightly compacted paternal chromatin. Indeed, we observed the interior of the *C. elegans* spermatid nucleus is resistant to antibody staining regardless of the antibody or fixation condition used. Thus, the sole use of antibodies to identify histone PTMs should be used with caution [98].

Overall our study shows that despite the limitations of proteomics, western analysis and cytology, their combined use was an effective strategy to mine for modification sites and then define the spatial and temporal dynamics of their addition and removal. Further high-resolution studies of gametes and early embryos are necessary to distinguish specific genes associated with the epigenetic markers identified here. However, our studies provide key landmarks for understanding the dynamics of epigenetic erasure and reprogramming required for both sperm cell and early embryonic development.

## Methods

### Strains

*C. elegans* strains were maintained using standard conditions [99]. Strains used in this study are CB1489 *him-8(e1489)*, DR466 *him-5(e1490)*, TY0119 *fem-1(hc17ts)* and JK0816 *fem-3(q20ts)*. Strains were cultured at 20°C, except for TY0119 *fem-1(hc17ts)* and JK0816 *fem-3(q20ts)*, which were maintained at 15°C.

### Isolation of *C. elegans* sperm and embryos

Large scale culturing of *C. elegans* hermaphrodites was conducted as in [35] except that *fem-3(q20ts)* animals were cultured at 15°C. Synchronous cultures of *fem-3(q20ts)* hermaphrodites were treated with a 1.2% sodium hypochlorite, 0.5 N NaOH solution with vigorous vortexing to release embryos. After washing in M9 buffer to remove hypochlorite, the embryos were either quick-frozen in liquid nitrogen and stored at −80°C degrees for chromatin isolation or were hatched overnight in M9 solution. The resulting L1 larvae were plated onto egg plates and cultured for 4 days at 25°C. Sperm was isolated as in [35] and frozen at −80°C degrees.

### Histone quantitation

Approximately 600 µL of mixed-stage N2 embryos and 600 µL of *fem-3(q20ts)* sperm were collected and lysed by 6 rounds of sonication (3 seconds at 10% amplitude) and rest (1 minute on ice). Genomic DNA was isolated using phenol/chloroform (Koelle lab) from 200 µL of N2 embryo lysate or 200 µL of *fem-3 (q20ts)* sperm lysate, resuspended in 100 µL of dH_2_O, and quantified with the Qubit dsDNA BR Assay Kit on a Qubit 2.0 Fluorometer (Life Technologies). Titrations of sperm and embryo lysates representing equivalent amounts of DNA were subjected to 4–20% SDS PAGE (Bio-Rad) or 14% SDS PAGE (Fisher Scientific) and western analysis. Primary antibodies (and the dilutions) used include: anti-HTAS-1 (at 1∶1000, polyclonal rabbit antibodies 3656 and 3657 generated against the last 15 amino acids of the C-terminal, LPKKKAKEDDKENNS), anti-SPCH (at 1∶500, rabbit 2391 [35]), anti-HTZ-1 (a polyclonal rabbit antibody from the lab of Dr. Gyorgyi Csankovszki used at 5 ug/mL for the westerns) and anti-histone PTMs shown in Table S7. HRP-conjugated donkey anti-rabbit (abcam ab6802) was used at a 1∶1000 dilution. HRP signal was detected using SuperSignal Chemiluminescent Substrate (Pierce). Western blots were analyzed using a Kodak Imager. The Gel Analyzer tool from ImageJ was used to quantify bands from western blot analysis.

### Isolation and acid solubilization of *C. elegans* spermatogenic and embryonic chromatin

For chromatin preparations, a minimum of 500 µL of frozen sperm or embryos was used per isolation. Cells were lysed as described above. Chromatin was isolated as in [35] except centrifugation through a sucrose gradient was conducted at 100,000×g for 1 hour at 4°C and 1 mM sodium orthovanadate (Na_3_VO_4_), 10 mM sodium butyrate (Na(C_3_H_7_COO)), and 0.02% protease inhibitors (Calbiochem) were added to all buffers prior to use. Nuclear basic proteins from sperm and embryo chromatin were isolated by resuspending samples in 0.4 N H_2_SO_4_ acid for 1 hour at 4°C [44]. Samples were subjected to centrifugation at 16,000×g for 10 minutes at 4°C. The resulting supernatant was resuspended in 500 µl of Buffer A [35].

### Multidimensional Protein Identification Technology (MudPIT) identification of sperm and embryo acid-soluble chromatin-associated proteins

#### Trichloroacetic acid (TCA) precipitation

Two spermatogenic chromatin and two embryonic acid-solubilized chromatin protein samples were each precipitated by mixing 1 volume of the cold sample solution with 1/3 volume of 100% (w/v) TCA (6.1 N, Sigma) to give a final TCA concentration of 25%. Samples were left on ice for 3 hours then centrifuged for 30 minutes at 4°C. The resulting pellets were washed twice with ice-cold acetone (500 µL each). After each wash, the solution was centrifuged for 10 minutes. Samples were dried on a Speed-vac for 1–2 minutes or air-dried.

#### Protease digestion

Precipitated chromatin proteins were dissolved in 60 µL of 100 mM Tris-HCl, pH 8.5 containing 8M urea (Sigma). The protein was reduced by incubation in 5 mM *tris*(2- carboxyethyl)phosphine (TCEP) for 20 minutes at room temperature followed with carboxyamidomethylation of cysteines by incubation at room temperature for 30 minutes in the dark in 10 mM iodoacetamide. The sample was diluted 2-fold (to 4M of the concentration of urea) by the addition of an equal volume of 100 mM Tris-HCl, pH 8.5 and then Lys-C (Promega) was added at ∼1∶100 enzyme to substrate ratio (wt∶wt) and incubated at 37°C for 4 hours in the dark. The sample was then further diluted to the concentration of 2M urea and trypsin (Promega) was added at ∼1∶100 enzyme to substrate ratio (wt∶wt) and incubated at 37°C overnight in the dark. The resulting peptides from the digests were dissolved using 90% formic acid to a final concentration of 2% formic acid. The sample was stored at −20°C prior to LC/LC-MS/MS analysis.

#### Multidimensional Protein Identification Technology (MudPIT)

Total peptide mixtures were pressure-loaded onto a biphasic fused-silica capillary column (250 µm i.d.) consisting of 2.5 cm long strong cation exchange (5 µm Partisphere SCX, Whatman, Clifton, NJ) and 2.5 cm long reversed phase (Aqua C18, Phenomenex, Torrance, CA) that was prepared by slurry packing using an in-house high pressure vessel. The column was washed with buffer A [water/acetonitrile/formic acid (95∶5∶0.1, v/v/v)] for more than 10 column volumes (100 µm i.d.) with a through-hole union (Upchurch Scientific, Oak Harbor, WA). The analytical columns were pulled beforehand by a laser puller (Sutter Instrument Co., Novato, CA) with a 5 µm opening. It was packed with 3 µm reversed phase (Aqua C18, Phenomenex, Torrance, CA) to 15 cm long. The entire column setting (biphasic column-union-analytical column) was placed in-line with an Agilent 1200 quaternary HPLC pump (Palo Alto, CA) for mass spectrometry analysis. The digested proteins were analyzed using a 12-step MudPIT separation method as described previously [100], [101]. The elution gradient of step 1 was as follows: 10 min of 100% buffer A, a 5 min gradient from 0 to 15% buffer B [water/acetonitrile/formic acid (20∶80∶0.1, v/v/v)], a 65 min gradient from 15 to 45% buffer B, a 15 min gradient from 45 to 100% buffer B, and 5 min of 100% buffer B. Steps 2–11 had the following profile: 1 min of 100% buffer A, 4 min of X% buffer C (500 mM ammonium acetate with 5% acetonitrile and 0.1% formic acid), followed by the same gradient as step 1. The 4 min buffer C percentages (X) were 10, 20, 30, 40, 50, 60, 70, 80, 90, and 100%, respectively. The salt pulse for the final step (step 12) was 90% buffer C plus 10% buffer B (450 mM ammonium acetate in 12.5% acetonitrile).

#### Mass spectrometry conditions

Data-dependent tandem mass spectrometry (MS/MS) analysis was performed with an LTQ-Orbitrap mass spectrometer (ThermoFisher, San Jose, CA). Peptides eluted from the LC column were directly electrosprayed into the mass spectrometer with the application of a distal 2.5 kV spray voltage. A cycle of one full-scan MS spectrum (m/z 300–1800) was acquired followed by eight MS/MS events, sequentially generated on the first to the eighth most intense ions selected from the full MS spectrum at a 35% normalized collision energy. The number of microscans was one for both MS and MS/MS scans and the maximum ion injection time was 50 ms and 100 ms respectively. The dynamic exclusion settings used were as follows: repeat count, 1; repeat duration, 30 seconds; exclusion list size, 100; and exclusion duration, 180 seconds. MS scan functions and HPLC solvent gradients were controlled by the Xcalibur data system (ThermoFisher).

#### Data analysis

Full MS and tandem mass spectra were extracted from raw files, and the tandem mass spectra were searched against a Wormpep database (Ver. 04-25-12) [102]. In order to accurately estimate peptide probabilities and false discovery rates, we used a decoy database containing the reversed sequences of all the proteins appended to the original database. Tandem mass spectra were matched to sequences using the Sequest [47] or ProLuCID algorithm. Sequest and ProLuCID searches were done on an Intel Xeon 80-processor cluster running under the Linux operating system. The peptide mass search tolerance was set to 3 Da for spectra acquired on the LTQ instrument. The mass of the amino acid cysteine was statically modified by +57.02146 Da, to take into account the carboxyamidomethylation of the sample. Serine, threonine, and tyrosine were treated as differentially modified by +79.9663 Da for phosphorylation. Lysine and arginine were treated as differentially modified by +14.0157 Da for methylation, +28.0314 Da for di-methylation, and +42.0471 for tri-methylation. Lysine was treated as differentially modified by +42.0106 Da for acetylation, and +114.042927 Da for ubiquitination. No enzymatic cleavage conditions were imposed on the database search so the search space included all candidate peptides whose theoretical mass fell within the mass tolerance window, regardless of their tryptic status [43].

The validity of peptide/spectrum matches (PSMs) was assessed in DTASelect [45], [46] using two SEQUEST [47] defined parameters, the cross-correlation score (XCorr) and the normalized difference in cross-correlation scores (DeltaCN). The search results were grouped by charge state (+1, +2, +3, and greater than +3) and tryptic status (fully tryptic, half-tryptic, and non-tryptic) [103], resulting in 12 distinct sub-groups. In each one of these sub-groups, the distribution of Xcorr, DeltaCN, and DeltaMass values for (a) direct and (b) decoy database PSMs was obtained and then the direct and decoy subsets were separated by discriminant analysis. Full separation of the direct and decoy PSM subsets is not generally possible; therefore, peptide match probabilities were calculated based on a nonparametric fit of the direct and decoy score distributions. A peptide probability of 90% was set as the minimum threshold. The false discovery rate was calculated as the percentage of reverse decoy PSMs among all the PSMs that passed the 90% probability threshold. After this last filtering step, we estimate that both the protein and peptide false discovery rates were reduced to between 0.0% and 0.5%. PSMs with an absolute DeltaMass value of greater than 6 ppm were not accepted during filtering. Proteins identified are shown in Tables S1 and S2 and sorted by Total Spectral Counts. NSAF and EMPAI abundance values calculated from all peptides (including semi-tryptic fragments) identified are also presented and show histones are among the most abundant proteins in each sample [48]–[50]. Peptide match parameters for full tryptic peptides that match to histone proteins are shown in Tables S3–S6. NSAF and EMPAI abundance values are those calculated for all peptides as shown in Tables S2 and S3. Peptides that matched to multiple proteins were distributed as in [50]. Tables S3–S6 show all post-translational modifications identified. Annotated mass spectra for post-translationally modified peptides are shown in Files S1–S4. Table 1 shows the number of times (occurrences) a modification site was identified from peptides that bore both single and multiple PTMs. Thus, from 253 PTM histone peptides detected in acid-solubilized embryo chromatin, 330 modifications were identified. From 127 PTM histone peptides detected in acid-solubilized sperm chromatin, 135 modifications were identified (Tables S3–S6).

We searched for ubiquitination sites, however, because iodoacetamide was used during sample preparation, sites identified may be due to iodoacetamide-induced adducts [104]. Thus, we excluded these in our counts of identified PTMs. However, we note a possible enrichment of H2AK119ub (see *C. elegans* amino acid numbering, File S5) in embryo and not sperm chromatin samples. This could be valid because: (1) H2A ubiquitinated on lysine 119 (H2AK119ub) represents 132 spectral IDs in acid-solubilized embryo chromatin samples, compared to 0 spectral IDs in acid-solubilized sperm chromatin samples. This enrichment is unlikely due to non-specific adducts, which should be induced similarly in both samples, (2) H2AK119ub is the most abundant ubiquitination site in embryo chromatin samples, with the next most abundant ubiquitinated protein being PAR2, which is represented by only 11 spectral IDs. (3) The enrichment of H2AK119ub is also considerably higher compared to other modification sites identified on all histone proteins, which argues that H2AK119ub identification is not merely due the fact that histones are a major component of the sample. Ubiquitination of H2AK120 (which corresponds to mammalian K119) was also identified as shown in Table S3, though at lower levels. We have listed all histone ubiquitinated peptides identified (in gray text) in the total histone peptide lists (Tables S3–S6) but excluded them in the PTM counts (Table 1).

### Western analysis

TCA precipitated sperm and embryo acid-solubilized chromatin proteins representing one-fifth of that analyzed by MudPIT were resuspended in 50 µL of 1×LSM. 5 µL were subjected to SDS PAGE and western blotting as described above. Primary antibodies and the dilutions used for westerns are described above (HTAS-1, HTZ-1, SPCH) or shown in Table S7. Mouse anti-MSP (from the lab of Dr. David Greenstein) was used at 1∶1000 dilution [105].

### Immunostaining

Because sperm DNA is tightly compacted, we tested different fixation methods to achieve consistent and sensitive detection of proteins associated with spermatid chromatin. A methanol/acetone fixation (as detailed below) was the most effective for detecting integral chromatin proteins, such as histones, in comparison to methanol or ethanol/paraformaldehyde methods [35], [56], [106]. Using this method we detect staining on early spermatid chromatin for histone H1 (Figure S2), a protein previously shown to be present in sperm chromatin [64]. For most proteins tested, staining levels on sperm chromatin decrease gradually after completion of meiosis, suggesting that chromatin becomes progressively more compacted [56]. Each staining experiment was conducted a minimum of four times with at least three slides (each with 30–50 animals) for every experiment.

For methanol/acetone fixation, 30–60 adult *him-8(e1489)* males or hermaphrodites were dissected into sperm salts (50 mM Pipes, pH 7, 25 mM KCl, 1 mM MgSO_4_, 45 mM NaCl, and 2 mM CaCl_2_) on a slide. The slide was then freeze-cracked. For antibodies that recognize histones, slides were placed into 100% methanol for 10 minutes, then 100% acetone for 5 minutes and briefly air-dried. Slides were then washed 3 times with PBST (1× PBS, 0.5% Triton X-100, and 1 mM EDTA, pH 8) for 10 minutes each wash. For antibodies that recognize ubiquitin, MOs, and the proteasome, a methanol fixation procedure was also used, in which slides were placed in methanol for 30 minutes then processed as described. Primary and secondary antibodies were incubated overnight. Slides were washed with PBST as above then stained with DAPI (1 µg/ml) and mounted with Vectashield. Confocal images were obtained using a Zeiss LSM710 confocal microscope. Primary antibodies against commercially available post-translationally modified histones used in this study are listed in Table S7.

## Supporting Information

Figure S1 Isolation of histones from **A**) *C. elegans* sperm and **B**) embryos. Arrowheads mark histone H3, H2A and H2B (which migrate together), and H4. The band representing major sperm protein (MSP) is denoted by the asterisk. **C**) Western analysis of whole body extracts show that HTAS-1 is present only in sperm-producing animals [*fem-3(gf)*] and not oocyte-producing animals [*fem-1(lf)*], similar to MSP. HTZ-1 is present in both.
(TIF)

Figure S2 HTAS-1 and HTZ-1 incorporation into sperm chromatin. Immunolocalization of isolated and methanol/acetone fixed *C. elegans* male gonads. Scale bar represents 20 µm. Contrast levels were set to enhance visualization of staining on post-meiotic spermatids, thus earlier stages are over-saturated. Histone H1 (red) and DAPI-stained DNA (blue) costained with **A**) HTAS-1 (green) is visible on all post-meiotic nuclei. **B**) HTZ-1 (green) is visible on early post-meiotic nuclei but not on more proximal spermatid nuclei. Similarly, histone H1, which has previously been shown to be retained in sperm chromatin and passed over to the embryo, is also not visible in later post-meiotic spermatids [64].
(TIF)

Figure S3 Histone PTM marks in the new embryo. Immunolocalization of 1-cell embryos. Histone H1 (red) staining, which overlaps with DAPI staining for DNA (blue), is used as a control to show that lack of staining by co-markers is not due to antibody inaccessibility. The scale bar represents 5 µm and applies to all panels. **A**) After fertilization, HTZ-1 (green) levels are high on both maternal (m) and paternal (p) chromatin as oocyte chromosomes complete meiosis, showing that HTZ-1 is passed over by both gamete types. HTZ-1 levels are very low in 2-cell embryos indicating it has been removed. **B**) HTAS-1 (green) is present on paternal but not maternal chromatin in 1-cell embryos. **C**) H4K16ac (green), **D**) H4K12ac (green), and **E**) H2Apan-ac (green) are present on maternal but not paternal chromatin after fertilization.
(TIF)

Figure S4 **A**) Chromatin-associated histone H2A ubiquitination (H2Aub) decreases as HTAS-1 is incorporated. Male germ lines were dissected, fixed, and costained with the DNA dye DAPI (blue in merged image). **A**) The monoclonal E6C5 antibody that recognizes H2Aub (green in merged image) [73] and HTAS-1 (red in merged image). Arrows mark examples of early (yellow) and late (white) off-chromatin foci. The scale bar represents 50 µm. **B–E**) During spermatogenesis, the H2Aub localization pattern using E6C5 is distinct from that of poly-ubiquitin conjugates. H2Aub (green) and (in red): **B**) K48-linkage specific polyubiquitin (Ub-K48) that targets proteins for degradation via the **C**) proteasome; **D**) K63-linkage specific polyubiquitin (Ub-K63); or **E**) Membranous Organelles (MOs). The regions in the white dotted boxes represent 20 µM and are shown enlarged in the panels below each section. These show that though some H2Aub off-chromatin foci overlap with Ub-K48 and Ub-K63 polyubiquitin conjugates, some do not. In panel D the region of nuclei positive for H2Aub foci (denoted with a green line) begins and ends earlier than the region of nuclei positive for Ub-K63 staining (marked with a red line). H2Aub staining does not overlap with MO staining during spermatogenesis.
(TIF)

Figure S5 H2Aub dynamics after fertilization. Immunostaining of dissected and fixed hermaphrodite gonads with antibodies specific to H2Aub (green). **A**) E6C5 monoclonal antibody [73] (green) or **B**) #308 polyclonal antibody [54] (green) and DAPI-stained DNA (red). Polar bodies are denoted by ‘pb’, ‘m’ is maternal, ‘p’ is paternal. Scale bars represent 10 µm for all panels. Both antibodies show that H2Aub levels on chromatin decrease in maturing oocytes closest to the spermatheca (−1 and −2) [65]. E6C5 exhibit an increase in off-chromatin foci (white arrowheads). Though H2Aub is absent on sperm chromatin before fertilization, it is present at high levels on and off paternal chromatin after fertilization in the oocyte meiotic embryo. Chromatin-associated levels of H2Aub remain low in 1- and 2-cell embryos but rise to high levels in embryos with greater than 16 cells. Higher magnification images of H2Aub staining around paternal DNA is shown in Figure 4.
(TIF)

Figure S6 Histone acetylation levels decrease during late sperm formation. Immunostaining of dissected and fixed male gonads with antibodies specific to acetylated histones (green) and DNA stained with DAPI (red). **A–E**) are modifications under-represented on the X chromosome (circled with white dotted line and labeled “X”). **A**) H4K16ac levels are high until metaphase 1 then not detectable on metaphase 2 nuclei. **B**) H3K27ac levels are high until diakinesis then drop during meiotic divisions. **C**) H4K5ac is unevenly distributed on chromosomes and levels on DNA drop before meiotic divisions. **D**) H4K12Ac levels are not detectable during meiotic divisions. **E**) H2Apan-ac levels are high until diakinesis then not detected during meiotic divisions. **F**) H2BK12ac (corresponding to H2BK7 in *C. elegans*, see File S5) is not detectable above background levels during sperm meiotic divisions. **G–H**) are modifications present on autosomes and the X chromosome. **G**) H4K8ac and **H**) H3K23ac levels fall during late sperm formation but are still visible on metaphase II chromosomes.
(TIF)

Figure S7 Establishment of the histone modification marks H4K16ac, H2BK12ac (corresponding to H2BK7 in *C. elegans*, see File S5), and H3K79me2 do not rely on transcriptional activation in the new embryo. 4 to 8-cell embryos fixed and costained with a P-granule marker (red) that marks the P_2_ germ cell precursor cell, whose DNA remains transcriptionally silenced even as embryonic transcription begins in other cells. Histone modification marks (green) **A**) acetylation of H4 on lysine 16 (H4K16ac), **B**) acetylation of H2B on lysine 12 (H2BK12ac), and **C**) acetylation of H3 on lysine 79 (H3K79me2) are detected on the transcriptionally-silenced P_2_ cell nuclei. The scale bar represents 10 µm and applies to all panels.
(TIF)

Figure S8 Histone mono-methylation at H3K36, H4K20, and di-methylation at H3K79 are retained in sperm chromatin. Immunostaining of dissected and fixed male gonads with antibodies specific to methylated histones (green) and DNA stained with DAPI (red). The position of the X chromosome (X), which was determined by H3K9me2 co-staining (not shown), is circled. **A**) H3K36me1 levels remain high through sperm meiotic divisions with low levels of staining on spermatid DNA. **B**) H4K20me1 levels on chromatin fall prior to the diplotene stage. Bright foci of staining not associated with the X chromosome are visible in nuclei from the pachytene stage through spermatids. **C**) H3K79me2 levels are high through sperm meiotic divisions but not detectable on spermatid DNA. **D**) Levels of H3K79me3 become undetectable on chromosomes after the karyosome stage.
(TIF)

File S1 Annotated mass spectra that identify post-translationally modified histone H2A peptides. Each slide number corresponds to the Slide # shown in Table S3.
(PPTX)

File S2 Annotated mass spectra that identify post-translationally modified histone H2B peptides. Each slide number corresponds to the Slide # shown in Table S4.
(PPTX)

File S3 Annotated mass spectra that identify post-translationally modified histone H3 peptides. Each slide number corresponds to the Slide # shown in Table S5.
(PPTX)

File S4 Annotated mass spectra that identify post-translationally modified histone H4 peptides. Each slide number corresponds to the Slide # shown in Table S6.
(PPTX)

File S5 Amino acid alignments for *C. elegans* and mouse histone proteins. We follow the convention of numbering histone proteins starting at the amino acid after the starting methionine. For histone H2A, the red text indicates that K119 in mouse corresponds to K120 in *C. elegans*.
(PDF)

Table S1 List of proteins identified in acid-solubilized sperm chromatin by MuDPIT analysis. Locus is either the gene name or highly identical proteins that were not differentiated by peptide IDs (clusters). Unique IDs is the number of unique unmodified peptides identified that match to a specific protein. Total Spectral counts is the number of total unmodified peptides identified that match to a specific protein and include semi-tryptic peptides. Coverage is the % of the protein identified by peptides that match to a specific protein. NSAF and EMPAI are relative abundance values in which higher values correlate with higher abundance [48]–[50]. The table is sorted from highest to lowest Total Spectral Counts. Histone proteins are shown in blue text. SPCH proteins are shown in green text.
(XLSX)

Table S2 List of proteins identified in acid-solubilized embryo chromatin by MuDPIT analysis. Locus is either the gene name or highly identical proteins that were not differentiated by peptide IDs (cluster). Unique IDs is the number of unique unmodified peptides identified that match to a specific protein. Total Spectral counts is the number of total unmodified peptides identified that match to a specific protein and include semi-tryptic peptides. Coverage is the % of the protein identified by peptides that match to a specific protein. NSAF and EMPAI are relative abundance values in which higher values correlate with higher abundance [48]–[50]. The table is sorted from highest to lowest Total Spectral Counts. Histone proteins are shown in blue text.
(XLSX)

Table S3 Peptides from all histone H2A proteins that were identified by MuDPIT analysis. Full tryptic peptides matching to each histone H2A proteins (locus) are listed. Sequence count is the number of unique peptides identified that match to the proteins shown. Spectrum Count is the total number of full-tryptic peptides identified that match to each set of proteins shown. Peptides shown in grey text were not included in spectral counts. Sequence Coverage is the % of the protein that was detected by peptides identified. Peptide features identified by mass spectrometric analysis are shown. NSAF and EMPAI are relative abundance values in which higher values correlate with higher abundance [48]–[50]. Modified residues (*PTMs*) are denoted by adjacent parenthesis with the molecular weight of the modification shown: acetylation (42.0106), methylation (14.0157), di-methylation (28.0313), and ubiquitination (114.0429). Peptides shown in grey text were not included in spectral counts.
(XLSX)

Table S4 Peptides from all histone H2B proteins that were identified by MuDPIT analysis. Full tryptic peptides matching to histone H2B proteins are listed. Sequence count is the number of unique peptides identified that match to the proteins shown. Spectrum Count is the total number of full-tryptic peptides identified that match to each set of proteins shown. Peptides shown in grey text were not included in spectral counts. Sequence Coverage is the % of the protein that was detected by peptides identified. Peptide features identified by mass spectrometric analysis are shown. NSAF and EMPAI are relative abundance values in which higher values correlate with higher abundance [48]–[50]. Modified residues (*PTMs*) are denoted by adjacent parenthesis with the molecular weight of the modification shown: acetylation (42.0106), methylation (14.0157), di-methylation (28.0313), and ubiquitination (114.0429).
(XLSX)

Table S5 Peptides from all histone H3 proteins that were identified by MuDPIT analysis. Full tryptic peptides matching to each histone H3 protein (locus) are listed. Sequence count is the number of unique peptides identified that match to the proteins shown. Spectrum Count is the total number of full-tryptic peptides identified that match to each set of proteins shown. Peptides shown in grey text were not included in spectral counts. Sequence Coverage is the % of the protein that was detected by peptides identified. Peptide features identified by mass spectrometric analysis are shown. NSAF and EMPAI are relative abundance values in which higher values correlate with higher abundance [48]–[50]. Modified residues (*PTMs*) are denoted by adjacent parenthesis with the molecular weight of the modification shown: acetylation (42.0106), methylation (14.0157), di-methylation (28.0313), and ubiquitination (114.0429).
(XLSX)

Table S6 Peptides from all histone H4 proteins that were identified by MuDPIT analysis. Full tryptic peptides matching to each histone H4 protein (locus) are listed. Sequence count is the number of unique peptides identified that match to the proteins shown. Spectrum Count is the total number of full-tryptic peptides identified that match to each set of proteins shown. Peptides shown in grey text were not included in spectral counts. Sequence Coverage is the % of the protein that was detected by peptides identified. NSAF and EMPAI are relative abundance values in which higher values correlate with higher abundance [48]–[50]. Modified residues (*PTMs*) are denoted by adjacent parenthesis with the molecular weight of the modification shown: acetylation (42.0106), methylation (14.0157), di-methylation (28.0313), and ubiquitination (114.0429).
(XLSX)

Table S7 Antibodies used in this study that recognize post-translationally modified histone proteins. Vendor (or lab head), catalog or ID number, and dilution used for immunostaining or western analysis in this study are listed. Results from the modENCODE project [53] are also listed (Positive signals in *C. elegans* for antibodies are indicated in the following applications: C is chromatin immunoprecipitation and W is western analysis).
(XLSX)

Table S8 The status of paternal histone modification marks identified and tracked in the embryo in this study compared with mouse.
(DOCX)
