# Supplementary figures and images for: The Specification and Global Reprogramming of Histone Epigenetic Marks during Gamete Formation and Early Embryo Development in C. elegans
Source: PLoS Genet. 2014 Oct 9;10(10):e1004588. doi: 10.1371/journal.pgen.1004588 (PMC4191889; doi:10.1371/journal.pgen.1004588)

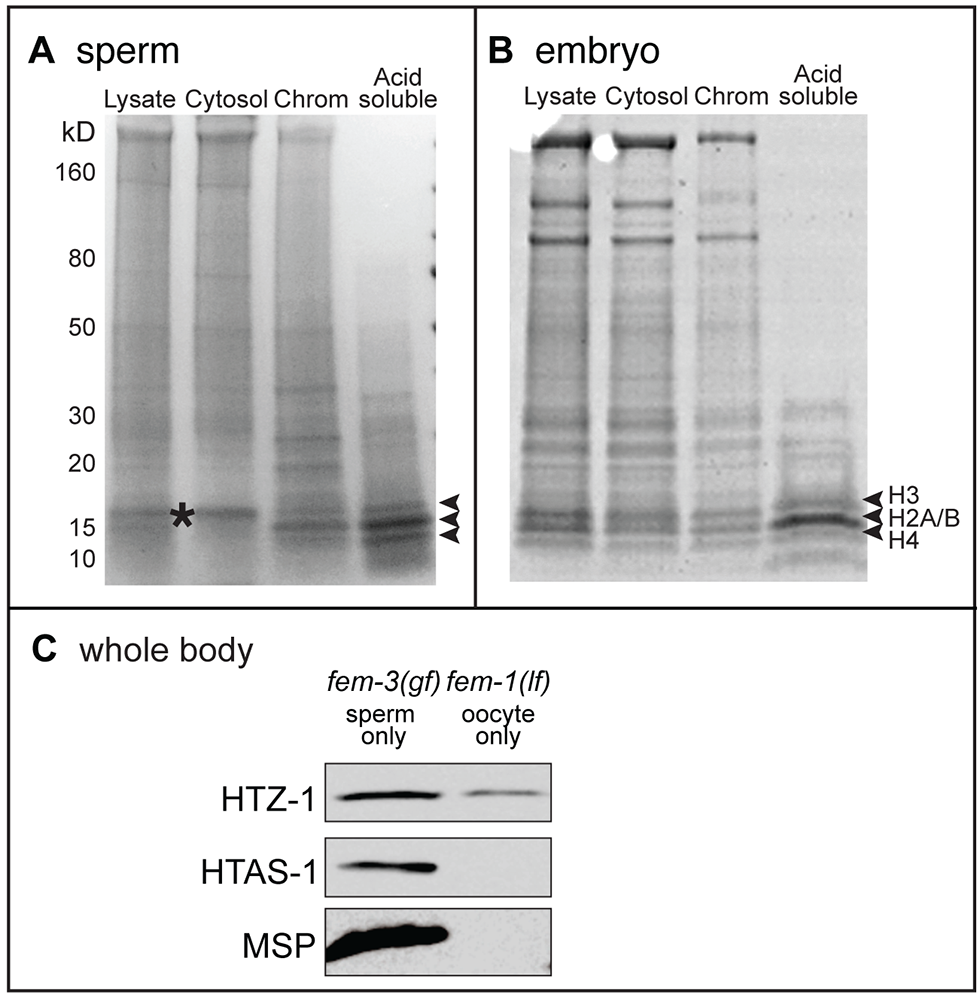

Supplement: Figure S1 — Isolation of histones from A) C. elegans sperm and B) embryos. Arrowheads mark histone H3, H2A and H2B (which migrate together), and H4. The band representing major sperm protein (MSP) is denoted by the asterisk. C) Western analysis of whole body extracts show that HTAS-1 is present only in sperm-producing animals [fem-3(gf)] and not oocyte-producing animals [fem-1(lf)], similar to MSP. HTZ-1 is present in both. (TIF) [file pgen.1004588.s001.tif]

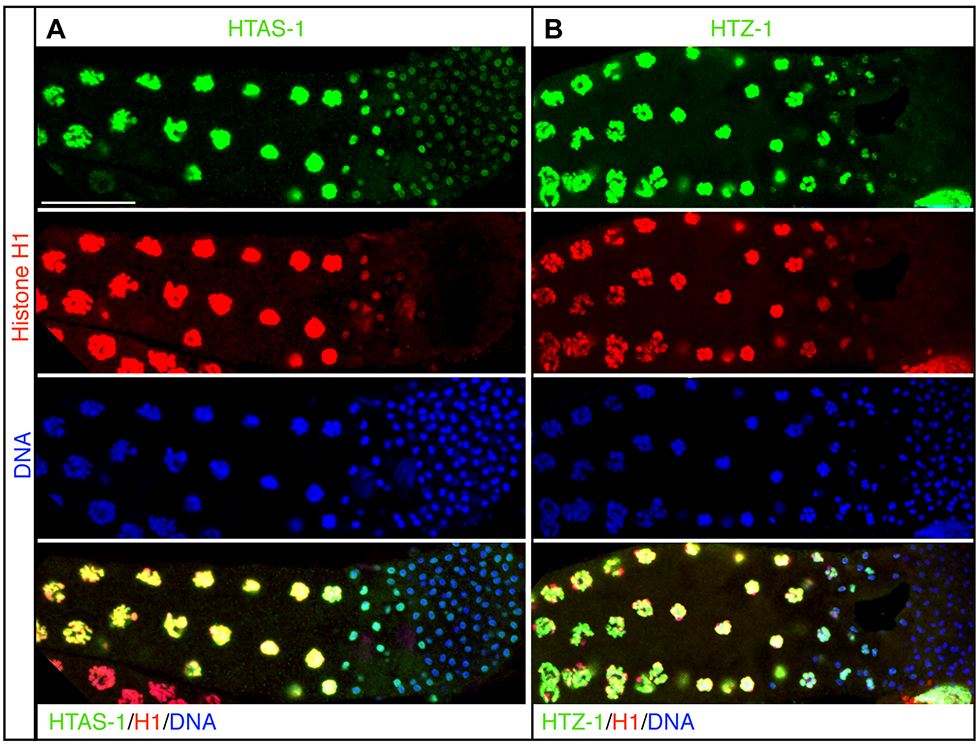

Supplement: Figure S2 — HTAS-1 and HTZ-1 incorporation into sperm chromatin. Immunolocalization of isolated and methanol/acetone fixed C. elegans male gonads. Scale bar represents 20 µm. Contrast levels were set to enhance visualization of staining on post-meiotic spermatids, thus earlier stages are over-saturated. Histone H1 (red) and DAPI-stained DNA (blue) costained with A) HTAS-1 (green) is visible on all post-meiotic nuclei. B) HTZ-1 (green) is visible on early post-meiotic nuclei but not on more proximal spermatid nuclei. Similarly, histone H1, which has previously been shown to be retained in sperm chromatin and passed over to the embryo, is also not visible in later post-meiotic spermatids [64]. (TIF) [file pgen.1004588.s002.tif]

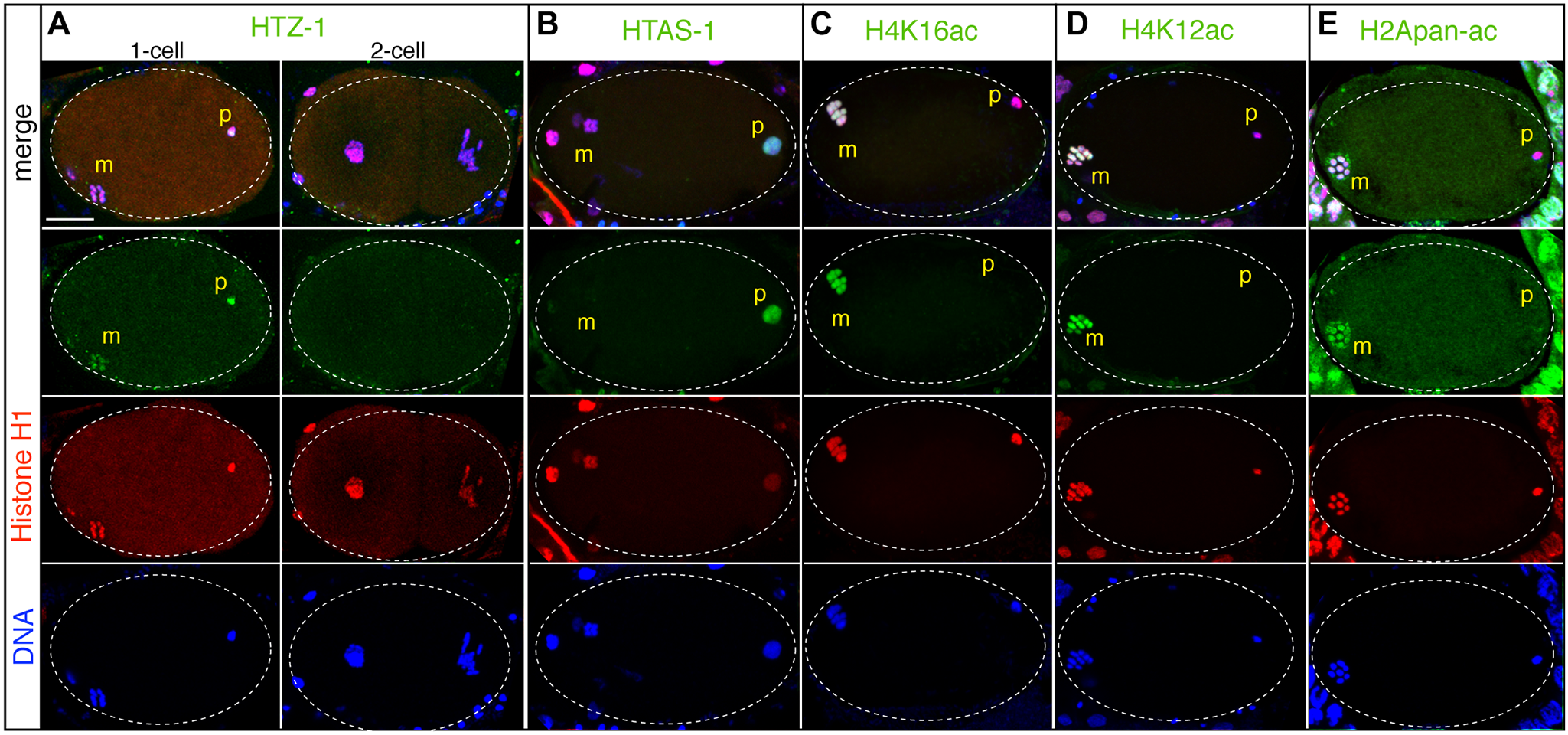

Supplement: Figure S3 — Histone PTM marks in the new embryo. Immunolocalization of 1-cell embryos. Histone H1 (red) staining, which overlaps with DAPI staining for DNA (blue), is used as a control to show that lack of staining by co-markers is not due to antibody inaccessibility. The scale bar represents 5 µm and applies to all panels. A) After fertilization, HTZ-1 (green) levels are high on both maternal (m) and paternal (p) chromatin as oocyte chromosomes complete meiosis, showing that HTZ-1 is passed over by both gamete types. HTZ-1 levels are very low in 2-cell embryos indicating it has been removed. B) HTAS-1 (green) is present on paternal but not maternal chromatin in 1-cell embryos. C) H4K16ac (green), D) H4K12ac (green), and E) H2Apan-ac (green) are present on maternal but not paternal chromatin after fertilization. (TIF) [file pgen.1004588.s003.tif]

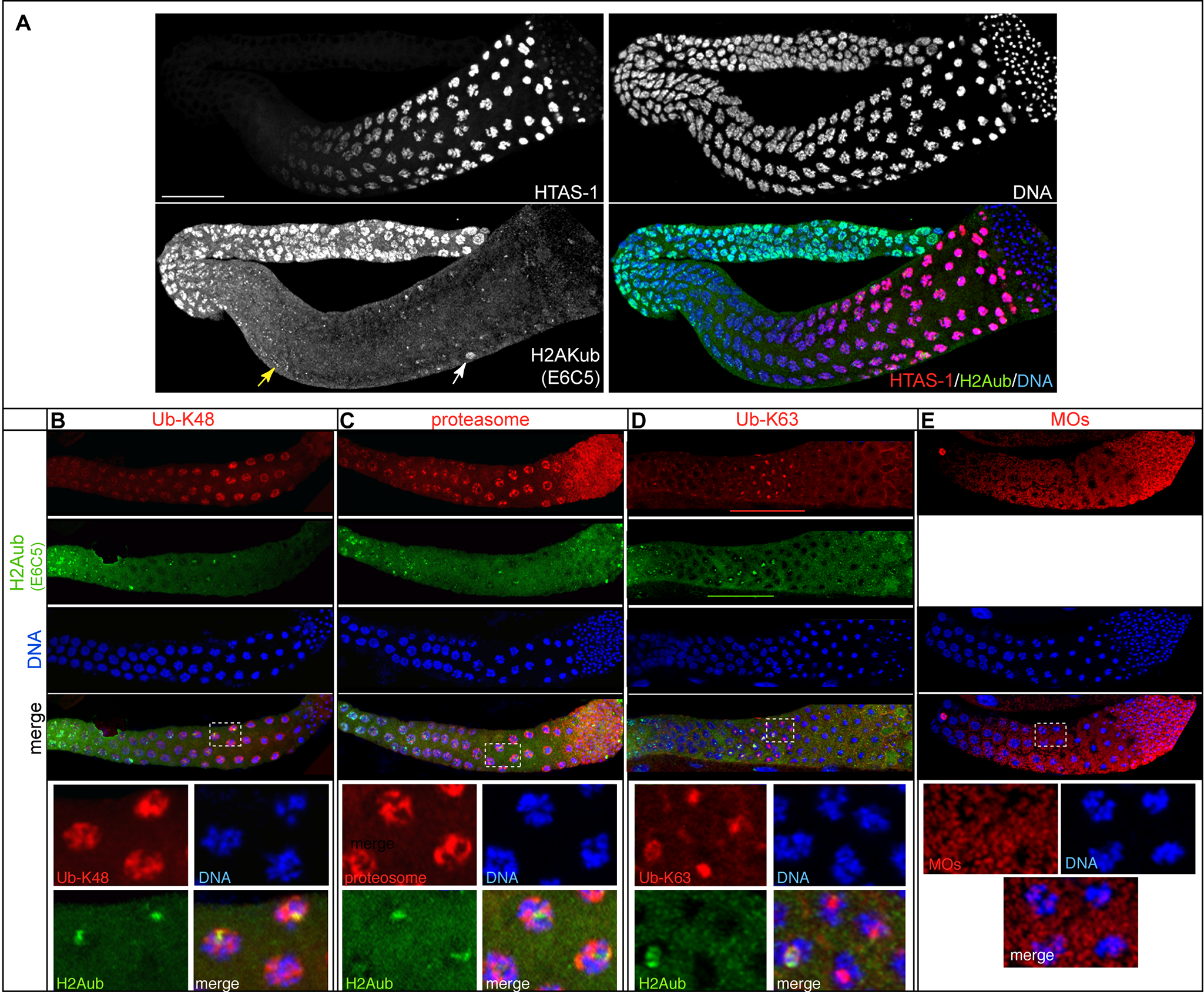

Supplement: Figure S4 — A) Chromatin-associated histone H2A ubiquitination (H2Aub) decreases as HTAS-1 is incorporated. Male germ lines were dissected, fixed, and costained with the DNA dye DAPI (blue in merged image). A) The monoclonal E6C5 antibody that recognizes H2Aub (green in merged image) [73] and HTAS-1 (red in merged image). Arrows mark examples of early (yellow) and late (white) off-chromatin foci. The scale bar represents 50 µm. B–E) During spermatogenesis, the H2Aub localization pattern using E6C5 is distinct from that of poly-ubiquitin conjugates. H2Aub (green) and (in red): B) K48-linkage specific polyubiquitin (Ub-K48) that targets proteins for degradation via the C) proteasome; D) K63-linkage specific polyubiquitin (Ub-K63); or E) Membranous Organelles (MOs). The regions in the white dotted boxes represent 20 µM and are shown enlarged in the panels below each section. These show that though some H2Aub off-chromatin foci overlap with Ub-K48 and Ub-K63 polyubiquitin conjugates, some do not. In panel D the region of nuclei positive for H2Aub foci (denoted with a green line) begins and ends earlier than the region of nuclei positive for Ub-K63 staining (marked with a red line). H2Aub staining does not overlap with MO staining during spermatogenesis. (TIF) [file pgen.1004588.s004.tif]

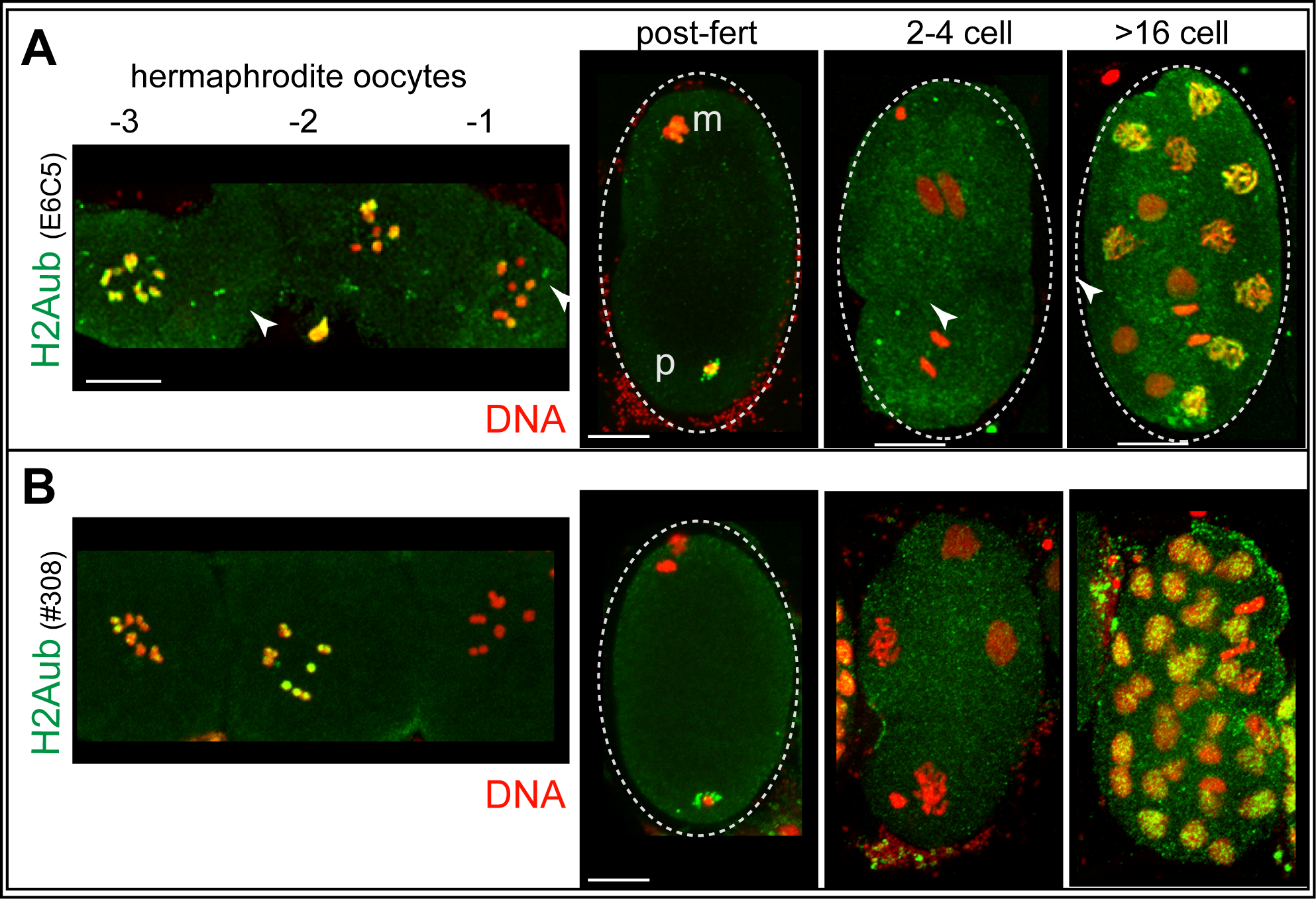

Supplement: Figure S5 — H2Aub dynamics after fertilization. Immunostaining of dissected and fixed hermaphrodite gonads with antibodies specific to H2Aub (green). A) E6C5 monoclonal antibody [73] (green) or B) #308 polyclonal antibody [54] (green) and DAPI-stained DNA (red). Polar bodies are denoted by ‘pb’, ‘m’ is maternal, ‘p’ is paternal. Scale bars represent 10 µm for all panels. Both antibodies show that H2Aub levels on chromatin decrease in maturing oocytes closest to the spermatheca (−1 and −2) [65]. E6C5 exhibit an increase in off-chromatin foci (white arrowheads). Though H2Aub is absent on sperm chromatin before fertilization, it is present at high levels on and off paternal chromatin after fertilization in the oocyte meiotic embryo. Chromatin-associated levels of H2Aub remain low in 1- and 2-cell embryos but rise to high levels in embryos with greater than 16 cells. Higher magnification images of H2Aub staining around paternal DNA is shown in Figure 4. (TIF) [file pgen.1004588.s005.tif]

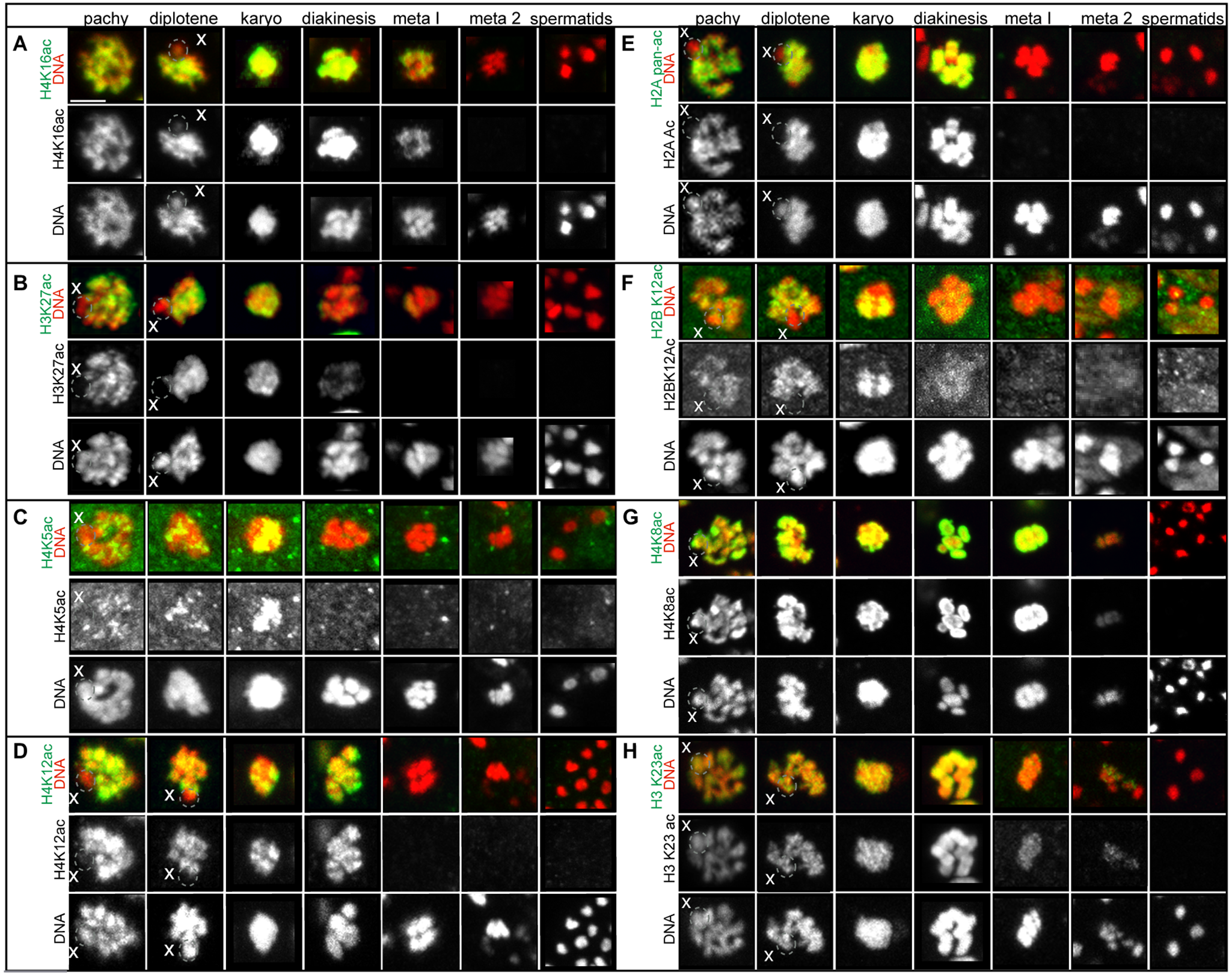

Supplement: Figure S6 — Histone acetylation levels decrease during late sperm formation. Immunostaining of dissected and fixed male gonads with antibodies specific to acetylated histones (green) and DNA stained with DAPI (red). A–E) are modifications under-represented on the X chromosome (circled with white dotted line and labeled “X”). A) H4K16ac levels are high until metaphase 1 then not detectable on metaphase 2 nuclei. B) H3K27ac levels are high until diakinesis then drop during meiotic divisions. C) H4K5ac is unevenly distributed on chromosomes and levels on DNA drop before meiotic divisions. D) H4K12Ac levels are not detectable during meiotic divisions. E) H2Apan-ac levels are high until diakinesis then not detected during meiotic divisions. F) H2BK12ac (corresponding to H2BK7 in C. elegans, see File S5) is not detectable above background levels during sperm meiotic divisions. G–H) are modifications present on autosomes and the X chromosome. G) H4K8ac and H) H3K23ac levels fall during late sperm formation but are still visible on metaphase II chromosomes. (TIF) [file pgen.1004588.s006.tif]

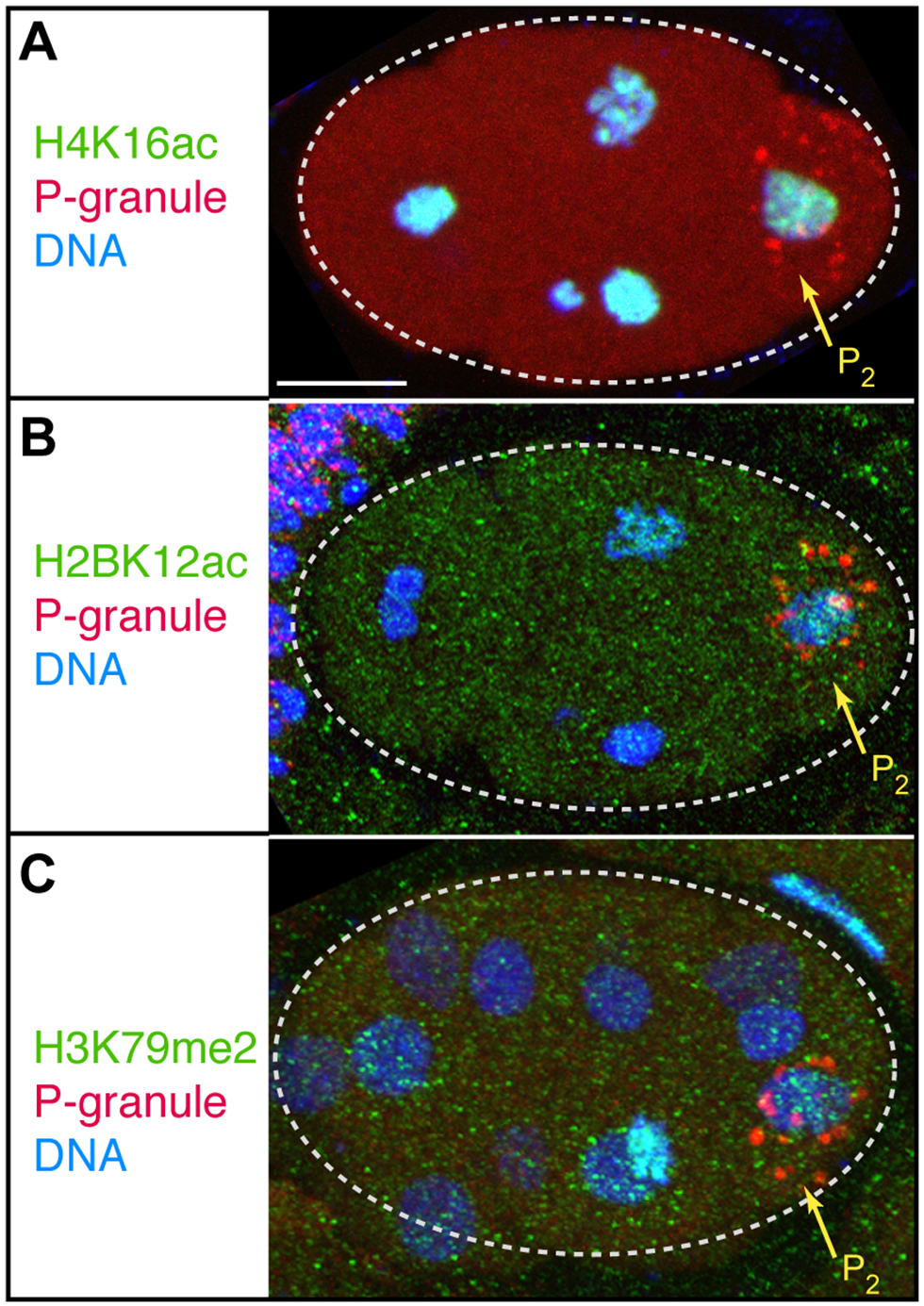

Supplement: Figure S7 — Establishment of the histone modification marks H4K16ac, H2BK12ac (corresponding to H2BK7 in C. elegans, see File S5), and H3K79me2 do not rely on transcriptional activation in the new embryo. 4 to 8-cell embryos fixed and costained with a P-granule marker (red) that marks the P2 germ cell precursor cell, whose DNA remains transcriptionally silenced even as embryonic transcription begins in other cells. Histone modification marks (green) A) acetylation of H4 on lysine 16 (H4K16ac), B) acetylation of H2B on lysine 12 (H2BK12ac), and C) acetylation of H3 on lysine 79 (H3K79me2) are detected on the transcriptionally-silenced P2 cell nuclei. The scale bar represents 10 µm and applies to all panels. (TIF) [file pgen.1004588.s007.tif]

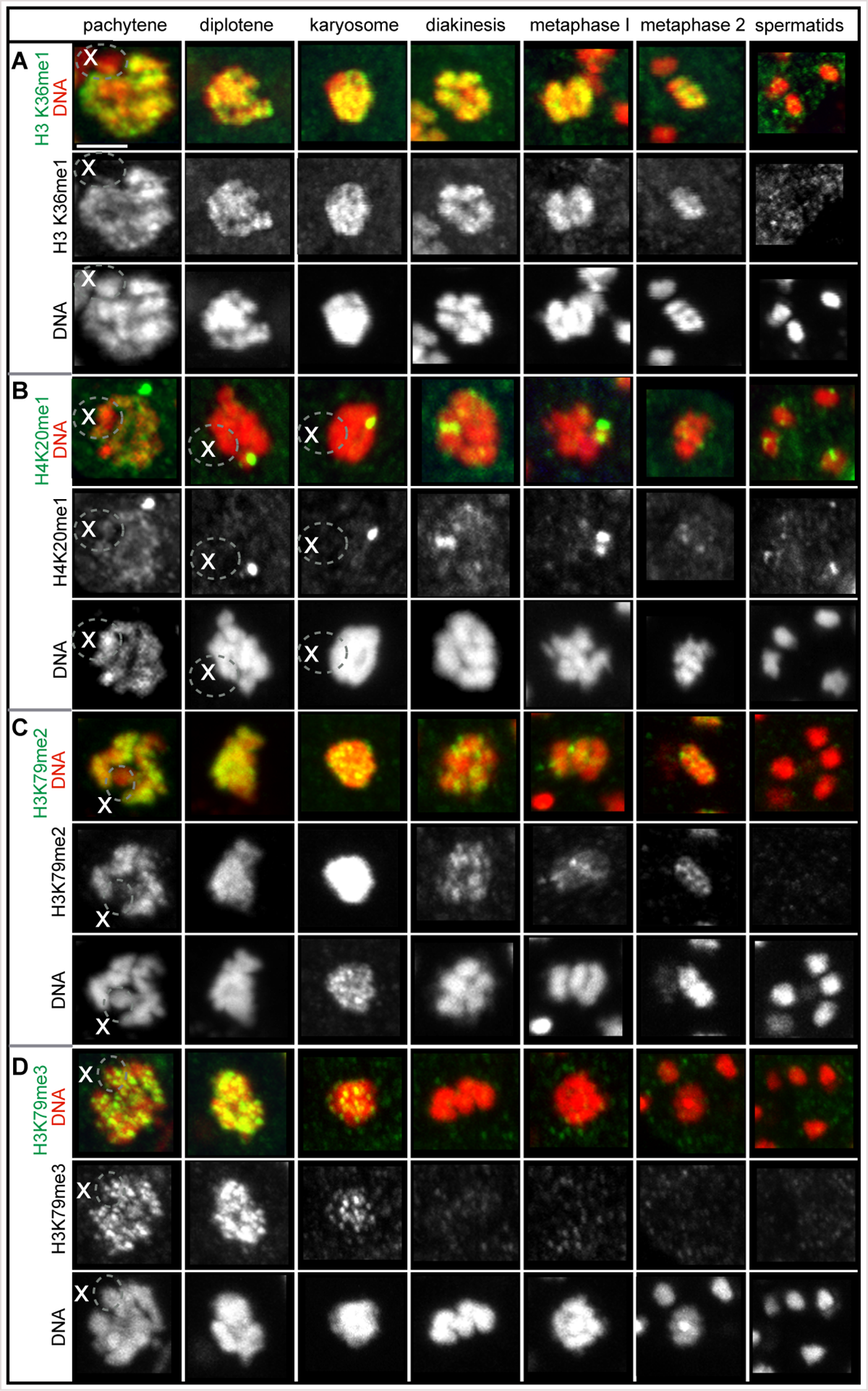

Supplement: Figure S8 — Histone mono-methylation at H3K36, H4K20, and di-methylation at H3K79 are retained in sperm chromatin. Immunostaining of dissected and fixed male gonads with antibodies specific to methylated histones (green) and DNA stained with DAPI (red). The position of the X chromosome (X), which was determined by H3K9me2 co-staining (not shown), is circled. A) H3K36me1 levels remain high through sperm meiotic divisions with low levels of staining on spermatid DNA. B) H4K20me1 levels on chromatin fall prior to the diplotene stage. Bright foci of staining not associated with the X chromosome are visible in nuclei from the pachytene stage through spermatids. C) H3K79me2 levels are high through sperm meiotic divisions but not detectable on spermatid DNA. D) Levels of H3K79me3 become undetectable on chromosomes after the karyosome stage. (TIF) [file pgen.1004588.s008.tif]
