# Supplementary material for: The Specification and Global Reprogramming of Histone Epigenetic Marks during Gamete Formation and Early Embryo Development in C. elegans
Source: PLoS Genet. 2014 Oct 9;10(10):e1004588. doi: 10.1371/journal.pgen.1004588 (PMC4191889; doi:10.1371/journal.pgen.1004588)

## Slide 1
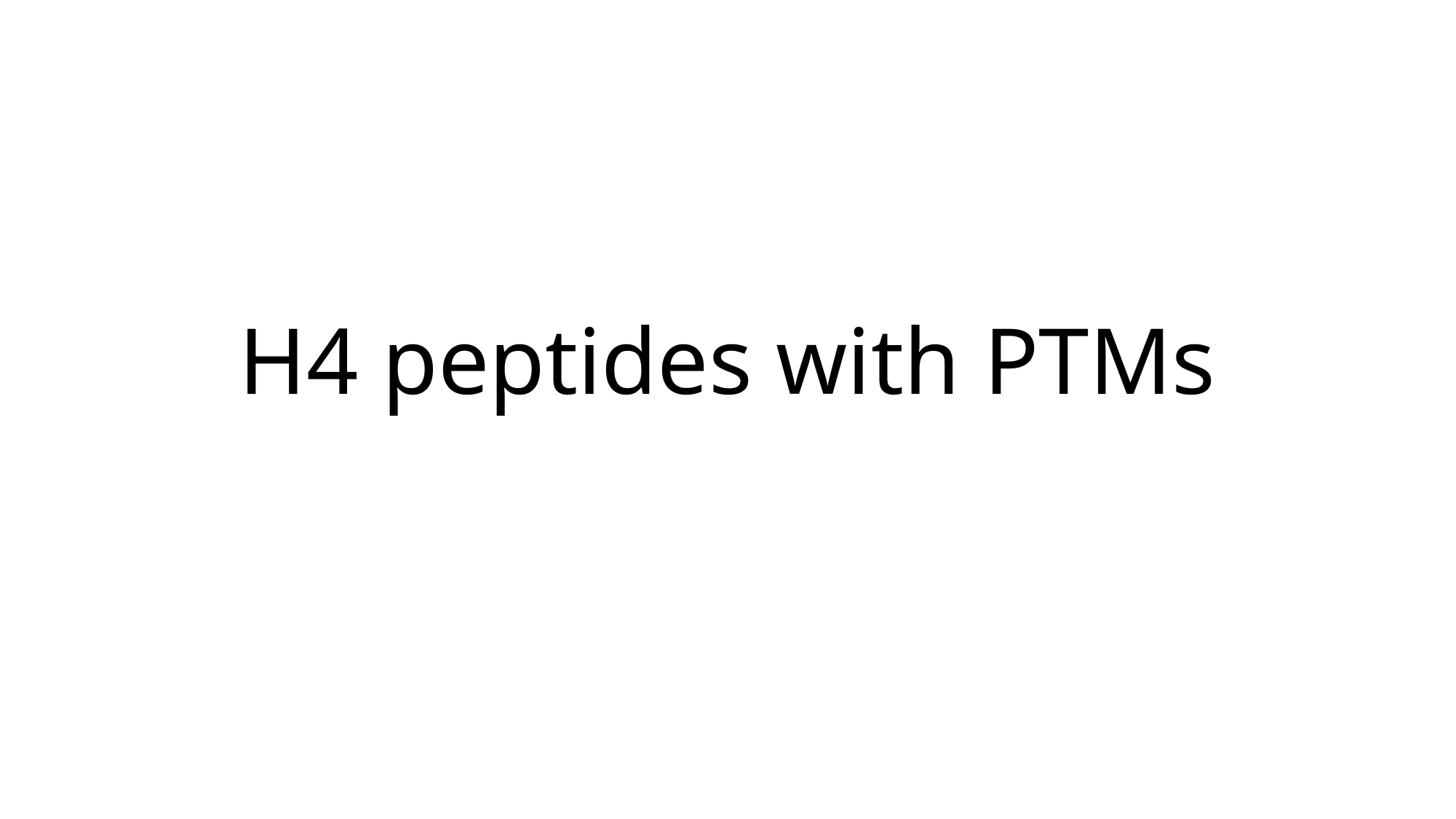

# H4 peptides with PTMs

## Slide 2
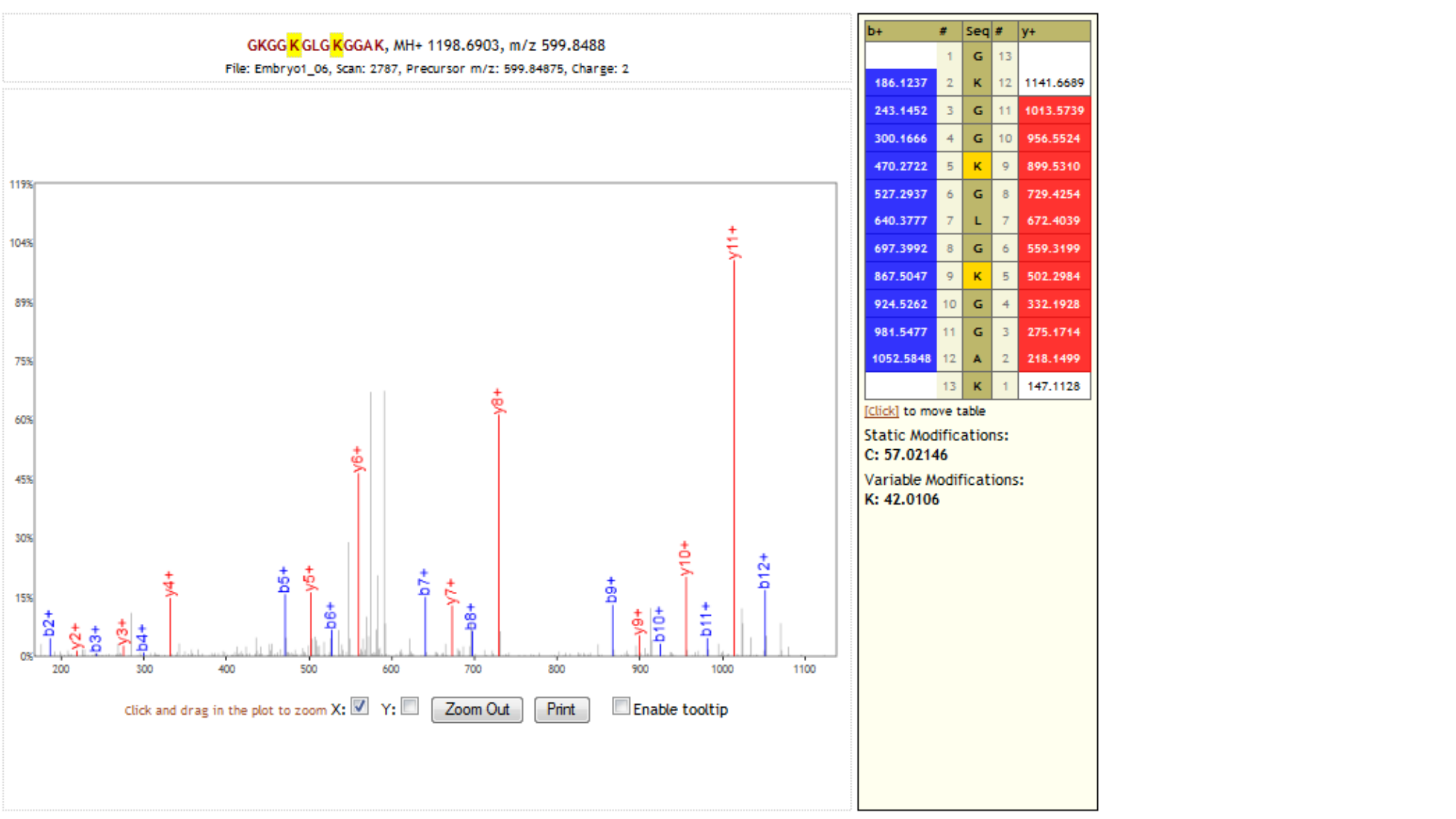

## Slide 3
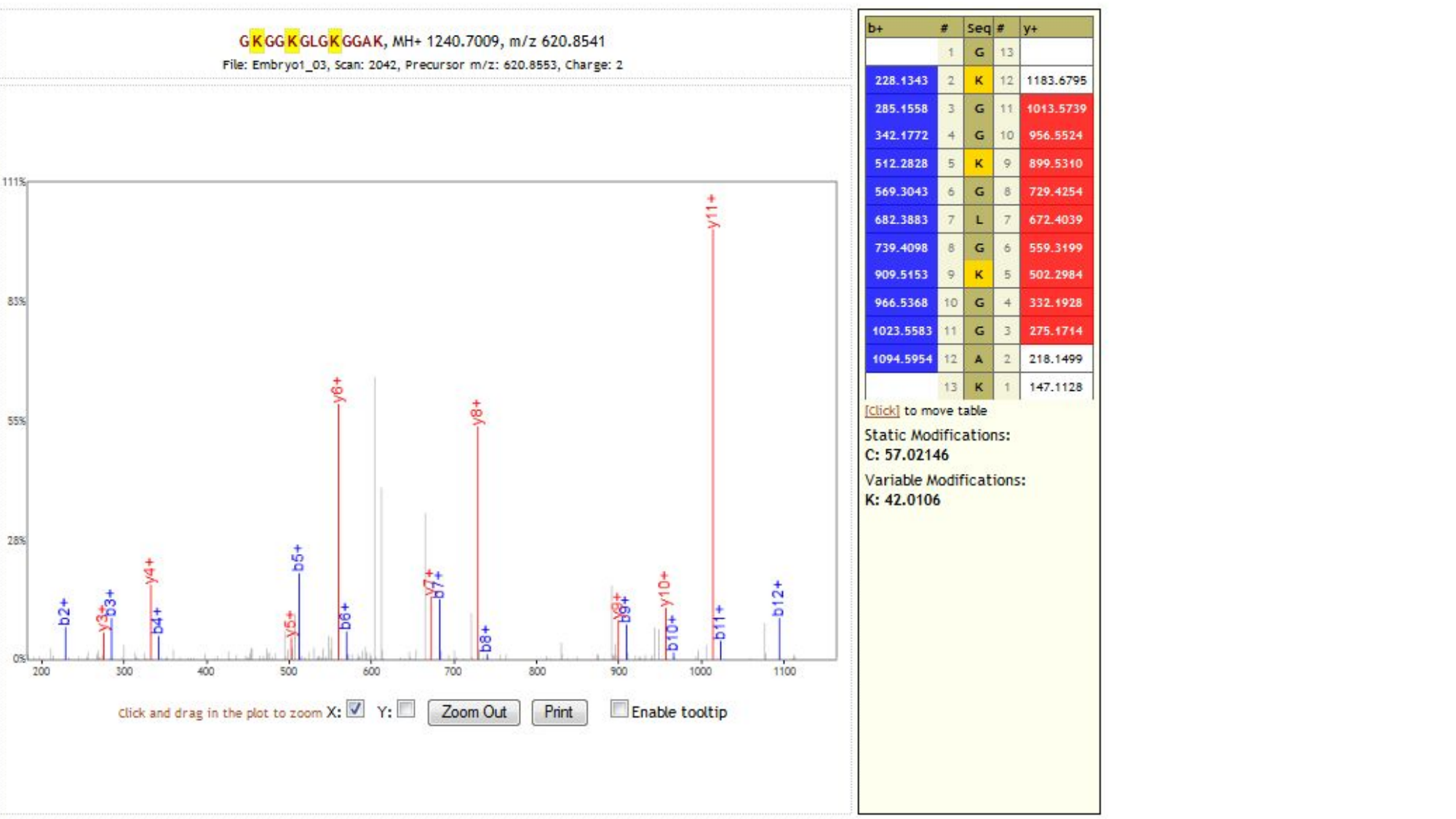

## Slide 4
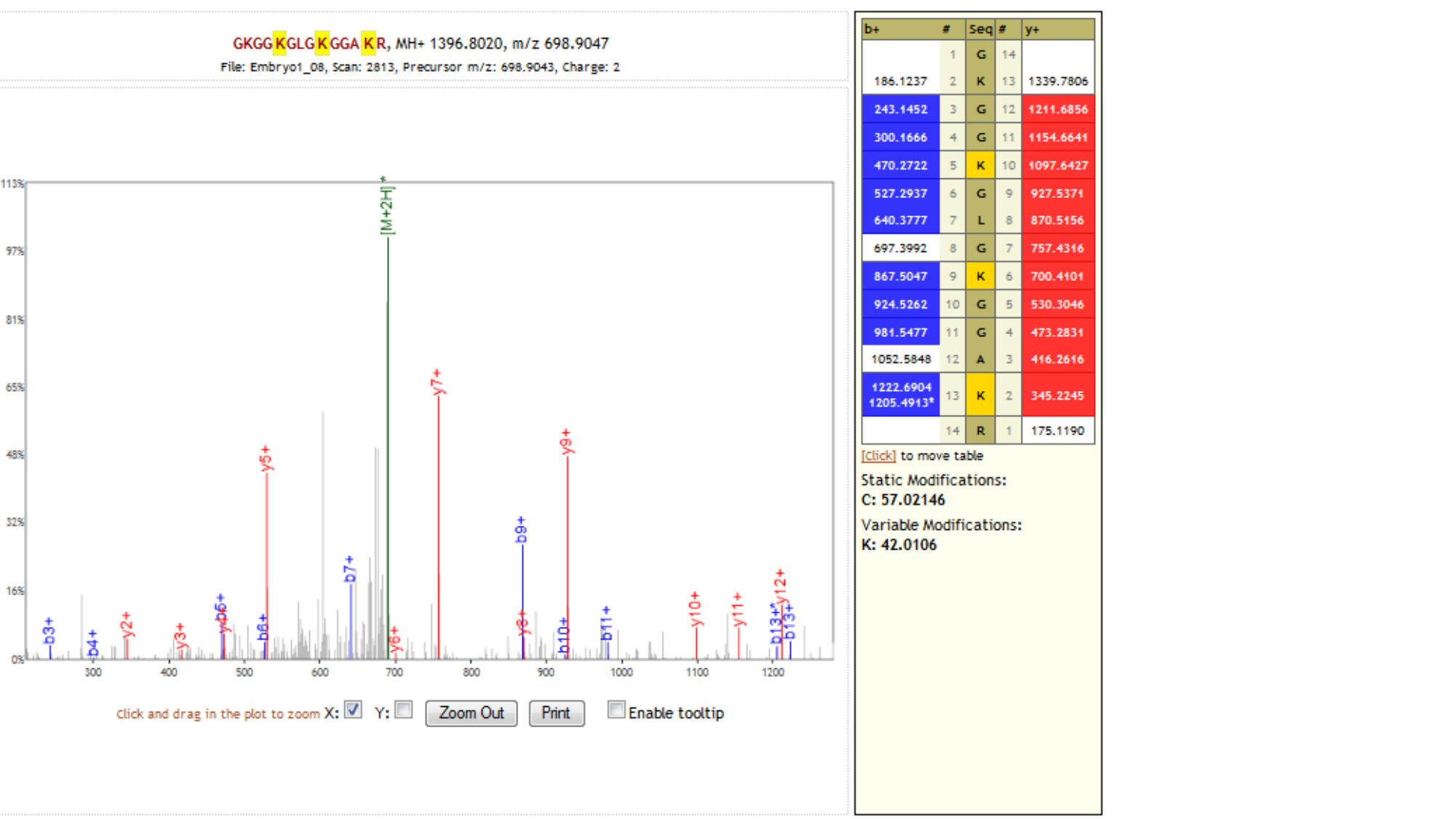

## Slide 5
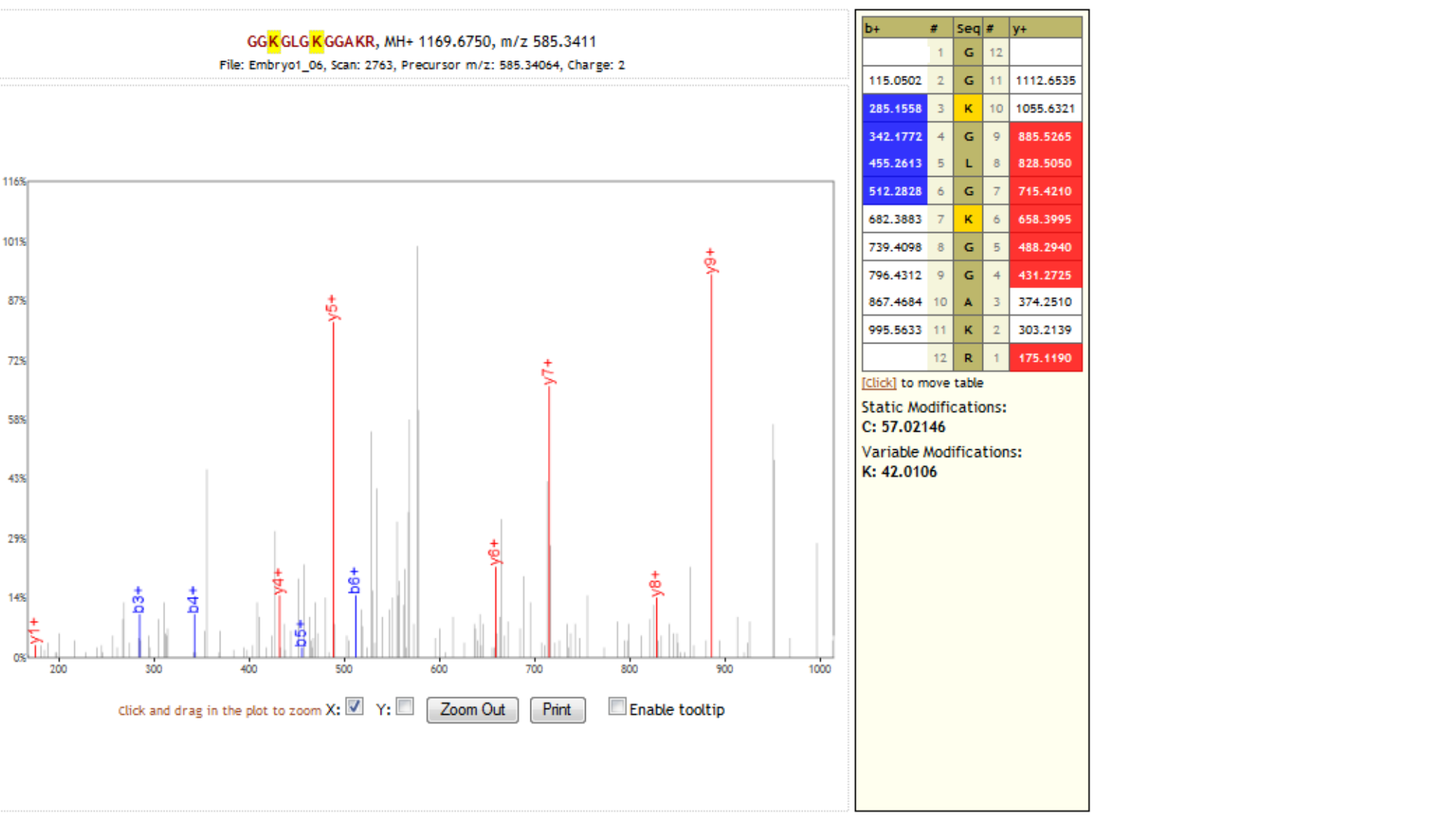

## Slide 6
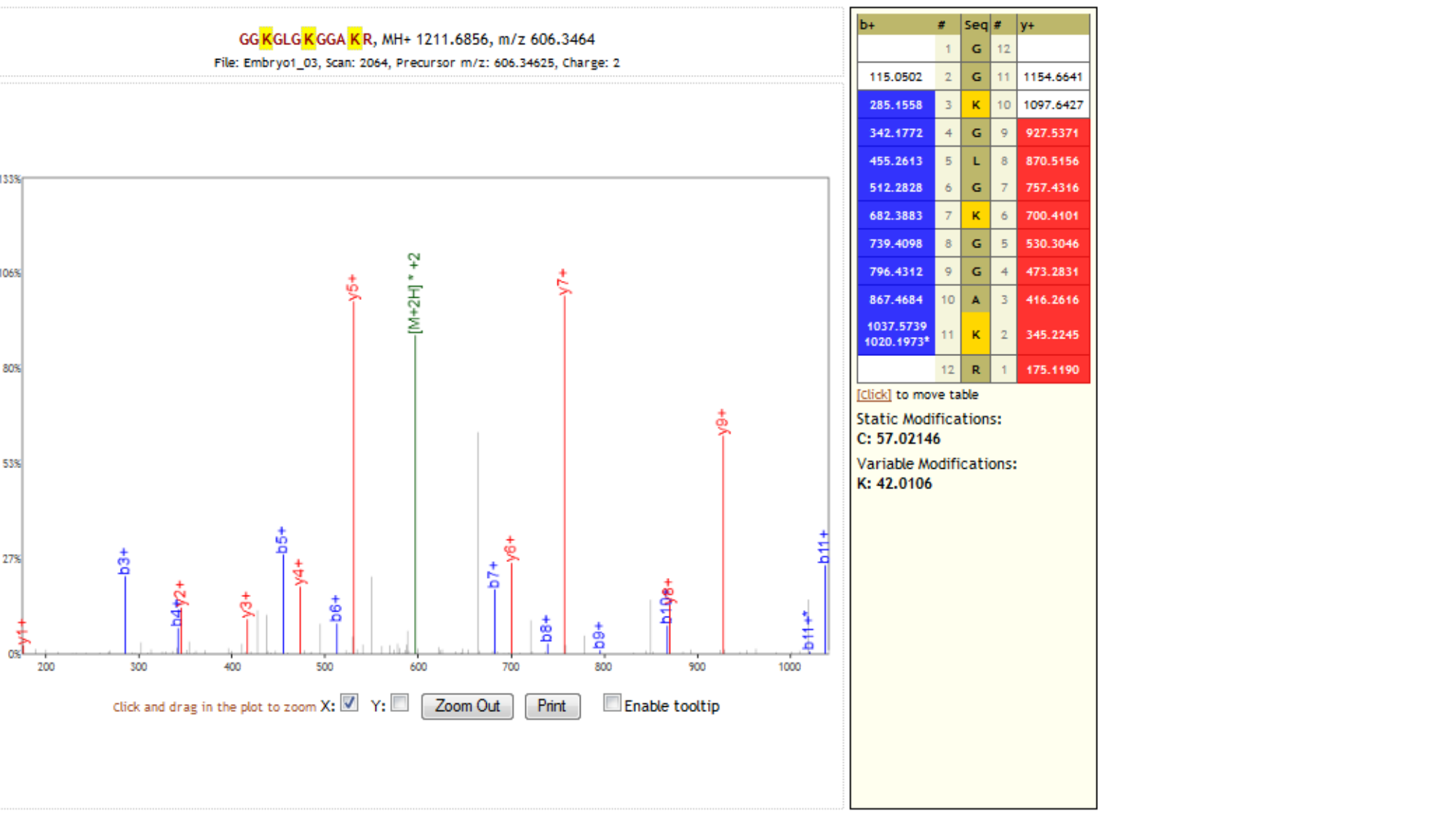

## Slide 7
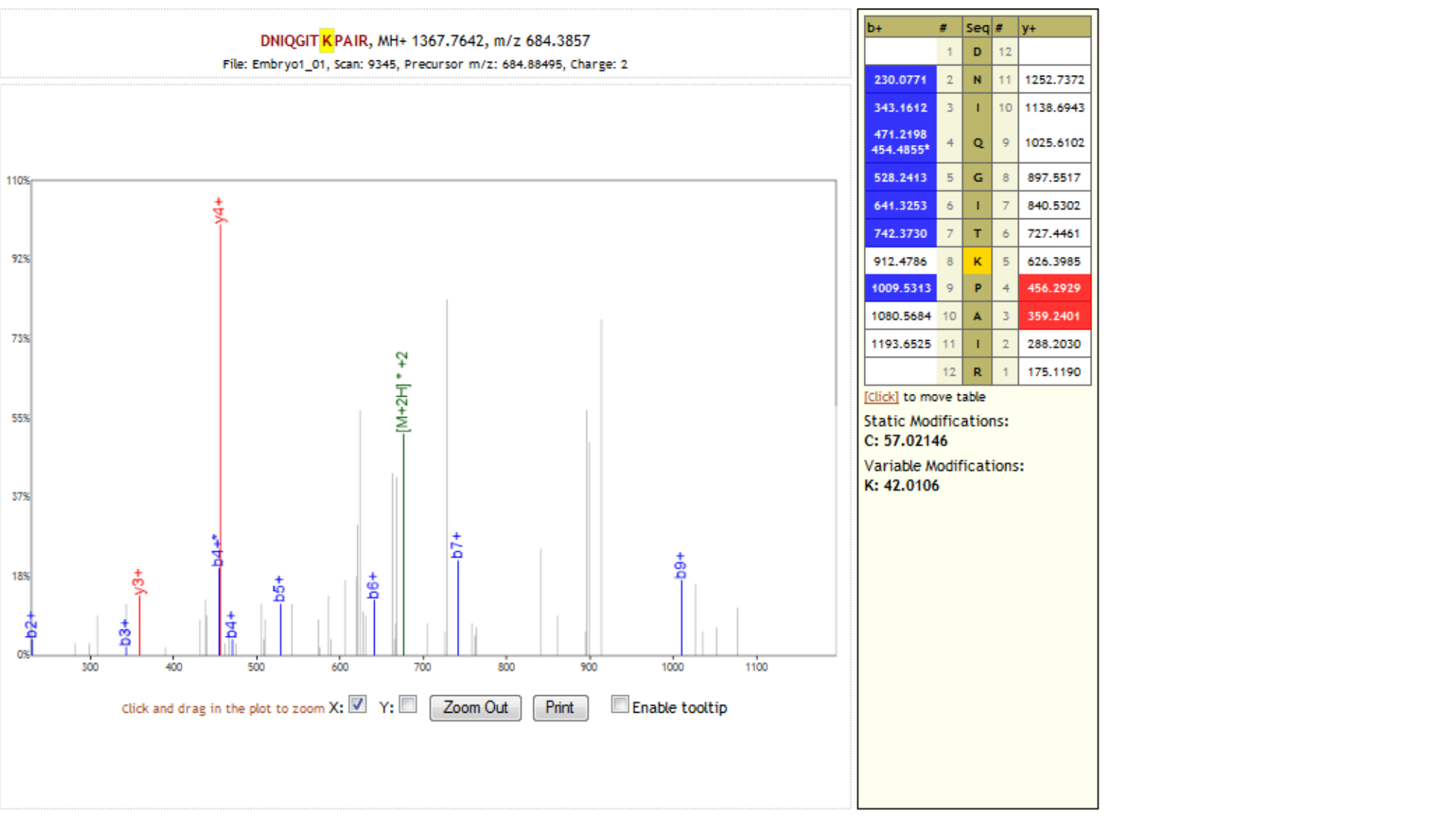

## Slide 8
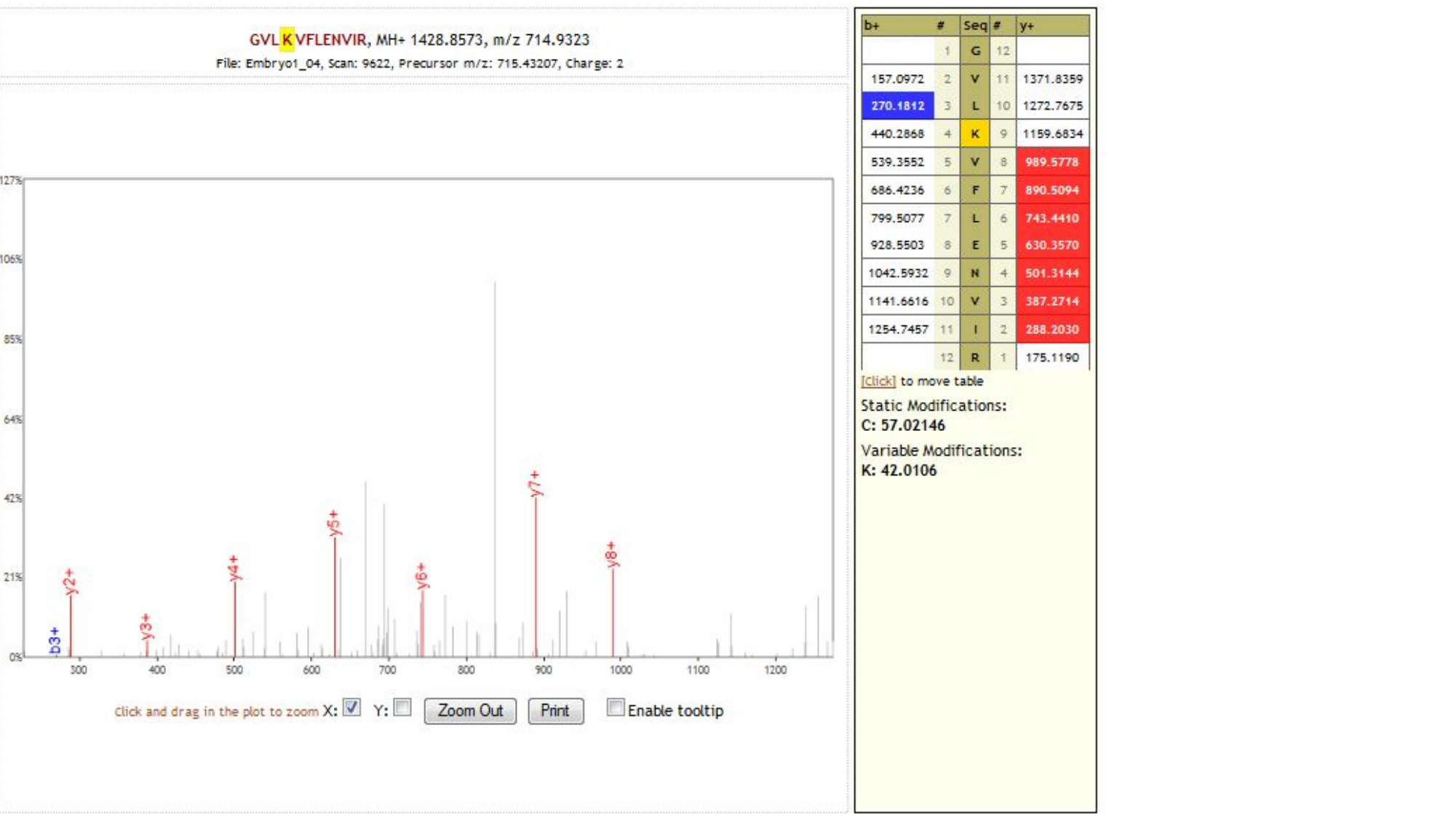

## Slide 9
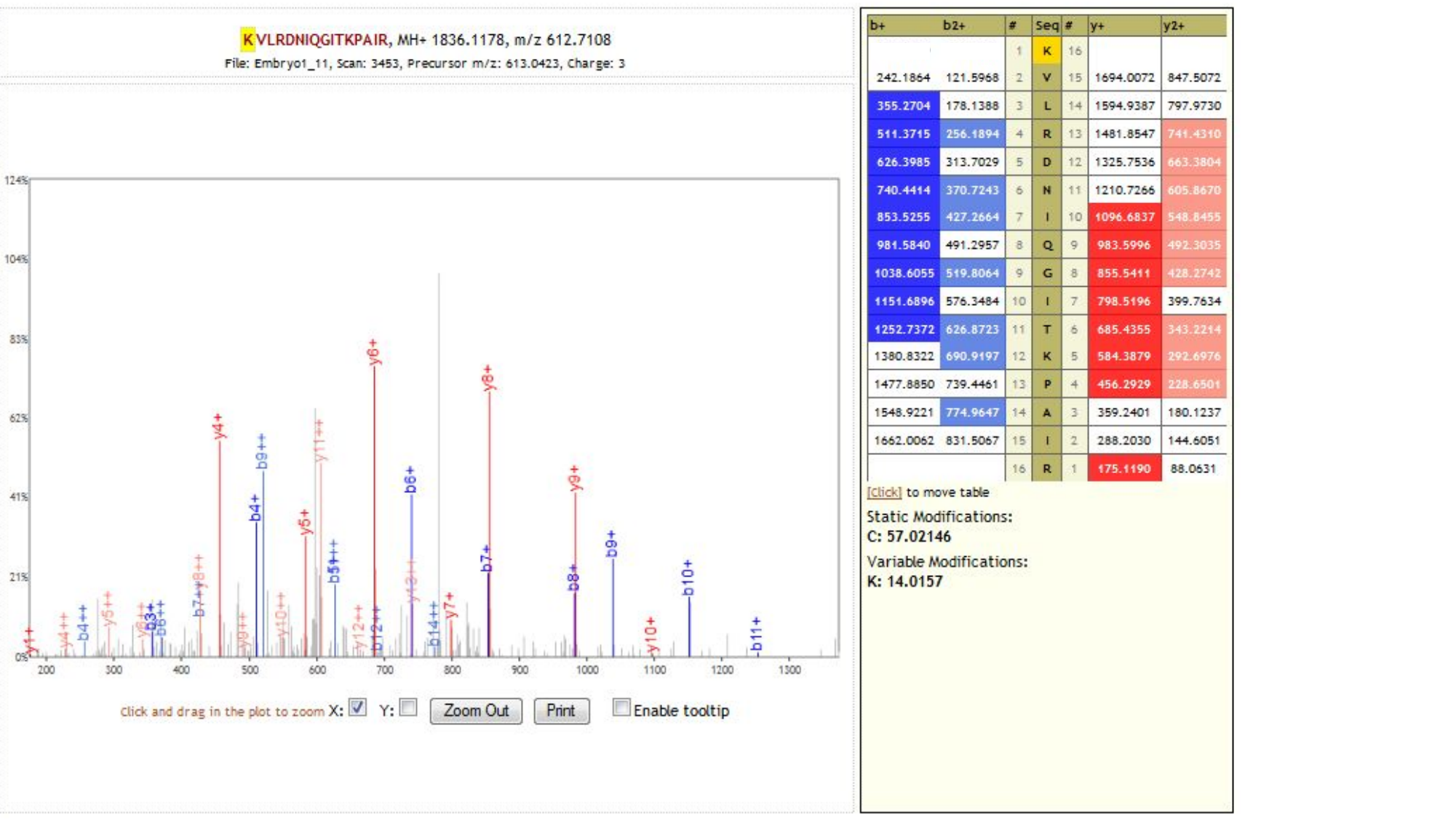

## Slide 10
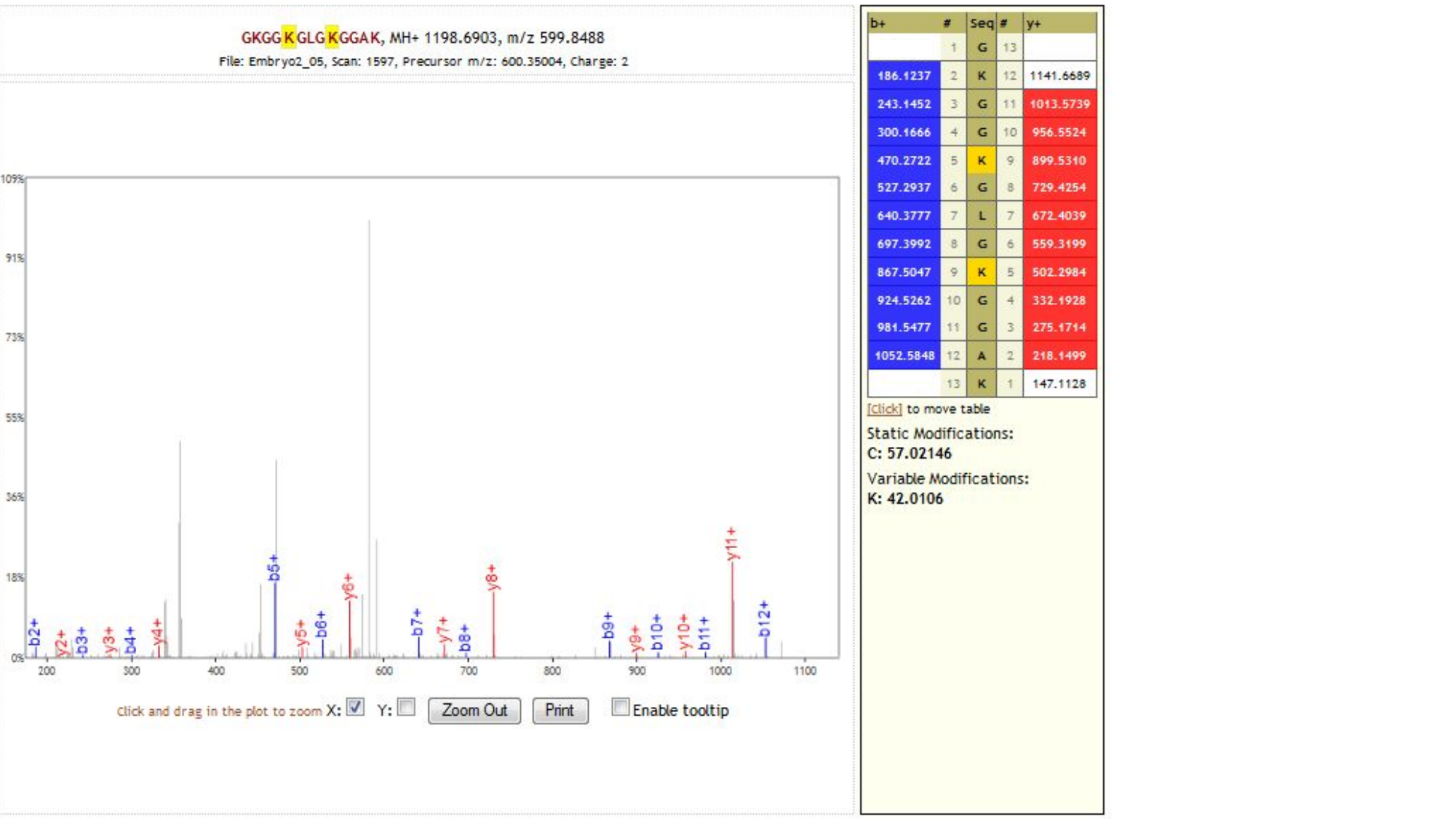

## Slide 11
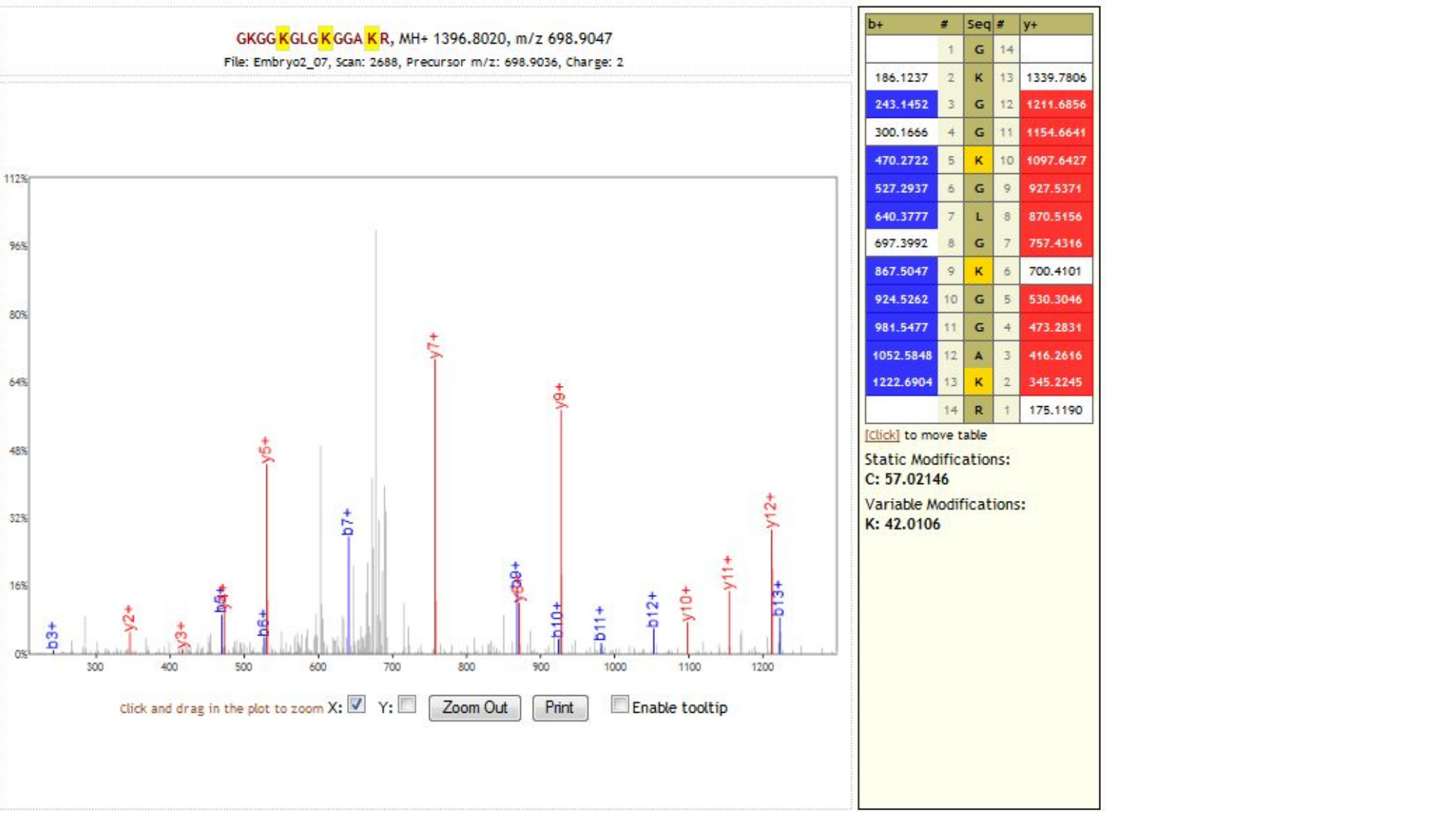

## Slide 12
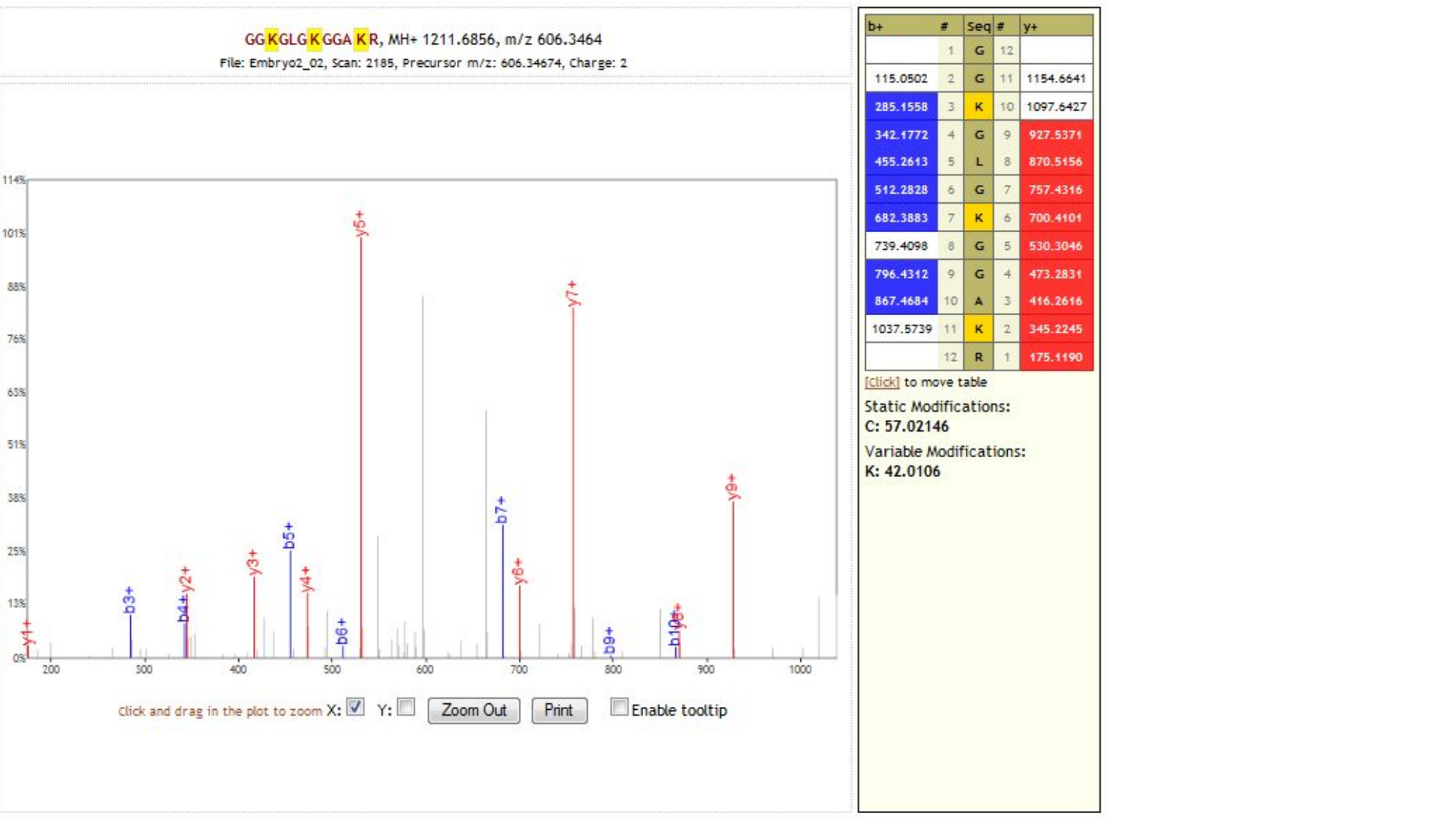

## Slide 13
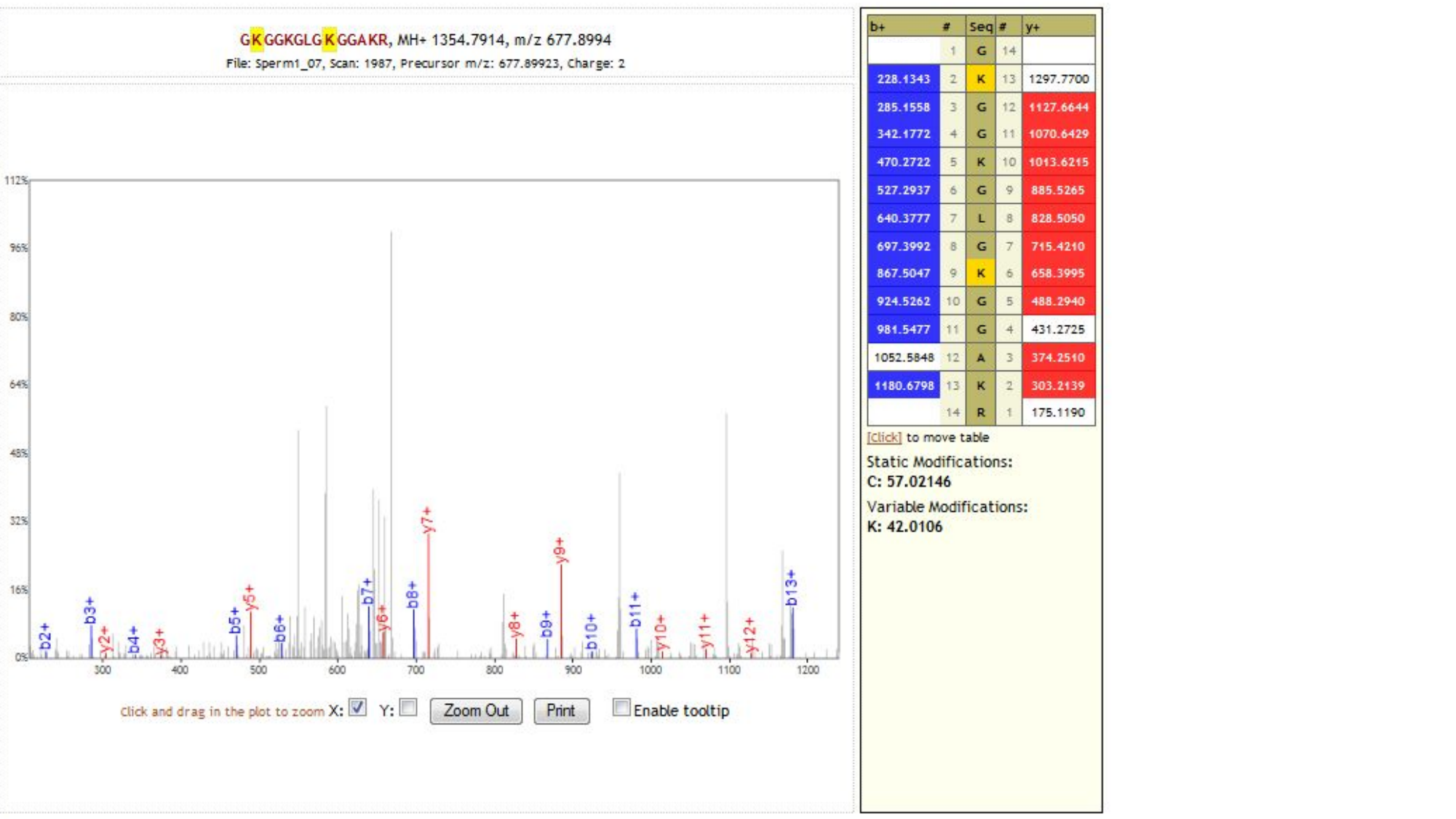

## Slide 14
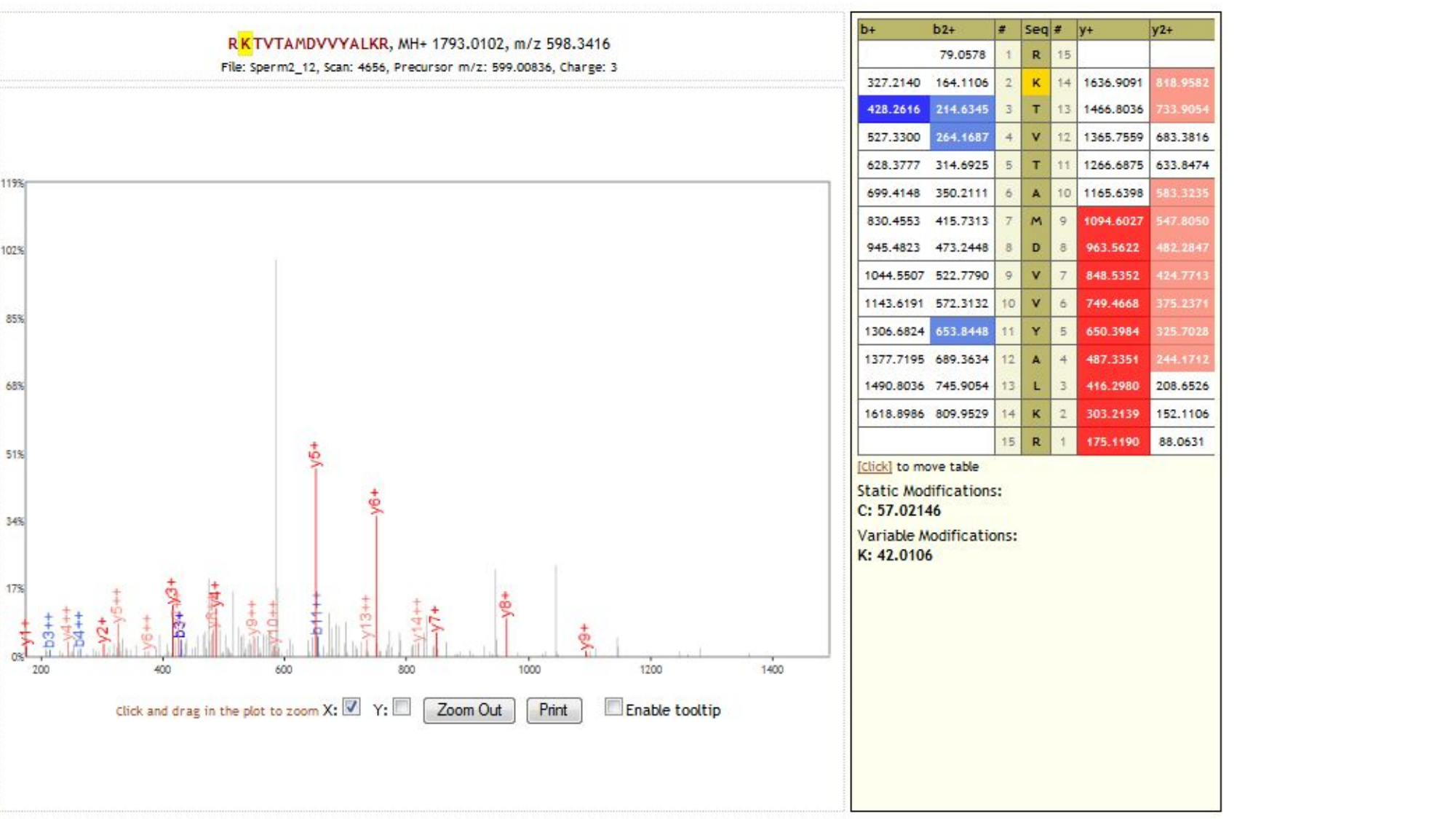

## Slide 15
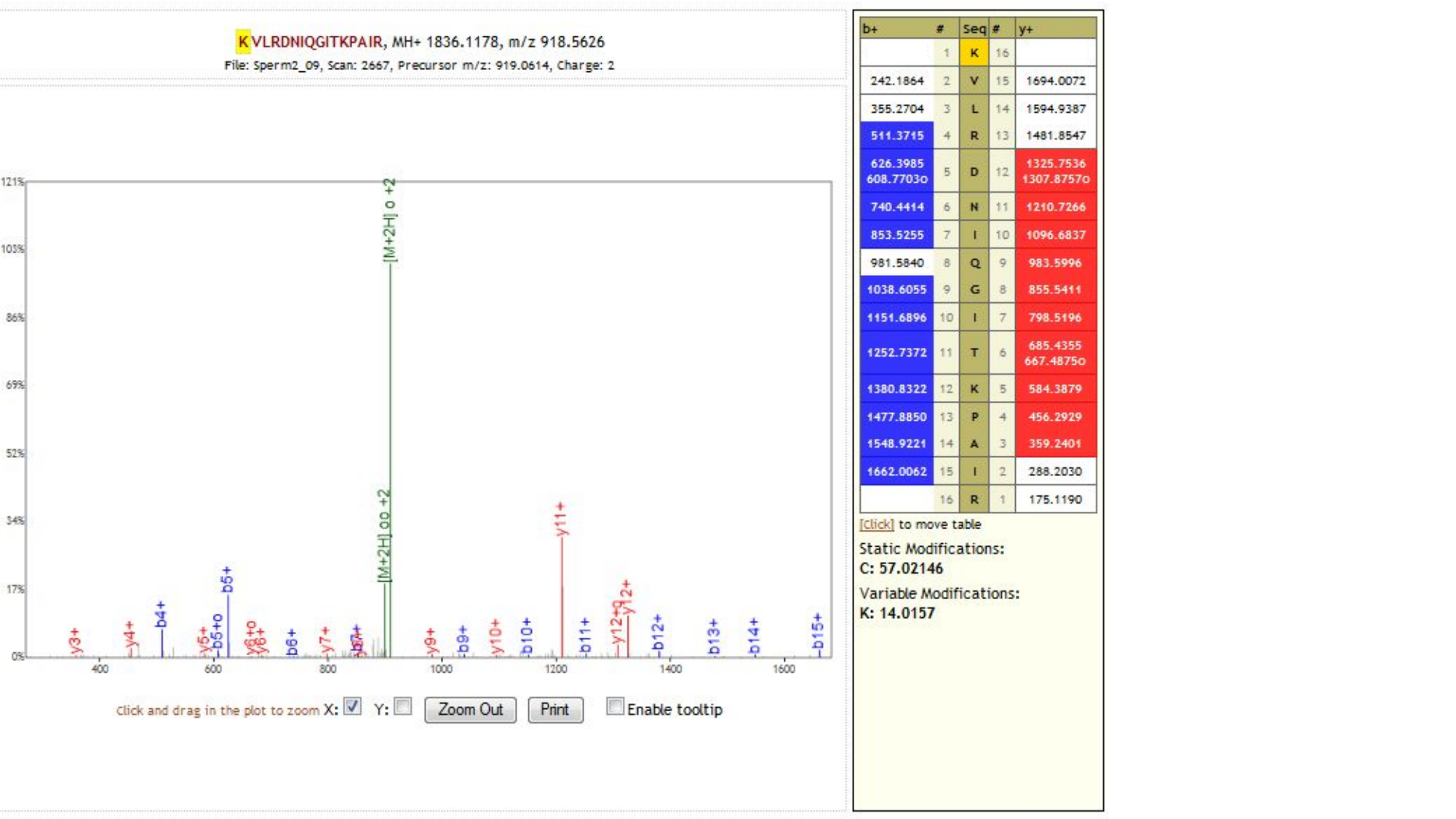

## Slide 16
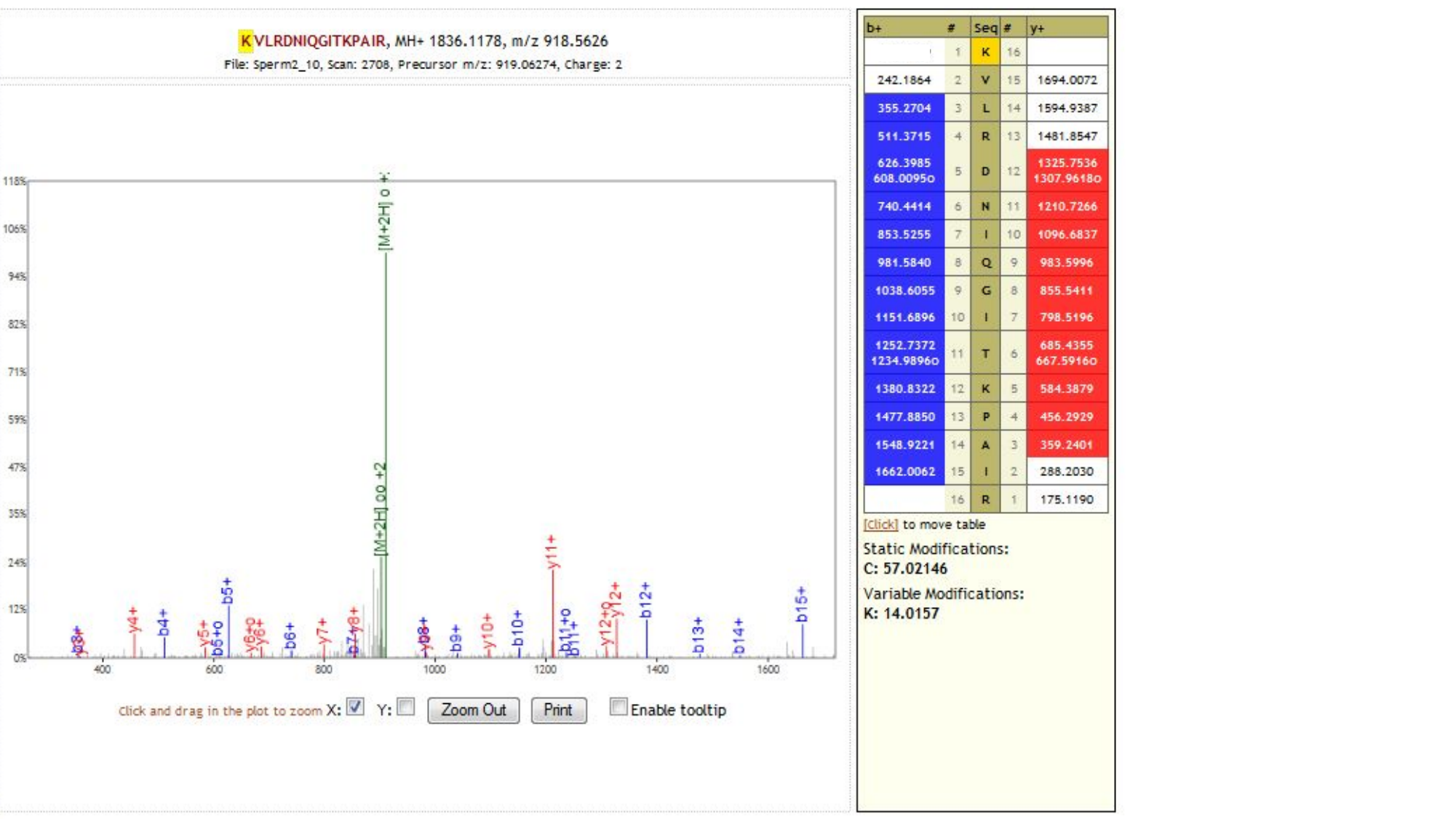

## Slide 17
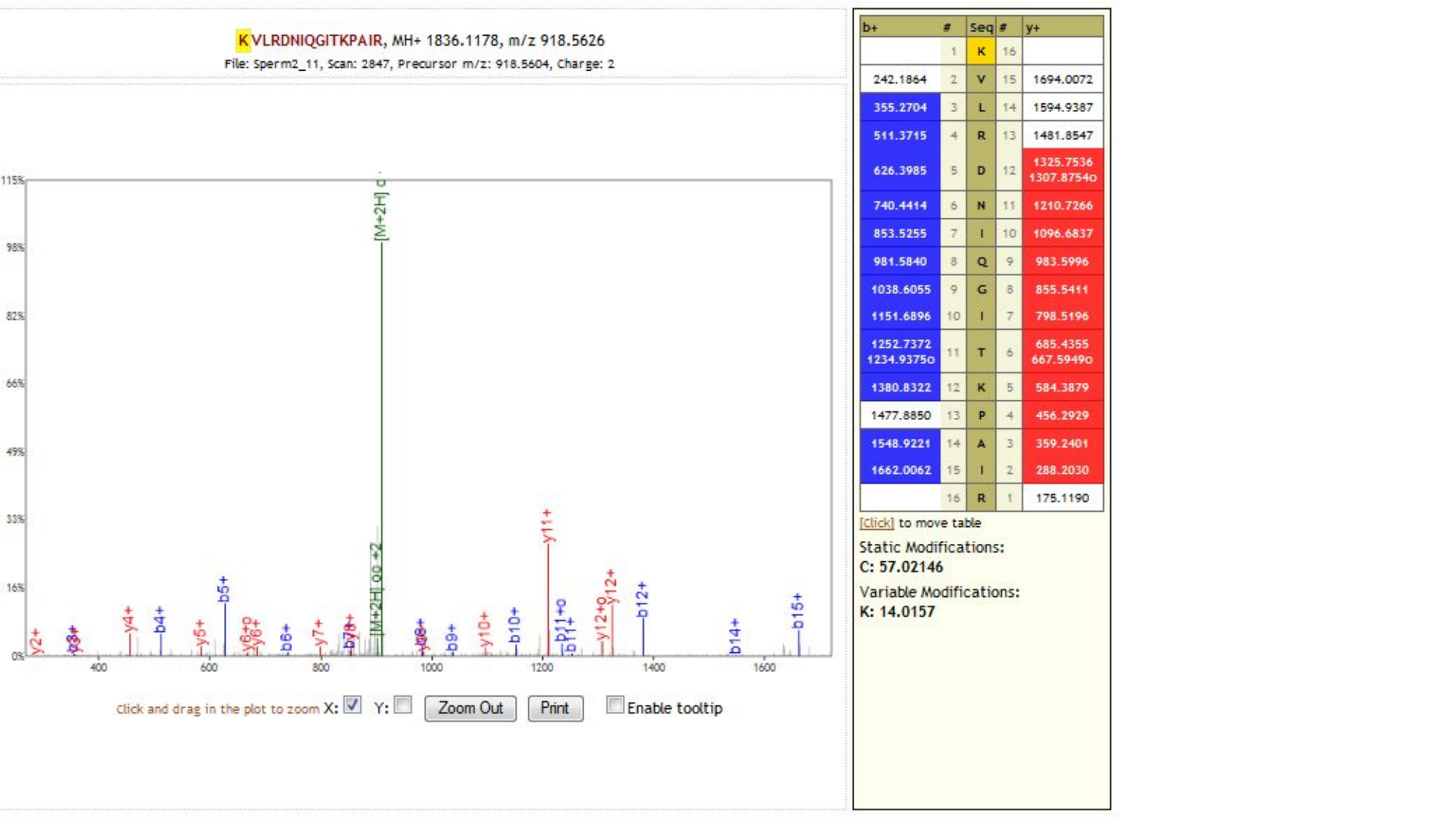

## Slide 18
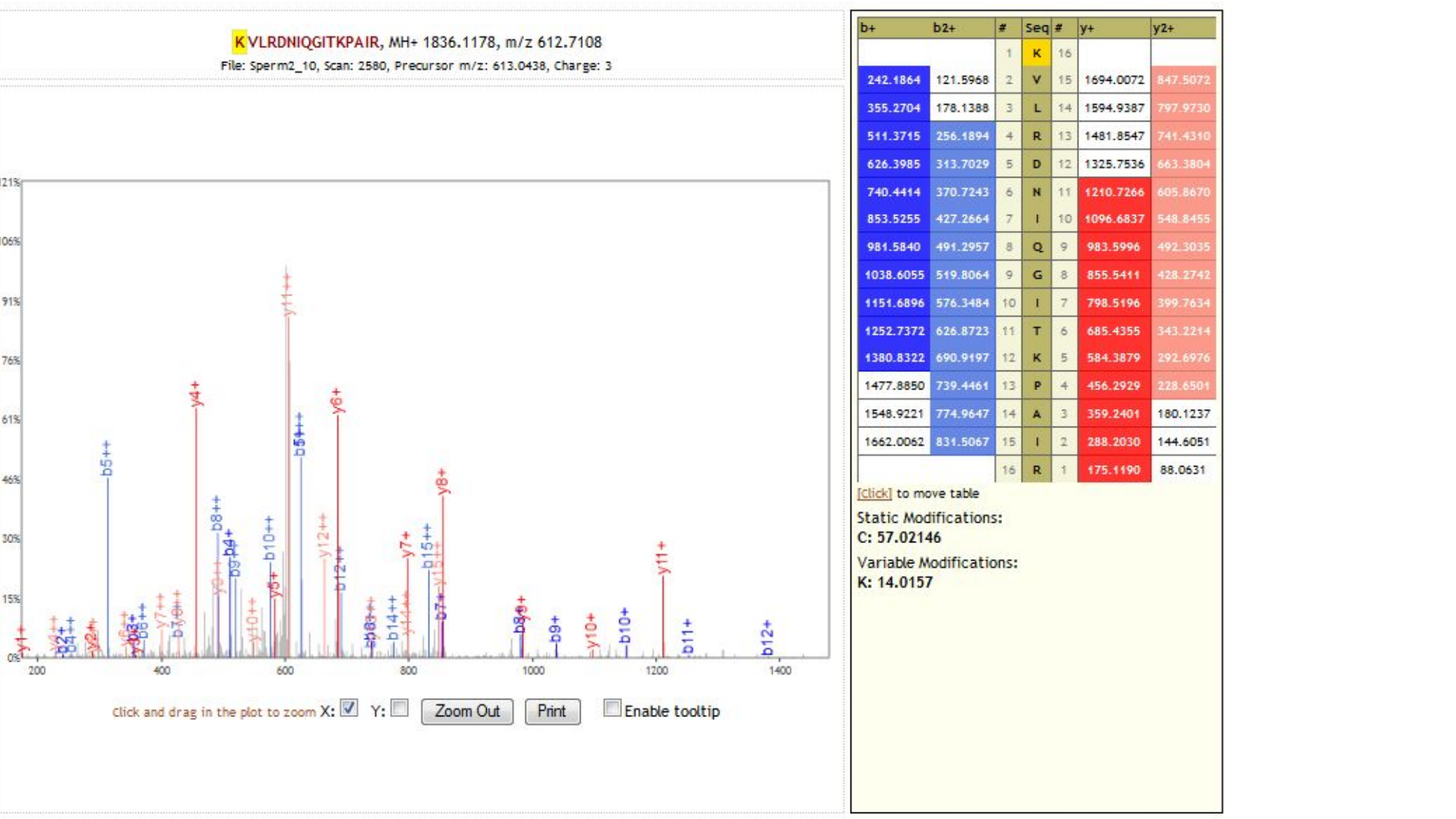

## Slide 19
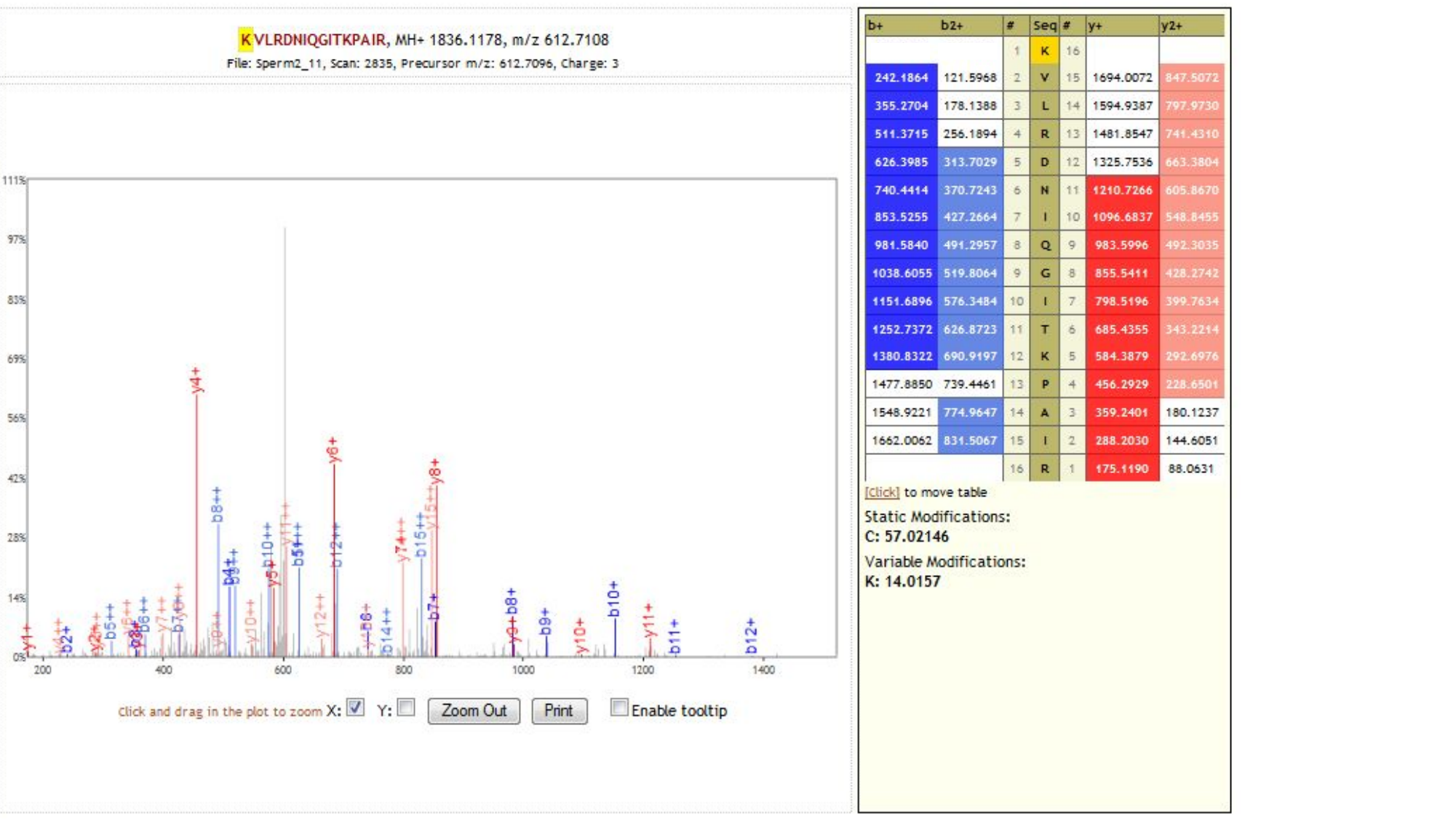

## Slide 20
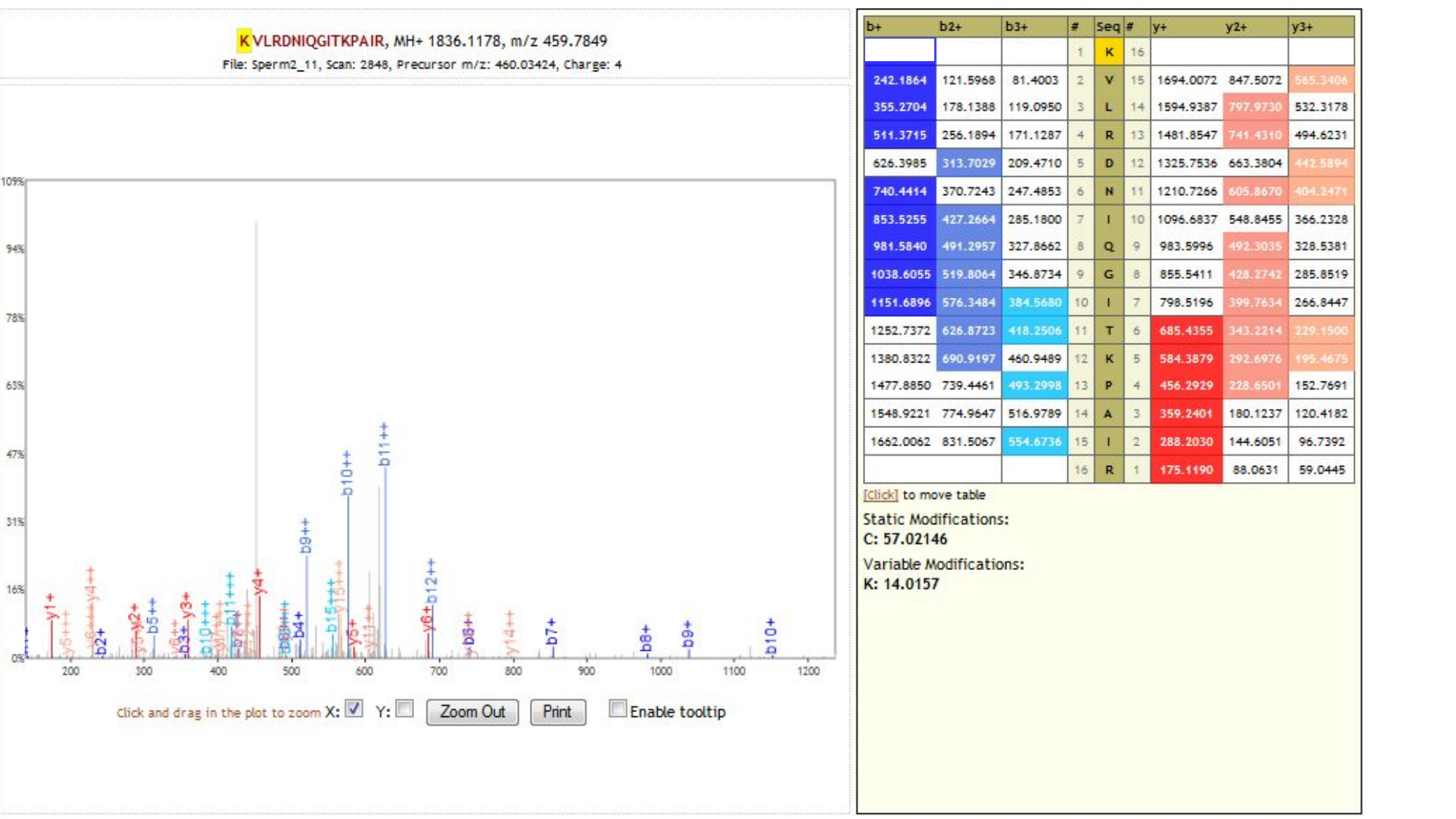

## Slide 21
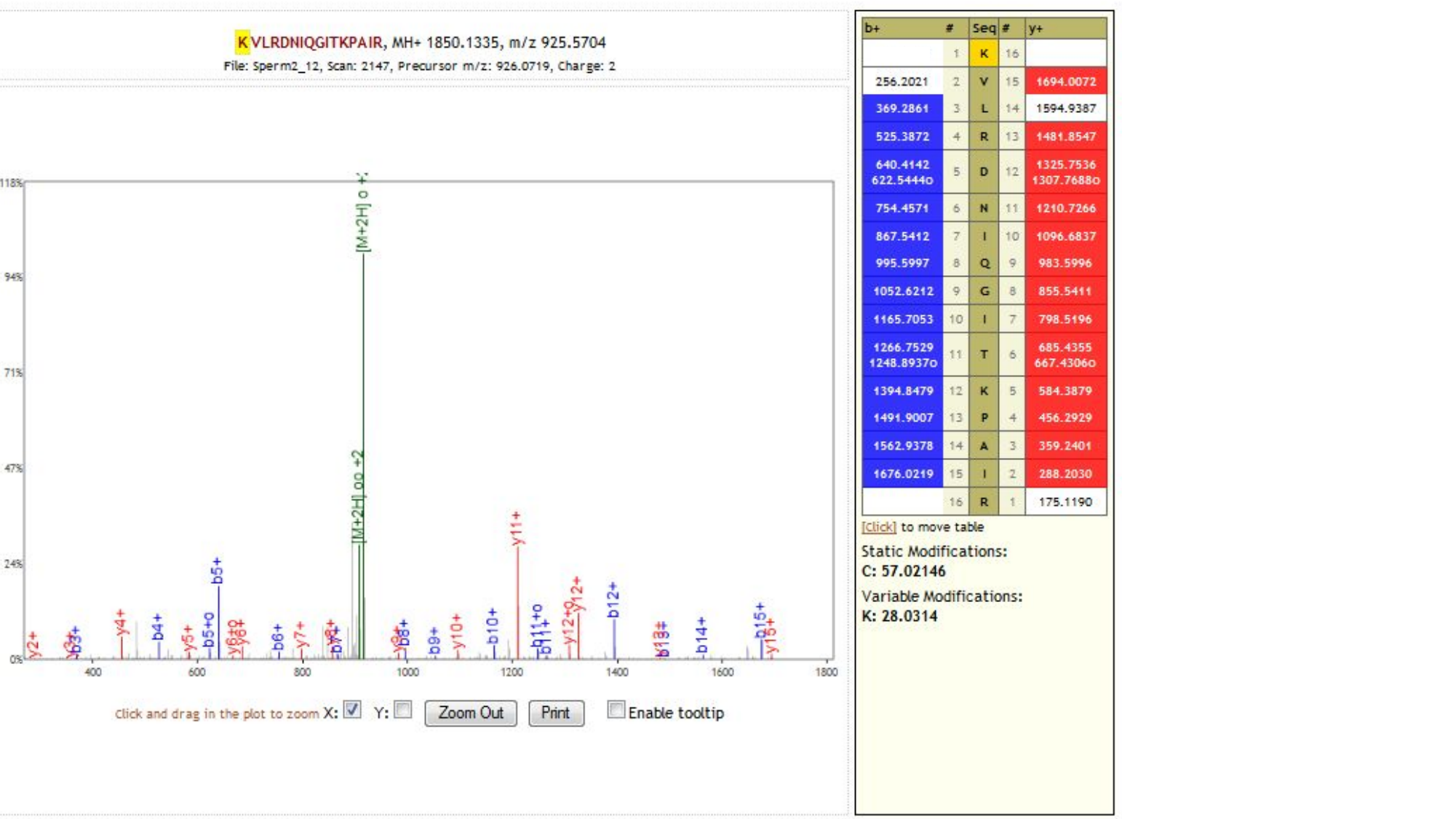

## Slide 22
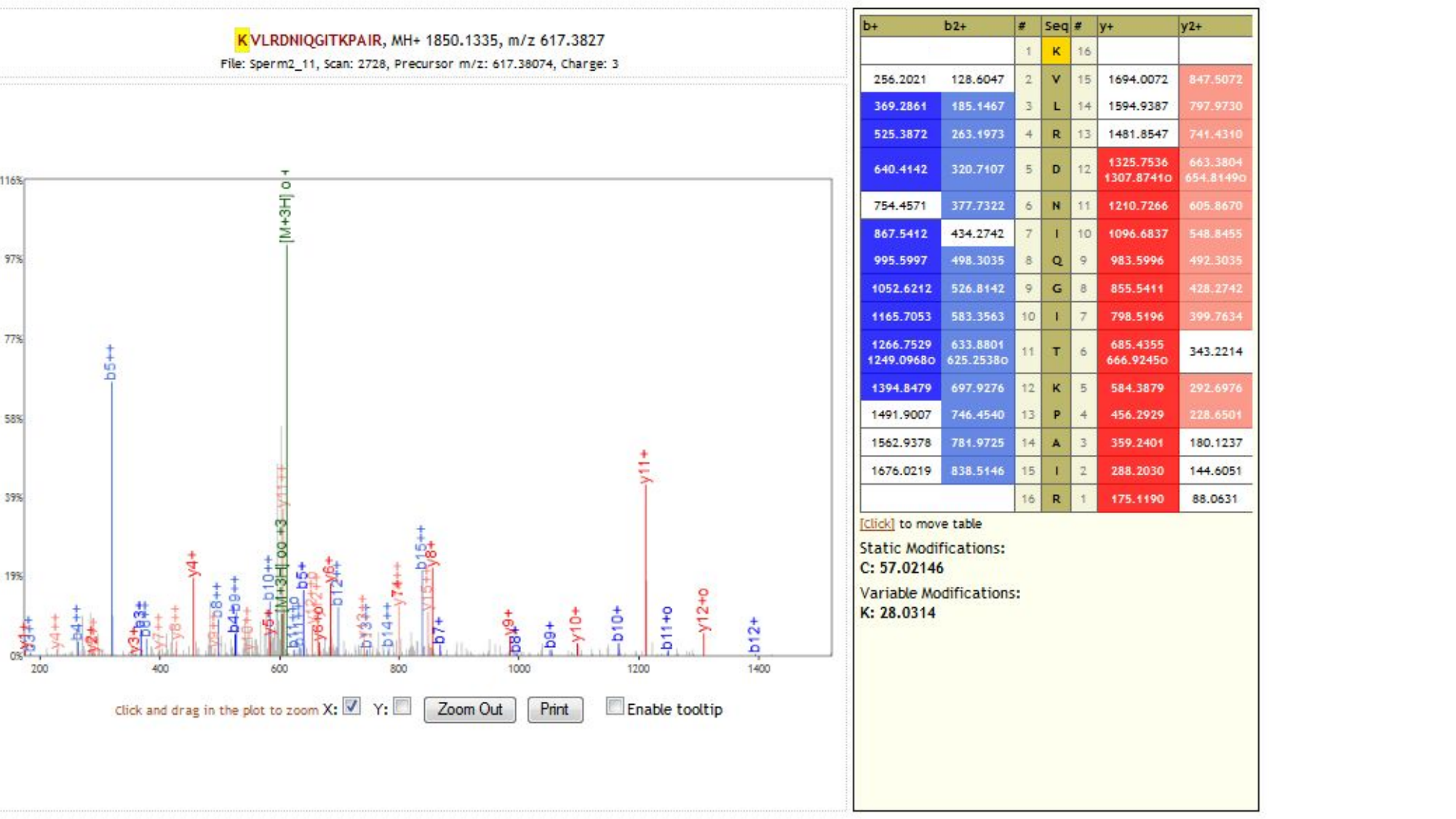

## Slide 23
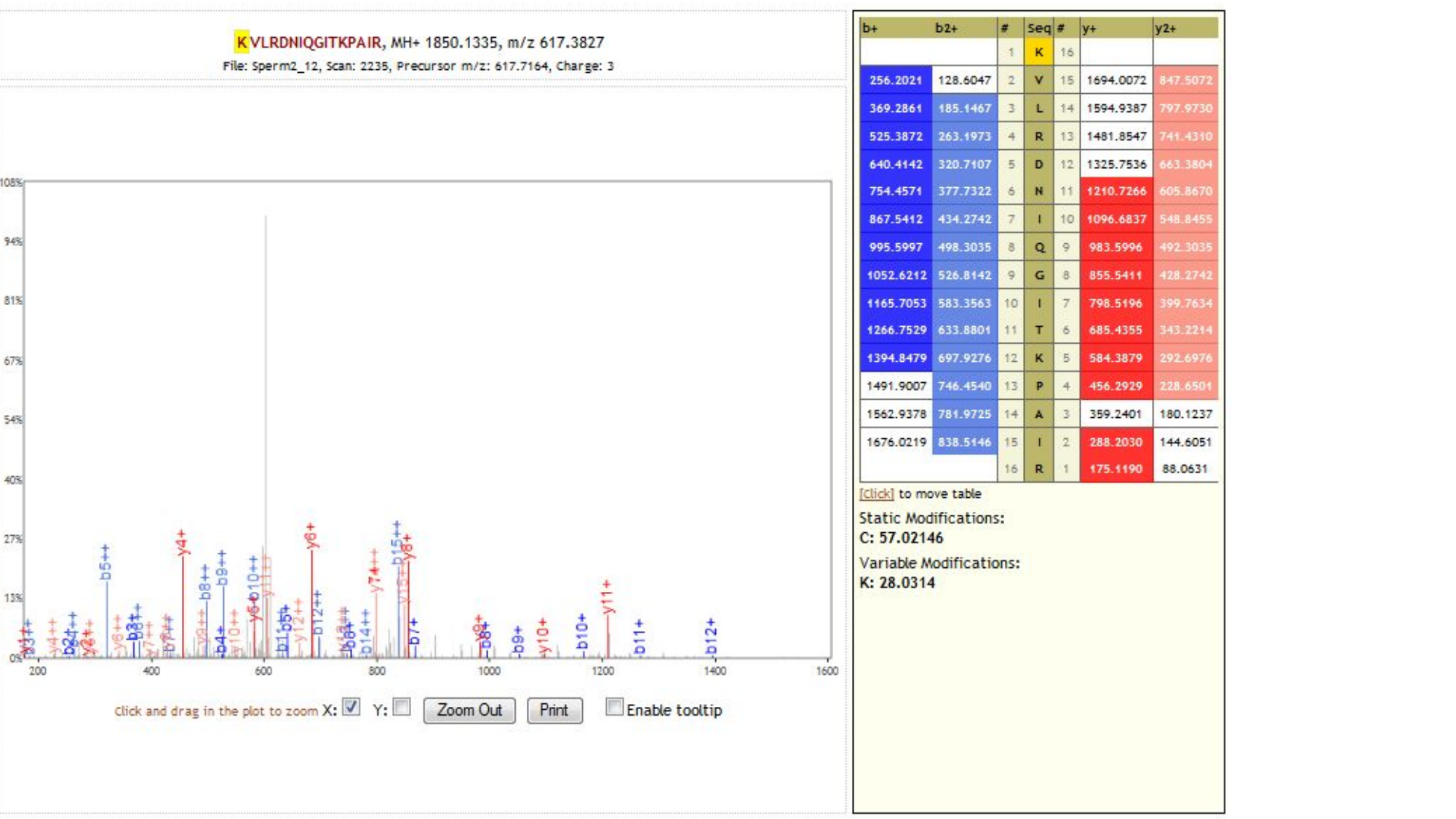

Supplement: File S4 — Annotated mass spectra that identify post-translationally modified histone H4 peptides. Each slide number corresponds to the Slide # shown in Table S6. (PPTX) [file pgen.1004588.s012.pptx]
