# Supplementary material for: The Specification and Global Reprogramming of Histone Epigenetic Marks during Gamete Formation and Early Embryo Development in C. elegans
Source: PLoS Genet. 2014 Oct 9;10(10):e1004588. doi: 10.1371/journal.pgen.1004588 (PMC4191889; doi:10.1371/journal.pgen.1004588)
Supplement: File S5 — Amino acid alignments for C. elegans and mouse histone proteins. We follow the convention of numbering histone proteins starting at the amino acid after the starting methionine. For histone H2A, the red text indicates that K119 in mouse corresponds to K120 in C. elegans. (PDF) [file pgen.1004588.s013.pdf]

Alignments of *C. elegans* and Mouse histone proteins

We follow the convention of numbering histone proteins starting at the amino acid after the starting methionine.

Histone H2A

S-phase H2A is represented by 16 genes that encode identical proteins.

```
                 ----- ----1---------2---------3---------4---------5--------
CeH2A           MSGRGK-GGKAKTGGKAKSRSSRAGLQFPVGRLHRILRKGNYAQRVGAGAPVYLAAVLEY
mouseH2A        MSGRGKQGGKAR--AKAKTRSSRAGLQFPVGRVHRLLRKGNYSERVGAGAPVYLAAVLEY
                 ---------1-  --------2---------3---------4---------5-------
                ****** ****:  .***:*************:**:******::****************

                -6---------7---------8---------9---------0---------1--------
CeH2A           LAAEVLELAGNAARDNKKTRIAPRHLQLAVRNDEELNKLLAGVTIAQGGVLPNIQAVLLP
mouseH2A        LTAEILELAGNAARDNKKTRIIPRHLQLAIRNDEELNKLLGRVTIAQGGVLPNIQAVLLP
                --6---------7---------8---------9---------0---------1-------
                *:**:**************** *******:**********. ******************

                -2------
CeH2A           KKTGGDKE----
mouseH2A        KKTESHHKAKGK
                --2---------
                *** ..::
```

Histone H4

H4 is represented by 16 genes encoding identical proteins.

```
                 ---------1---------2---------3---------4---------5---------
C. elegans H4   MSGRGKGGKGLGKGGAKRHRKVLRDNIQGITKPAIRRLARRGGVKRISGLIYEETRGVLK 60
mouse H4        MSGRGKGGKGLGKGGAKRHRKVLRDNIQGITKPAIRRLARRGGVKRISGLIYEETRGVLK 60
                ************************************************************

                6---------7---------8---------9---------0--
C. elegans H4   VFLENVIRDAVTYCEHAKRKTVTAMDVVYALKRQGRTLYGFG- 102
Mouse H4        VFLENVIRDAVTYTEHAKRKTVTAMDVVYALKRQGRTLYGFGG 103
                ************* ****************************
```

Histone H2B

HIS-48 class includes 5 proteins: B0035.8 (HIS-48), F54E12.4 (HIS-58), F55G1.3 (HIS-62), H02I12.6 (HIS-66)

HIS-11 class includes 4 proteins: ZK131.5 (HIS-11), ZK131.9 (HIS-15), F35H10.11 (HIS-29), F17E9.9 (HIS-34), F08G2.1 (HIS-44)

HIS-8 class includes 3 proteins: F45F2.12 (HIS-8), K06C4.4 (HIS-20), K06C4.12 (HIS-22)

The numbering for *C. elegans* homologs shown is based on the alignment for the HIS-48 class of proteins, which had the most number of unique peptide IDs.

```
                                    ---------1---------2---------3---------4
HIS-48 class    -------------------MPPKPSAKGAKKAAKTVVAKPKDGKKRRHARKESYSVYIYR 41
HIS-11 class    -------------------MPPKPSAKGAKKAAKTVT-KPKDGKKRRHARKESYSVYIYR 40
HIS-8 class     ------------------MAPPKPSAKGAKKAAKTVT-KPKDGKKRRHARKESYSVYIYR 41
C50F4.5         ------------------MAPPKPSAKGAKKAAKTVS-KPKDGKKRKHARKESYSVYIYR 41
T10C6.11        MAINPFFTLVQRPVQSIEMAPPKPSAKGAKKAAKTVT-KPKDGKKRRHARKESYSVYIYR 59
F07B7.4/11      MANNPFLTLVQRPVQSIEMAPPKPSAKGAKKAAKTVT-KPKDGKKRRHARKESYSVYIYR 59
F45F2.2         --------------------------MNCCKIFKYLT--------PNFLHKESYSLYIFR 26
Mouse H2B       -------------MPEPS-KSAPAPKKGSKKAISKAQ--KKDGKKRKRSRKESYSVYVYK 44
                              ---- -----1---------2--  -------3---------4---
                                           .  *  .            .  :*****:*:::

                ---------5---------6---------7---------8---------9---------0
HIS-48 class    VLKQVHPDTGVSSKAMSIMNSFVNDVFERIASEASRLAHYNKRSTISSREIQTAVRLILP 101
HIS-11 class    VLKQVHPDTGVSSKAMSIMNSFVNDVFERIAAEASRLAHYNKRSTISSREIQTAVRLILP 100
HIS-8 class     VLKQVHPDTGVSSKAMSIMNSFVNDVFERIAAEASRLAHYNKRSTISSREIQTAVRLILP 101
C50F4.5         VLKQVHPDTGVSSKAMSIMNSFVNDVFERIASEASRLAHYNKRSTISSREIQTAVRLILP 101
T10C6.11        VLKQVHPDTGVSSKAMSIMNSFVNDVFERIAAEASRLAHYNKRSTISSREIQTAVRLILP 119
F07B7.4/11      VLKQVHPDTGVSSKAMSIMNSFVNDVFERIAAEASRLAHYNKRSTISSREIQTAVRLILP 119
F45F2.2         VLKQVHPDTGVSSKAMSIMNSFVNDVFERIAAEASRLAHYNKRSTISSREIQTAVRLILP 86
Mouse H2B       VLKQVHPDTGISSKAMGIMNSFVNDIFERIASEASRLAHYNKRSTITSREIQTAVRLLLP 104
                ------5---------6---------7---------8---------9---------0---
                **********:*****.********:*****:**************:**********:**

                ---------1---------2--
HIS-48 class    GELAKHAVSEGTKAVTKYTSSK 123
HIS-11 class    GELAKHAVSEGTKAVTKYTSSK 122
HIS-8 class     GELAKHAVSEGTKAVTKYTSSK 123
C50F4.5         GELAKHAVSEGTKAVTKYTSSK 123
T10C6.11        GELAKHAVSEGTKAVTKYTSSK 141
F07B7.4/11      GELAKHAVSEGTKAVTKYTSSK 141
F45F2.2         GELAKNAVSEGTNAVTKYTSSK 108
Mouse H2B       GELAKHAVSEGTKAVTKYTSSK 126
                ------1---------2-----
                ************:*********
```

Histone H3

S-phase H3 is encoded by 14 genes: (F22B3.2 (his-63),F55G1.2 (his-59), F54E12.1 (his-55), F07B7.5 (his-49), B0035.10 (his-45), F08G2.3 (his-42), F17E9.10 (his-32), K06C4.13 (his-27), ZK131.2 (his-25), K06C4.5 (his-17), ZK131.7 (his-13), ZK131.3 (his-9), F45F2.13 (his-6), T10C6.13 (his-2).
H3.3 is encoded by 2 genes: F45E1.6 (his-71), Y49E10.6 (his-72)

The numbering for *C. elegans* homologs shown is based on the alignment for the S-phase class of proteins.

```
                 ---------1---------2---------3---------4---------5---------
S-phase         MARTKQTARKSTGGKAPRKQLATKAARKSAPASGGVKKPHRYRPGTVALREIRRYQKSTE 60
HIS-74          MARTKQTARKSTGGKAPRKALATKAARKSAIVTGSVKKVHRFRPGTVALREIRRYQKSTE
H3.3-71         MARTKQTARKSTGGKAPRKQLATKAARKSAPTTGGVKKPHRYRPGTVALREIRRYQKSTE 60
H3.3-72         MARTKQTARKSTGGKAPRKQLATKAARKSAPTTGGVKKPHRYRPGTVALREIRRYQKSTE 60
HIS-70          MARTKHTARKSFGGKAPRKSLATKAARKVFPVDGQVKK--RYRPSSNALKEIRKYQKSTE 58
HIS-73          MVPHQRTTVRSIMN-QLEKQLQAGSRRNAAP----EETNPSCTGENSEFSTTKNSLQTLN 55
HIS-69          ---------MCPGGKAPRKQLATKAARKNAIVVGAVKKPHRFRPGTVALREIRRYQKSTD 51
Mouse H3        MARTKQTARKSTGGKAPRKQLATKAARKSAPATGGVKKPHRYRPGTVALREIRRYQKSTE
                 ---------1---------2---------3---------4---------5---------
                          .  .   .* * : : *:        :.       .  :   :.  :: :

                6---------7---------8---------9---------0---------1---------
S-phase         LLIRRAPFQRLVREIAQDFKTDLRFQSSAVMALQEAAEAYLVGLFEDTNLCAIHAKRVTI 120
CeH3(his-74)    LLLRKLPFQRLVREIAQDFKTDLRFQSAAIGALQEASEAYLVGLFEDTNLCAIHAKRVTI
H3.3-71         LLIRKLPFQRLVREIAQDFKTDLRFQSAAIGALQEASEAYLVGLFEDTNLCAIHAKRVTI 120
H3.3-72         LLIRKLPFQRLVREIAQDFKTDLRFQSAAIGALQEASEAYLVGLFEDTNLCAIHAKRVTI 120
HIS-70          LLVRKLPFQRLVREVAQEIMPNVRFQSAAIQALHEAAEAYLIGLFEDTNLCAIHAKRVTI 118
HIS-73          LLIRRAPFQRLVREIAQDFKTDLRFQSSAVMALQEAAEAYLVGLLEDTNLCAIHAKRVTI 115
HIS-69          LLLRKLPFQRLVREIAQDVKQDLRFQSAAIQALQEASEYFLVGLFEDTNLCAIHAKRVTI 111
Mouse H3        LLIRKLPFQRLVREIAQDFKTDLRFQSSAVMALQEACEAYLVGLFEDTNLCAIHAKRVTI
                6---------7---------8---------9---------0---------1---------
                **:*: ********:**:.  ::****:*: **:**.* :*:**:***************

                2---------3-----
S-phase         MPKDIQLARRIRGERA 136
CeH3(his-74)    MPKDMQLARRIRGERS
H3.3-71         MPKDIQLARRIRGERA 136
H3.3-72         MPKDMQLARRIRGERA 136
HIS-70          MPKDMQVG-------- 126
HIS-73          MPKDIQLARRIRGERA 131
HIS-69          MPKDMQLARRIRGERN 127
Mouse H3        MPKDIQLARRIRGERA
                2---------3-----
                ****:*:.
```
