# Supplementary material for: The Specification and Global Reprogramming of Histone Epigenetic Marks during Gamete Formation and Early Embryo Development in C. elegans
Source: PLoS Genet. 2014 Oct 9;10(10):e1004588. doi: 10.1371/journal.pgen.1004588 (PMC4191889; doi:10.1371/journal.pgen.1004588)
Supplement: Table S8 — The status of paternal histone modification marks identified and tracked in the embryo in this study compared with mouse. (DOCX) [file pgen.1004588.s021.docx]

|  |  |  | ***C. elegans*** | **Mouse** | **References** |
| --- | --- | --- | --- | --- | --- |
| H2A | K119 | ub | erased | erased | 1-5 |
| H2B | K12 | ac | erased | unknown |  |
| H3 | K27 | ac | erased | unknown |  |
|  | K79 | me3 | erased | erased | 6 |
| H4 | K5 | ac | erased | erased | 7 |
|  | K12 | ac | erased | retained | 7, 8, 9 |
|  | K16 | ac | erased | retained | 7, 8 |
|  |  |  |  |  |  |
| H3 | K23 | me1 | retained | unknown |  |
|  | K36 | me1 | retained | retained | 8 |
|  | K79 | me2 | retained | erased | 6 |
| H4 | K8 | ac | retained | retained | 7, 8 |
|  | K20 | me1 | retained | retained | 10 |

**References:**

1. Rathke C, Baarends WM, Jayaramaiah-Raja S, Bartkuhn M, Renkawitz R, et al. (2007) Transition from a nucleosome-based to a protamine-based chromatin configuration during spermiogenesis in *Drosophila*. J Cell Sci 120: 1689-1700.

2. Baarends WM, Hoogerbrugge JW, Roest HP, Ooms M, Vreeburg J, et al. (1999) Histone ubiquitination and chromatin remodeling in mouse spermatogenesis. Dev Biol 207: 322-333.

3. Lu LY, Wu J, Ye L, Gavrilina GB, Saunders TL, et al. (2010) RNF8-dependent histone modifications regulate nucleosome removal during spermatogenesis. Dev Cell 18: 371-384.

4. Chen HY, Sun JM, Zhang Y, Davie JR, Meistrich ML (1998) Ubiquitination of histone H3 in elongating spermatids of rat testes. J Biol Chem 273: 13165-13169.

5. Sin HS, Barski A, Zhang F, Kartashov AV, Nussenzweig A, et al. (2012) RNF8 regulates active epigenetic modifications and escape gene activation from inactive sex chromosomes in post-meiotic spermatids. Genes Dev 26: 2737-2748.

6. Vielle A, Lang J, Dong Y, Ercan S, Kotwaliwale C, et al. (2012) H4K20me1 contributes to downregulation of X-linked genes for *C. elegans* dosage compensation. PLoS Genet 8: e1002933.

7. van der Heijden GW, Derijck AA, Ramos L, Giele M, van der Vlag J, et al. (2006) Transmission of modified nucleosomes from the mouse male germline to the zygote and subsequent remodeling of paternal chromatin. Dev Biol 298: 458-469.

8. Brunner AM, Nanni P, Mansuy IM (2014) Epigenetic marking of sperm by post-translational modification of histones and protamines. Epigenetics Chromatin 7: 2.

9. Paradowska AS, Miller D, Spiess AN, Vieweg M, Cerna M, et al. (2012) Genome wide identification of promoter binding sites for H4K12ac in human sperm and its relevance for early embryonic development. Epigenetics 7: 1057-1070.

10. van der Heijden GW, Dieker JW, Derijck AA, Muller S, Berden JH, et al. (2005) Asymmetry in histone H3 variants and lysine methylation between paternal and maternal chromatin of the early mouse zygote. Mech Dev 122: 1008-1022.
